# Diretriz Brasileira de Reabilitação Cardiovascular – 2020

**DOI:** 10.36660/abc.20200407

**Published:** 2020-05-22

**Authors:** Tales de Carvalho, Mauricio Milani, Almir Sergio Ferraz, Anderson Donelli da Silveira, Artur Haddad Herdy, Carlos Alberto Cordeiro Hossri, Christina Grüne Souza e Silva, Claudio Gil Soares de Araújo, Eneas Antonio Rocco, José Antonio Caldas Teixeira, Luciana Oliveira Cascaes Dourado, Luciana Diniz Nagem Janot de Matos, Luiz Gustavo Marin Emed, Luiz Eduardo Fonteles Ritt, Marconi Gomes da Silva, Mauro Augusto dos Santos, Miguel Morita Fernandes da Silva, Odilon Gariglio Alvarenga de Freitas, Pablo Marino Corrêa Nascimento, Ricardo Stein, Romeu Sergio Meneghelo, Salvador Manoel Serra

**Affiliations:** 1 Clínica de Prevenção e Reabilitação Cardiosport FlorianópolisSC Brasil Clínica de Prevenção e Reabilitação Cardiosport , Florianópolis , SC – Brasil; 2 Universidade do Estado de Santa Catarina FlorianópolisSC Brasil Universidade do Estado de Santa Catarina (Udesc), Florianópolis , SC – Brasil; 3 Fitcordis Medicina do Exercício BrasíliaDF Brasil Fitcordis Medicina do Exercício , Brasília , DF – Brasil; 4 Instituto Dante Pazzanese de Cardiologia São PauloSP Brasil Instituto Dante Pazzanese de Cardiologia , São Paulo , SP – Brasil; 5 Programa de Pós-Graduação em Cardiologia e Ciências Cardiovasculares Universidade Federal do Rio Grande do Sul Porto AlegreRS Brasil Programa de Pós-Graduação em Cardiologia e Ciências Cardiovasculares da Universidade Federal do Rio Grande do Sul (UFRGS), Porto Alegre , RS – Brasil; 6 Hospital de Clínicas de Porto Alegre Universidade Federal do Rio Grande do Sul Porto AlegreRS Brasil Hospital de Clínicas de Porto Alegre , Universidade Federal do Rio Grande do Sul (HCPA/UFRGS), Porto Alegre , RS – Brasil; 7 Vitta Centro de Bem Estar Físico Porto AlegreRS Brasil Vitta Centro de Bem Estar Físico , Porto Alegre , RS – Brasil; 8 Instituto de Cardiologia de Santa Catarina FlorianópolisSC Brasil Instituto de Cardiologia de Santa Catarina , Florianópolis , SC – Brasil; 9 Universidade do Sul de Santa Catarina FlorianópolisSC Brasil Unisul: Universidade do Sul de Santa Catarina (UNISUL), Florianópolis , SC – Brasil; 10 Hospital do Coração São PauloSP Brasil Hospital do Coração (Hcor), São Paulo , SP – Brasil; 11 Clínica de Medicina do Exercício Rio de JaneiroRJ Brasil Clínica de Medicina do Exercício , Clinimex, Rio de Janeiro , RJ – Brasil; 12 Hospital Samaritano Paulista São PauloSP Brasil Hospital Samaritano Paulista , São Paulo , SP – Brasil; 13 Universidade Federal Fluminense Rio de JaneiroRJ Brasil Universidade Federal Fluminense (UFF), Rio de Janeiro , RJ – Brasil; 14 Instituto do Coração Hospital das Clínicas da Faculdade de Medicina Universidade de São Paulo Rio de JaneiroRJ Brasil Instituto do Coração (Incor) do Hospital das Clínicas da Faculdade de Medicina da Universidade de São Paulo (HC-FMUSP), Rio de Janeiro , RJ – Brasil; 15 Hospital Israelita Albert Einstein São PauloSP Brasil Hospital Israelita Albert Einstein , São Paulo , SP – Brasil; 16 Hospital Cardiológico Costantini CuritibaPR Brasil Hospital Cardiológico Costantini , Curitiba , PR – Brasil; 17 Hospital Cárdio Pulmonar SalvadorBA Brasil Hospital Cárdio Pulmonar , Salvador , BA – Brasil; 18 Escola Bahiana de Medicina e Saúde Pública SalvadorBA Brasil Escola Bahiana de Medicina e Saúde Pública , Salvador , BA – Brasil; 19 SPORTIF – Clínica do Exercício do Esporte Belo HorizonteMG Brasil SPORTIF – Clínica do Exercício do Esporte , Belo Horizonte , MG – Brasil; 20 ACE Cardiologia do Exercício Rio de JaneiroRJ Brasil ACE Cardiologia do Exercício , Rio de Janeiro , RJ – Brasil; 21 Instituto Nacional de Cardiologia Rio de JaneiroRJ Brasil Instituto Nacional de Cardiologia , Rio de Janeiro , RJ – Brasil; 22 Universidade Federal do Paraná CuritibaPR Brasil Universidade Federal do Paraná , Curitiba , PR – Brasil; 23 Minascor Centro Médico Belo HorizonteMG Brasil Minascor Centro Médico , Belo Horizonte , MG – Brasil; 24 Instituto Estadual de Cardiologia Aloysio de Castro Rio de JaneiroRJ Brasil Instituto Estadual de Cardiologia Aloysio de Castro (IECAC), Rio de Janeiro , RJ – Brasil

**Realização:** *Departamento de Ergometria, Exercício, Cardiologia Nuclear e Reabilitação Cardiovascular da Sociedade Brasileira de Cardiologia*

**Conselho de Normatizações e Diretrizes (2020-2021):** *Brivaldo Markman Filho, Antonio Carlos Sobral Sousa, Aurora Felice Castro Issa, Bruno Ramos Nascimento, Harry Correa Filho, Marcelo Luiz Campos Vieira*

**Coordenador de Normatizações e Diretrizes (2020-2021):** *Brivaldo Markman Filho*

**Coordenadores da Diretriz:** *Tales de Carvalho e Mauricio Milani*

**Table:** 

Declaração de potencial conflito de interesses dos autores/colaboradores da Diretriz Brasileira de Reabilitação Cardiovascular – 2020 Se nos últimos 3 anos o autor/colaborador da diretriz:							

Nomes Integrantes da diretriz	Participou de estudos clínicos e/ou experimentais subvencionados pela indústria farmacêutica ou de equipamentos relacionados à diretriz em questão	Foi palestrante em eventos ou atividades patrocinadas pela indústria relacionados à diretriz em questão	Foi (é) membro do conselho consultivo ou diretivo da indústria farmacêutica ou de equipamentos	Participou de comitês normativos de estudos científicos patrocinados pela indústria	Recebeu auxílio pessoal ou institucional da indústria	Elaborou textos científicos em periódicos patrocinados pela indústria	Tem ações da indústria
Almir Sergio Ferraz	Novartis, Amgen, Sanofi	Não	Não	Não	Não	Boehringer	Não
Anderson Donelli da Silveira	Não	Não	Não	Não	Não	Não	Não
Artur Haddad Herdy	Não	Não	Não	Não	Não	Não	Não
Carlos Alberto Cordeiro Hossri	Não	Não	Não	Não	Não	Não	Não
Christina Grüne Souza e Silva	Não	Não	Não	Não	Não	Não	Não
Claudio Gil Soares de Araújo	Não	Não	Não	Não	Inbramed	Não	Não
Eneas Antonio Rocco	Não	Não	Não	Não	Não	Não	Não
José Antonio Caldas Teixeira	Não	Não	Não	Não	Não	Não	Não
Luciana Diniz Nagem Janot de Matos	Não	Não	Não	Não	Não	Não	Não
Luciana Oliveira Cascaes Dourado	Não	Não	Não	Não	Não	Não	Não
Luiz Gustavo Marin Emed	Não	Não	Não	Não	Não	Não	Não
Luiz Eduardo Fonteles Ritt	Não	Não	Não	Não	Não	Não	Não
Marconi Gomes da Silva	Não	Não	Não	Não	Não	Não	Não
Mauricio Milani	Não	Não	Não	Não	Não	Não	Não
Mauro Augusto dos Santos	Não	Não	Não	Não	Não	Não	Não
Miguel Morita Fernandes da Silva	Não	Não	Não	Não	Novartis	Não	Não
Odilon Gariglio Alvarenga de Freitas	Não	Não	Não	Não	Não	Não	Não
Pablo Marino Corrêa Nascimento	Não	Não	Não	Não	Não	Não	Não
Ricardo Stein	Não	Não	Não	Não	Não	Não	Não
Romeu Sergio Meneghelo	Não	Não	Não	Não	Não	Não	Não
Salvador Manoel Serra	Não	Não	Não	Não	Não	Não	Não
Tales de Carvalho	Não	Não	Não	Não	Não	Não	Não

Sumário

1. Introdução 946

1.1. Classes (Graus) de Recomendação 947

1.2. Níveis de Evidência 947

2. Estrutura de um Programa de Reabilitação Cardiovascular 947

2.1. Equipe e Responsabilidades dos Profissionais 947

**2.1.1. Médico Assistente 947**

**2.1.2. Médico-líder no Programa de Reabilitação Cardiovascular 947**

**2.1.3. Outros Profissionais 947**

**2.1.4. Fisioterapeutas e Profissionais de Educação Física 947**

**2.1.5. Profissional de Enfermagem 947**

2.2. Estrutura Física de um Serviço de Reabilitação 948

**2.2.1. Aspectos Gerais 948**

**2.2.2. Equipamentos para a Prática de Exercícios Físicos 948**

***2.2.2.1. Exercícios Aeróbicos* 948**

***2.2.2.2. Exercícios de Fortalecimento Muscular* 948**

***2.2.2.3. Outros Exercícios* 948**

**2.2.3. Monitoramento 948**

**2.2.4. Segurança 948**

3. Fases da Reabilitação Cardiovascular e Estratificação de Risco 949

3.1. Risco Clínico Alto 949

3.2. Risco Clínico Intermediário 950

3.3. Risco Clínico Baixo 951

4. Custo-efetividade da Reabilitação Cardiovascular 952

5. Reabilitação Cardiovascular Domiciliar 953

6. Reabilitação Cardiovascular Integrando o Tratamento Clínico Pleno das Doenças Cardiovasculares 953

6.1. Recomendações Gerais para Incremento da Atividade Física e Prática de Exercícios Físicos 954

6.2. Hipertensão Arterial Sistêmica 955

**6.2.1. Benefícios Terapêuticos dos Exercícios Físicos 955**

**6.2.2. Indicações de Exercícios Físicos na Hipertensão Arterial Sistêmica 956**

**6.2.3. Avaliação Pré-participação 956**

**6.2.4. Particularidades na Prescrição e no Acompanhamento dos Exercícios Físicos 957**

6.3. Coronariopatia Estável após Evento Agudo ou Revascularizações 957

**6.3.1. Benefícios Terapêuticos dos Exercícios Físicos 958**

**6.3.2. Quando Indicar Reabilitação 958**

**6.3.3. Avaliação Pré-participação e Prescrição de Exercícios 959**

**6.3.4. Particularidades na Prescrição e Acompanhamento dos Exercícios Físicos 960**

***6.3.4.1. Angina Refratária* 960**

***6.3.4.2. Treinamento com Indução de Isquemia Miocárdica* 960**

***6.3.4.3. Ajustes de Fármacos Diante da Assimilação do Treinamento Físico* 960**

6.4. Insuficiência Cardíaca 961

**6.4.1. Prescrição dos Exercícios Físicos e Avaliação Pré-participação 961**

**6.4.2. Considerações Finais sobre a Insuficiência Cardíaca 963**

6.5. Transplante Cardíaco 963

**6.5.1. Benefícios dos Exercícios Físicos 963**

**6.5.2. Avaliação Pré-participação e Particularidades 964**

**6.5.3. Prescrição do Treinamento Físico 964**

**6.5.4. Reabilitação Cardiovascular Domiciliar 965**

**6.5.5. Recomendações 965**

6.6. Miocardiopatias 966

**6.6.1. Miocardiopatia Hipertrófica 966**

***6.6.1.1. Benefícios Terapêuticos do Exercício Físico* 966**

***6.6.1.2. Quando Indicar Exercícios Físicos* 967**

***6.6.1.3. Avaliação Pré-participação* 967**

***6.6.1.4. Particularidades na Prescrição e no Acompanhamento dos Exercícios Físicos* 968**

**6.6.2. Miocardite 968**

**6.6.3. Outras Miocardiopatias 969**

***6.6.3.1. Cardiomiopatia Arritmogênica do Ventrículo Direito* 969**

***6.6.3.2. Miocardiopatia Não Compactada* 969**

6.7. Valvopatias 970

**6.7.1. Fase Pré-intervenção 970**

**6.7.2. Fase Pós-intervenção 970**

**6.7.3. Avaliação Pré-participação 970**

**6.7.4. Particularidades na Prescrição e no Acompanhamento dos Exercícios Físicos 971**

6.8. Portadores de Marcapasso ou Cardioversor Desfibrilador Implantável 971

**6.8.1. Benefícios Terapêuticos dos Exercícios Físicos 972**

**6.8.2. Quando Indicar Reabilitação Cardiovascular 973**

**6.8.3. Avaliação Pré-participação 973**

**6.8.4. Particularidades na Prescrição e no Acompanhamento dos Exercícios Físicos 973**

**6.8.5. Treinamento Resistido 974**

**6.8.6. Estimulação Elétrica Neuromuscular 974**

6.9. Doença Arterial Obstrutiva Periférica 975

Referências 977

## 1. Introdução

Está cientificamente comprovado, sendo algo incorporado ao senso comum, que ser fisicamente ativo contribui para preservar e recuperar a boa saúde do corpo e da mente. Os efeitos favoráveis da reabilitação cardiovascular (RCV) com ênfase nos exercícios físicos têm sido consistentemente documentados, inclusive em meta-análises de estudos clínicos randomizados, que demonstram significativas reduções da morbimortalidade cardiovascular e global, ^1^ bem como da taxa de hospitalização, ^1 , 2^ com expressivo ganho de qualidade de vida, ^1 , 2^ justificando a sua consensual e enfática recomendação pelas principais sociedades médicas mundiais. ^3 - 6^

O sedentarismo, que apresenta elevada prevalência no Brasil e no mundo, está fortemente relacionado às doenças cardiovasculares (DCV) e à mortalidade precoce. ^7 , 8^ Em contrapartida, maiores volumes de atividade física são positivamente associados à melhor qualidade e à maior expectativa de vida, ^9 - 13^ existindo uma forte e inversa associação dos diferentes componentes da aptidão física com a mortalidade por todas as causas e com a ocorrência de eventos cardiovasculares desfavoráveis. Ou seja, quanto menor o nível de aptidão física, maior tende ser a taxa de mortalidade. ^14 - 21^

Portanto, o principal objetivo da RCV com ênfase nos exercícios físicos é propiciar uma melhora dos componentes da aptidão física, tanto aeróbico quanto não aeróbicos (força/potência muscular, flexibilidade, equilíbrio), algo que exige a combinação de diferentes modalidades de treinamento. Assim, a RCV deve proporcionar os mais elevados níveis de aptidão física passíveis de obtenção, de modo a reduzir o risco de eventos cardiovasculares e promover todos os outros benefícios a serem auferidos pela prática regular de exercícios físicos, culminando com a redução da mortalidade geral. ^14 - 21^

Entretanto, apesar dos benefícios documentados e do excelente significado em termos de custo-efetividade, ^22 , 23^ a RCV é mundialmente subutilizada. No Brasil, país de dimensão continental e grande diversidade social e econômica, dentre as inúmeras barreiras ao acesso à RCV, ^24 , 25^ vale destacar como algo presente em praticamente todas as regiões: escassez de serviços estruturados, dificuldade de deslocamento (mobilidade urbana ruim) e níveis altos de violência nas cidades. ^26 , 27^ Neste contexto, programas de reabilitação cardiovascular domiciliar (RCVD), em que a maioria das sessões ocorre no ambiente domiciliar sob supervisão indireta, surgem como complemento ou alternativa aos programas tradicionais, nos quais as sessões são sempre realizadas sob supervisão direta ou presencial.

A exemplo do que ocorreu nos documentos anteriormente publicados pela Sociedade Brasileira de Cardiologia sobre o tema, ^6 , 28 - 31^ esta diretriz aborda exclusivamente a intervenção com base na prática de exercícios físicos direcionadas aos pacientes com DCV, sendo a classe (ou grau) de recomendação sempre fundamentada no nível de evidência encontrado, conforme consta a seguir.

### 1.1. Classes (Graus) de Recomendação

- **Classe I**: condições para as quais há evidências conclusivas, ou, na sua falta, consenso de que o procedimento é seguro e útil/eficaz;
- **Classe II**: condições para as quais há evidências conflitantes e/ou divergência de opinião sobre segurança e utilidade/eficácia do procedimento:
- **Classe IIA**: peso ou evidência/opinião a favor do procedimento. A maioria aprova;
- **Classe IIB**: segurança e utilidade/eficácia menos bem estabelecida, não havendo predomínio de opiniões a favor.
- **Classe III**: condições para as quais há evidências e/ou consenso de que o procedimento não é útil/eficaz e, em alguns casos, pode ser prejudicial.

### 1.2. Níveis de Evidência

- **Nível A**: dados obtidos a partir de múltiplos estudos randomizados de bom porte, concordantes e/ou de meta-análise robusta de estudos clínicos randomizados;
- **Nível B**: dados obtidos a partir de meta-análise menos robusta, com base em um único estudo randomizado ou em estudos não randomizados (observacionais);
- **Nível C**: dados obtidos de opiniões consensuais de especialistas.

## 2. Estrutura de um Programa de Reabilitação Cardiovascular

### 2.1. Equipe e Responsabilidades dos Profissionais

A composição das equipes profissionais de RCV deve ajustar-se aos objetivos, à clientela e às disponibilidades de recursos humanos e materiais, respeitadas as características regionais, a modalidade (supervisão direta ou indireta) e o local de realização (hospital, clínica, ambulatório e outros). A equipe multiprofissional habitualmente é composta por médicos, educadores físicos, fisioterapeutas e profissionais de enfermagem, mas outros, como nutricionistas, psicólogos e assistentes sociais, podem compor a equipe. ^31 , 32^

#### 2.1.1. Médico Assistente

A RCV compõe o tratamento clínico pleno dos pacientes estáveis com DCV, o que exige a integração do médico assistente, que, ao encaminhar o seu paciente, deve ter conhecimento das indicações e dos benefícios a serem obtidos, adotando as necessárias providências clínicas pré-participação. Tendo em vista o encaminhamento de relatórios, eventuais necessidades de ajustes farmacológicos, intercorrências médicas, entre outros, é de grande relevância que sejam criados mecanismos para uma fácil comunicação entre o médico assistente e a equipe de RCV. ^31^

#### 2.1.2. Médico-líder no Programa de Reabilitação Cardiovascular

Coordena as ações médicas, sendo no Brasil habitualmente o coordenador geral do programa de RCV. Ele deve conhecer em profundidade a temática de RCV e ter conhecimento para atuar em emergências cardiovasculares. ^6 , 32 - 34^

Algumas de suas principais atuações são:

- Executar a avaliação pré-participação, com inclusão de testes de exercício, de modo a subsidiar a programação inicial das sessões de treinamento da RCV; ^31^
- Treinar a equipe para identificar situações de risco e realizar o atendimento apropriado em situações emergenciais;
- Estabelecer restrições e limites para a prescrição dos exercícios físicos;
- Liderar e interagir com os demais membros da equipe, com o objetivo de otimizar a qualidade e a segurança da prescrição dos exercícios físicos;
- Programar reavaliações subsequentes, sempre interagindo com o médico assistente.

#### 2.1.3. Outros Profissionais

De modo semelhante aos médicos, os demais membros da equipe, ao executarem suas respectivas funções, devem seguir as normas e regras que norteiam as atividades do programa, respeitando as recomendações de seus respectivos conselhos profissionais. ^31^

#### 2.1.4. Fisioterapeutas e Profissionais de Educação Física

Atuam diretamente na prescrição e na supervisão dos exercícios físicos, dentro das metas e dos limites definidos na orientação médica, após a avaliação pré-participação e subsequentes reavaliações. Devem ter conhecimentos específicos sobre as DCV e fisiologia do exercício, além de receberem periodicamente treinamento de suporte básico de vida, incluindo o uso de desfibrilador automático externo. Além da atuação nas sessões de exercícios físicos, podem contribuir para as orientações e demais medidas, visando a adoção de hábitos saudáveis.

#### 2.1.5. Profissional de Enfermagem

Em um programa de RCV, o profissional de enfermagem pode auxiliar na avaliação clínica, atuando na obtenção e no fornecimento de informações relacionadas à situação clínica do paciente, inclusive em contato com os familiares. Pode ser responsável pelas dosagens de glicemia e verificação de pressão arterial (PA), antes e durante as sessões de exercícios. Em caso de intercorrências clínicas, pode participar do atendimento e auxiliar o médico, com eventuais administrações de medicamentos. Deve também estar capacitado para atuar no suporte básico de vida, com uso de desfibrilador automático externo.

## 2.2. Estrutura Física de um Serviço de Reabilitação

### 2.2.1. Aspectos Gerais

Um programa de RCV pode funcionar em vários tipos de instalações, a depender dos objetivos e recursos disponíveis. Mais frequentemente, os programas de RCV são realizados em ambientes fechados e climatizados, sendo também possível realizar as sessões de exercícios físicos em espaços abertos, como pistas de atletismo, quadras, ginásios poliesportivos, parques ou áreas públicas de lazer. ^29^

Em ambientes fechados, o espaço para a realização dos exercícios físicos deverá apresentar dimensões e características adequadas, variáveis de acordo com os recursos locais e a capacidade de atendimento. O ambiente deverá ser suficientemente amplo para a realização dos exercícios físicos, com uma altura de pé direito idealmente igual ou superior a 2,5 m. Também deverá ser apropriadamente iluminado e bem ventilado, onde seja possível manter a temperatura entre 22 ^o^ C e 25 ^o^ C, além de umidade relativa do ar entre 40 e 65% durante as sessões de exercício. A área disponível exclusivamente para a realização dos exercícios físicos, desconsiderando vestiários, banheiros, recepção ou sala de espera, varia muito, podendo ir desde 20 m ^2^ a algumas centenas de metros quadrados. É importante que existam locais próprios para a troca de roupas e instalações sanitárias. Para minimizar o risco de acidentes e quedas, o piso deve ter propriedades antiderrapantes. ^29^

### 2.2.2. Equipamentos para a Prática de Exercícios Físicos

***2.2.2.1. Exercícios Aeróbicos***

Os equipamentos mais usados são esteiras rolantes e cicloergômetros de membros inferiores (MMII), mas também podem ser utilizados cicloergômetros de membros superiores (MMSS), remoergômetros, ergômetros de esqui, elípticos, entre outros. ^29^

As esteiras rolantes devem ser elétricas, com capacidade de suportar, pelo menos, 100 kg de peso corporal, com suportes frontal e lateral para as mãos e trava de segurança. Devem também permitir ajuste individualizado dentro de uma faixa ampla de velocidade e inclinação. Os cicloergômetros podem ser de frenagem mecânica ou eletromagnética. Há modelos específicos para MMSS ou ainda para que os quatro membros sejam exercitados simultaneamente. Para os modelos de MMII existem as opções vertical e horizontal. O ideal é que o cicloergômetro possibilite a leitura da cadência ou velocidade e, principalmente, da potência em watts. Há cicloergômetros em que é possível programar a intensidade diretamente em watts, de modo que a resistência do pedal aumenta quando a cadência diminui e vice-versa.

Os remoergômetros, ergômetros de esqui e elípticos podem ser particularmente úteis para os pacientes com menor grau de limitação funcional ou que já tenham tido experiências prévias com tais equipamentos. Eles apresentam como vantagem possibilitar o exercício simultâneo dos MMSS e MMII.

***2.2.2.2. Exercícios de Fortalecimento Muscular***

Há vários tipos de equipamentos que podem ser utilizados para o fortalecimento muscular. Porém, é possível realizar vários exercícios utilizando somente o peso corporal, que representa um esforço, em geral, suficiente nos pacientes mais debilitados. Um exemplo prático é o exercício de sentar e levantar, cuja realização requer tão somente uma cadeira ou um banco.

O uso de cordas ou faixas suspensas, bem fixadas ao teto ou alto da parede, podem permitir uma ampla variedade de exercícios com a utilização do peso do próprio corpo. Pesos livres, halteres ou caneleiras com pesos variados são frequentemente adotados em programas de RCV e possibilitam uma ampla variedade de movimentos e estímulos adequados de diferentes grupos musculares. Podem ser também utilizados aparelhos específicos, com pesos ligados a cabos e polias. Outros equipamentos que também podem ser usados: barras, bastões, bolas com peso ( *medicine balls* ), “bolas suíças” e faixas ou bandas elásticas com diferentes graus de resistência. ^29^

Em todos os exercícios deve haver atenção para a correta execução dos movimentos e adequada postura, a fim de evitar lesões osteomusculares. Atenção ao manuseio dos equipamentos também é necessária, com o intuito de evitar acidentes com o material e eventuais lesões corporais.

***2.2.2.3. Outros Exercícios***

Visando a saúde global, considerando a cardiopatia e doenças associadas, pode ser necessário acrescentar outros tipos de exercícios, como treinamento isométrico manual, treinamento da musculatura inspiratória e exercícios para aprimorar o equilíbrio e a flexibilidade.

### 2.2.3. Monitoramento

Além de esfigmomanômetros e estetoscópios, há vários recursos disponíveis, como cardiofrequencímetros e aplicativos de celulares para monitoramento da frequência cardíaca (FC), glicosímetros e oxímetros digitais. Dependendo da complexidade clínica e do risco de eventos cardiovasculares desfavoráveis, é desejável o monitoramento eletrocardiográfico no repouso e durante o exercício, que pode ser obtido por equipamentos de conexão direta ao paciente ou por sistemas de telemetria, sendo de fundamental importância em caso de eventos cardiovasculares a possibilidade do rápido acesso aos equipamentos, para identificação do quadro clínico e a subsequente conduta médica.

### 2.2.4. Segurança

Apesar de ser extremamente incomum, é importante que o programa tenha um planejamento para o adequado atendimento de eventos cardiovasculares graves, como a parada cardiorrespiratória, que, na maioria dos casos em adultos, decorre de fibrilação ventricular ou taquicardia ventricular sem pulso. Portanto, o desfibrilador, manual ou automático, é um equipamento de segurança obrigatório. Ainda devem estar disponíveis outros materiais do suporte básico e avançado de vida, como laringoscópio, tubos orotraqueais de tamanhos variados, máscaras, ambu e oxigenioterapia suplementar.

Para orientação mais detalhada de técnicas, equipamentos e medicamentos, orienta-se consultar diretrizes específicas sobre os respectivos assuntos. ^35 , 36^

## 3. Fases da Reabilitação Cardiovascular e Estratificação de Risco

Tradicionalmente, a RCV é dividida em fases temporais, sendo a fase 1 intra-hospitalar e as fases 2 a 4 ambulatoriais. Nos primórdios, a fase 1 foi destinada à recuperação após infarto agudo do miocárdio (IAM) ou cirurgia de revascularização miocárdica (CRVM). Posteriormente, em contexto atualmente denominado reabilitação cardiopulmonar e metabólica, foram incluídos os pacientes internados submetidos a intervenções coronárias percutâneas (ICP), cirurgias valvares, cirurgias para cardiopatias congênitas e transplante cardíaco (TxC), além dos portadores de insuficiência cardíaca (IC), doença arterial coronariana (DAC), diabéticos, hipertensos, pneumopatas e nefropatas crônicos, assim que estabilizados clinicamente. Portanto, a RCV deve ser iniciada imediatamente após o paciente ter sido considerado clinicamente compensado, como decorrência do tratamento clínico e/ou intervencionista. ^31^

Na fase 1 da RCV objetiva-se que o paciente tenha alta hospitalar com as melhores condições físicas e psicológicas possíveis, municiado de informações referentes ao estilo saudável de vida, em especial no que diz respeito ao exercício físico. Propõe-se a combinação de exercícios físicos de baixa intensidade, técnicas para o controle do estresse e programas de educação em relação aos fatores de risco e à cardiopatia. A equipe de atendimento deve ser composta por, pelo menos, médico, fisioterapeuta e enfermeiro, capacitados para atuar em RCV, que não precisam dedicar tempo integral ao programa de reabilitação, podendo exercer outras atividades no hospital. ^31^

O direcionamento às fases ambulatoriais da RCV deve ser realizado na alta da internação. A fase 2 começa imediatamente após a alta hospitalar e tem duração média de 3 meses. A fase 3 costuma ter duração de 3 a 6 meses e a fase 4 tem duração prolongada. Em todas as fases objetiva-se progressão dos benefícios da RCV ou, pelo menos, a manutenção dos ganhos obtidos.

Em uma divisão rígida da RCV em fases temporais, pode-se não levar em consideração que existem pacientes com cardiopatias graves, muito sintomáticos e debilitados, que permanecem por longo prazo em uma reabilitação “fase 2”, pois continuam requerendo a supervisão direta dos exercícios físicos, enquanto outros, de baixo risco, desde o início se enquadram em programas de fase 3 ou mesmo de fase 4, sendo potenciais candidatos a uma RCV domiciliar, em que a maioria das sessões ocorrem sob supervisão indireta, à distância. ^31^

**Figura 1:**
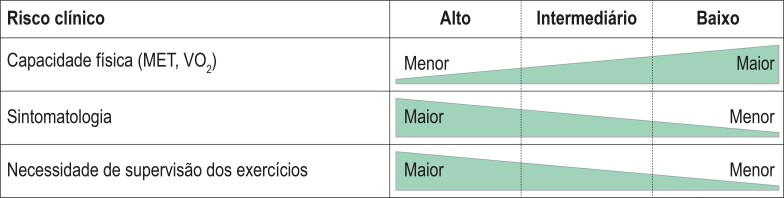
– Características gerais dos pacientes em reabilitação cardiovascular ambulatorial, de acordo com a estratificação do risco clínico. MET: equivalente metabólico; VO 2 : consumo de oxigênio.

Portanto, recomenda-se uma estratificação do risco clínico que possibilite o uso mais racional dos programas, com direcionamento individualizado às modalidades de RCV. Nesse contexto, os pacientes de alto risco, com menor capacidade física e mais sintomáticos, devem participar de sessões supervisionadas por tempo indeterminado, enquanto os de menor risco, com maior capacidade física e menos sintomáticos precocemente podem realizar, sem supervisão direta, exercícios mais intensos e diversificados ( Figura 1 ).

A estratificação de risco clínico do paciente em alto, intermediário ou baixo é pautada em recomendações prévias. ^4 , 28 , 37^ As notas de corte para o enquadramento são baseadas na opinião de especialistas (evidência nível C), o que possibilita modificações regionais de acordo com a experiência da equipe da RCV e com o julgamento clínico realizado na avaliação médica pré-participação e subsequentes reavaliações ( Tabela 1 ).

**Tabela 1:** – Estratificação do risco clínico dos pacientes em reabilitação cardiovascular ambulatorial

Risco	Alto	Intermediário	Baixo

Característica			
Evento cardiovascular, intervenção cardiovascular ou descompensação clínica	Inferior a 8 a 12 semanas	Superior a 12 semanas	Superior a 6 meses
Capacidade funcional	TE: < 5 MET TCPE: Weber C/D ou VO _2_ pico < 60% do predito	TE: 5 a 7 MET TCPE: Weber B ou VO _2_ pico de 60 a 85% do predito	TE: > 7 MET TCPE: Weber A ou VO _2_ pico > 85% do predito
Sinais e sintomas de isquemia miocárdica (limiar isquêmico)	Em baixas cargas TE: abaixo de 6 MET TCPE: abaixo de 15 ml.kg ^-1^ .min ^-1^	TE: acima de 6 MET TCPE: acima de 15 ml.kg ^-1^ .min ^-1^	Ausente
Sintomatologia	IC: CF III e IV Angina: CF III e IV	IC: CF I a II Angina: CF I e II	Ausente
Outras características clínicas	IRC dialítica; queda da saturação de oxigênio em esforço; arritmia ventricular complexa	De acordo com o julgamento clínico na avaliação médica pré-participação	De acordo com o julgamento clínico na avaliação médica pré-participação

*CF: classe funcional; IC: insuficiência cardíaca; IRC: insuficiência renal crônica; MET: equivalente metabólico; TCPE: teste cardiopulmonar de exercício; TE: teste ergométrico; VO _2_: consumo de oxigênio.*

### 3.1. Risco Clínico Alto

A duração da RCV pode variar conforme o quadro clínico e a evolução do treinamento físico. O enquadramento, a manutenção ou a reclassificação do perfil de risco devem ser determinados pela avaliação médica pré-participação e por reavaliações subsequentes, realizadas pelo médico e demais integrantes da equipe. O modelo dessa avaliação médica pode variar de acordo com a estrutura logística e a experiência do serviço, devendo conter, no mínimo, consulta clínica, exame físico, eletrocardiograma (ECG) de repouso e teste cardiopulmonar de exercício (TCPE) ou teste ergométrico (TE).

Os pacientes de alto risco, com frequência, podem necessitar de atendimento médico imediato ou a curto prazo (reinternação, intervenções ou ajustes de fármacos). Portanto, requerem maior monitoramento do treinamento pela equipe assistencial, a qual deve ser capaz de identificar sinais e sintomas de situações de risco e atuar no atendimento de intercorrências clínicas, inclusive com material de suporte básico e avançado de vida, com cardiodesfibrilador manual ou automático. É preferencial, inclusive, que esse equipamento esteja dentro da sala de atendimento. A equipe médica deve estar prontamente disponível na localidade, com rápido acesso ao paciente em caso de intercorrências graves.

Ressalte-se que a melhor maneira de prevenir intercorrências durante um programa de reabilitação e, especialmente após eventos e intervenções, consiste na realização de qualificadas avaliações pré-participação e subsequentes, que devem ser sistemáticas.

O programa de exercícios deve ser individualizado em termos de intensidade, duração, frequência, modalidade de treinamento e progressão, de acordo com os testes funcionais realizados inicialmente e no seguimento. Sempre devem ser adotados recursos para a correta determinação da FC e verificação da PA, em repouso e em esforço, além da possibilidade de verificação de saturação de oxigênio, determinação da glicemia capilar e monitoramento eletrocardiográfico.

O atendimento também deve contemplar um programa educacional direcionado à modificação do estilo de vida, com ênfase na reeducação alimentar e em estratégias para cessação do tabagismo, quando necessárias. É importante que o paciente obtenha conhecimentos sobre sua doença e aprendizado de automonitoramento, tanto na execução dos exercícios quanto na identificação de sinais e sintomas de alerta para situações clínicas instáveis ou de risco.

As características clínicas dos pacientes que se enquadrariam inicialmente no risco clínico alto (presença de, pelo menos, uma delas) são:

- Internação por descompensação cardiovascular recente (menos de 8 a 12 semanas) devido a quadros de: IAM ou angina instável; revascularização cirúrgica ou percutânea; arritmias complexas; morte súbita revertida; descompensação de IC;
- Pacientes cardiopatas, com presença ou ausência de evento cardiovascular e/ou intervenções, mas com importantes alterações funcionais ao esforço físico, ou seja:

**–** Baixa capacidade funcional no TE (menor que 5 equivalentes metabólicos [MET]) ou no TCPE (classificação de Weber C e D ou consumo de oxigênio [VO _2_ ] abaixo de 60% do predito para idade e sexo);
**–** Sinais e sintomas de isquemia miocárdica em baixa carga (abaixo de 6 MET ou de VO _2_ de 15 ml.kg ^-1^ .min ^-1^ );
**–** Sintomatologia exacerbada (IC com classe funcional III e IV ou angina classe funcional III e IV).

- Outras características clínicas de pacientes com risco aumentado aos exercícios físicos: doença renal crônica (DRC) dialítica, dessaturação de oxigênio em esforço e arritmia ventricular complexa em repouso ou esforço.

Considerando que os pacientes de alto risco frequentemente necessitam de reajustes de fármacos e de reavaliações, com eventuais intervenções (revascularizações ou outros procedimentos), torna-se essencial comunicação constante da equipe assistencial da RCV com o(s) médico(s) assistente(s). É importante destacar que alguns pacientes, devido a intercorrências nas sessões e/ou resultados nas avaliações subsequentes, podem permanecer classificados como de alto risco, mantendo a prática de exercícios físicos sob supervisão direta por tempo indeterminado.

### 3.2. Risco Clínico Intermediário

Os pacientes podem ter cumprido etapas anteriores da RCV, sendo reclassificados, ingressar diretamente nessa categoria sem participações prévias ou ser oriundos de outros programas de exercícios. A duração da RCV sob essa classificação também pode ser variável, a depender do quadro clínico e da evolução do treinamento físico, algo a ser definido nas reavaliações subsequentes.

A supervisão de exercícios deve ser feita pelo fisioterapeuta ou professor de educação física, e o serviço deve, idealmente, contar com a coordenação geral de um médico com experiência em RCV. É recomendada a disponibilidade de recursos para a correta determinação da FC e verificação de PA em repouso e esforço e, sempre que necessário, com possibilidade de verificação da saturação de oxigênio, determinação da glicemia e monitoramento eletrocardiográfico.

Para o atendimento de pacientes de risco intermediário, caso não haja médico presente no local das atividades, deve haver possibilidade do seu rápido acionamento remoto. A estrutura do serviço deve apresentar material de suporte básico de vida e profissionais de saúde treinados em reanimação cardiopulmonar, com o uso de desfibrilador automático externo, o qual deve estar presente no local de atendimento.

É fundamental que a equipe médica integrada ao serviço de RCV realize a avaliação pré-participação, com adequada estratificação do risco. O seguimento médico regular e as reavaliações sistemáticas, além dos atendimentos eventuais quando necessários, são fundamentais para garantir a segurança dos exercícios.

As características clínicas dos pacientes com risco intermediário (presença de, pelo menos, uma característica) são:

- Evento cardiovascular ou intervenções com intervalo superior a 12 semanas, com estabilidade do quadro clínico;
- Pacientes cardiopatas que ainda apresentam algumas alterações funcionais em esforço físico:

**–** Moderada capacidade funcional no TE (entre 5 e 7 MET) ou no TCPE (classificação de Weber B ou VO _2_ entre 60 e 85% do predito para idade e sexo);
**–** Sinais e sintomas de isquemia em carga acima de 6 MET ou com VO _2_ acima de 15 ml.kg ^-1^ .min ^-1^ ;
**–** Sintomatologia de menor magnitude (IC com classe funcional I e II ou angina classe funcional I e II).

- Outras características clínicas que o médico responsável pela avaliação pré-participação julgue como de risco intermediário aos exercícios físicos.

O principal objetivo da RCV neste perfil de risco ainda é o aprimoramento da aptidão física, tanto aeróbica quanto não aeróbica (força/potência muscular, flexibilidade, equilíbrio), com melhor controle da(s) doença(s). Deve ser considerada a necessidade de promoção de bem-estar, com melhora da qualidade de vida, além de outros procedimentos que contribuam para a redução do risco de complicações clínicas, como é o caso das estratégias para cessação do tabagismo, reeducação alimentar e controle de peso corporal. A ênfase na manutenção e adesão do tratamento farmacológico também é fundamental para evitar a progressão ou instabilização da DCV. A obtenção de conhecimentos sobre a própria doença, possibilitando melhor automonitoramento, aumentam a acurácia na identificação de sinais e sintomas relacionados à progressão da doença ou a situações clínicas instáveis, que podem requerer interrupção do programa de exercícios e reavaliações médicas.

Os pacientes desta categoria, após um período inicial de orientações e de obtenção de conhecimentos sobre os exercícios e o automonitoramento, podem adequar-se a uma RCV domiciliar, na qual a prática de exercício físico é realizada com supervisão indireta, sob a responsabilidade de profissionais do serviço. A avaliação das sessões, com reajustes na prescrição e esclarecimentos de dúvidas, deve ser feita de maneira sistemática, presencial ou virtualmente, conforme o caso.

### 3.3. Risco Clínico Baixo

Assim como os pacientes de risco intermediário, os de baixo risco podem ter sido reclassificados após cumprirem etapas anteriores da RCV, ingressarem diretamente nesta categoria sem participações prévias na RCV, ou serem oriundos de outros programas de exercícios físicos. A duração do treinamento destes pacientes é de longo prazo, visando a manutenção da saúde geral e obtenção dos maiores ganhos possíveis nos componentes da aptidão física, com o objetivo de alcançar ao máximo o potencial de saúde do indivíduo.

Dependendo da disponibilidade e das preferências individuais, os exercícios podem ser realizados sob supervisão presencial ou à distância. Porém, em virtude do menor risco clínico e da menor necessidade de supervisão, os pacientes deste estágio se enquadram perfeitamente em um modelo domiciliar, de modo que a equipe da RCV possa dedicar atenção assistencial presencial principalmente aos pacientes de maior risco clínico.

Os pacientes devem ter reavaliações médicas periódicas, realizadas pelo seu médico assistente e pela equipe da RCV, com TCPE ou TE, cuja periodicidade, a princípio, não deve exceder 12 meses. O objetivo das reavaliações médicas é reajustar a prescrição do treinamento e identificar eventual piora da doença ou sinais de risco para situações instáveis ou eventos cardiovasculares, possibilitando eventuais reajustes do tratamento farmacológico e/ou intervenções cirúrgicas ou percutâneas.

Os pacientes em RCVD devem ser periodicamente avaliados e orientados para a prática dos exercícios, ocasiões em que é recomendável a participação em algumas sessões supervisionadas de exercícios, especialmente para os menos experientes, possibilitando eventuais reajustes na prescrição e esclarecimento de dúvidas. É também recomendável a realização de consultas periódicas com a equipe da RCV, por meio de contatos virtuais e/ou telefônicos, pelo menos uma vez a cada seis meses, para estimular a adesão ao programa de exercícios físicos.

As características clínicas dos pacientes do estágio 4 (presença de todas as características a seguir) são:

- Evento cardiovascular ou intervenções com intervalo superior a 6 meses e estabilidade clínica;
- Pacientes cardiopatas que não apresentam alterações funcionais em esforço físico ou que estas sejam muito discretas quando presentes;
- Os pacientes nessa classificação costumam apresentar as seguintes características:

**–** Boa capacidade funcional no TE (superior a 7 MET) ou no TCPE (classificação de Weber A ou VO _2_ acima de 85% do predito para idade e sexo);
**–** Ausência de sinais e sintomas de isquemia miocárdica ou de outra sintomatologia anormal ao esforço físico.

## 4. Custo-efetividade da Reabilitação Cardiovascular

Segundo a Organização Mundial da Saúde, entre 2000 e 2016 o aumento mundial dos gastos com saúde no mundo foi maior do que o crescimento da economia global, chegando a 7,5 trilhões de dólares em 2016. ^38^ Em relação às DCV, 863 bilhões de dólares foram gastos mundialmente em 2010, estimando-se que em 2030 chegue a 1,04 trilhão de dólares. ^39^

No Brasil, onde quase 50% dos gastos com saúde são financiados pelo governo, ^40^ observa-se situação semelhante, pois as DCV constituem o grupo que ocasiona o maior gasto com internações no Sistema Único de Saúde, sendo a principal causa de aposentadorias por invalidez. ^41 - 45^ Em 2015, em relação às DCV, estima-se que o gasto público com internações hospitalares e consultas tenha sido superior a 5 bilhões de reais e o gasto por afastamentos temporários ou permanentes superior a 380 milhões de reais. ^40^

Portanto, o impacto econômico provocado pelas DCV, aliado à obrigatoriedade do uso consequente e racional de recursos financeiros, exige a implementação em larga escala de modelos de baixo custo, viabilizando o atendimento de maior número de pacientes. Em coronariopatas estáveis, a RCV é uma estratégia que, em termos de custo-efetividade, supera, com larga margem, procedimentos amplamente utilizados no país, tais como a intervenção coronariana percutânea (ICP). ^46 , 47^ Além disso, sua utilização em maior escala proporcionaria redução nos gastos com saúde, em decorrência da diminuição de novos eventos cardiovasculares, reinternações hospitalares e tratamentos intervencionistas. ^48 , 49^ Assim, sua disseminação deveria ser considerada uma estratégia de saúde pública prioritária.

A determinação da custo-efetividade, que se faz por análise combinada das consequências clínicas (efetividade) e do gasto financeiro do sistema de saúde, é fundamental para avaliar a pertinência da implementação em larga escala de determinado tratamento. ^50 - 52^ De acordo com Georgiou et al., ^53^ são consideradas medidas de excelente custo-efetividade as que exigem investimentos inferiores a 20.000 dólares para salvar uma vida por ano (VSA), sendo aceitáveis as que exigem investimentos entre 20.000 e 40.000 dólares e inaceitáveis aquelas que exigem investimentos acima de 40.000 dólares por VSA.

De acordo com os dados disponíveis entre 1985 e 2004, a RCV foi considerada uma intervenção com excelente relação de custo-efetividade, na medida em que a sua adição ao tratamento convencional resultou em um aumento de gastos de 2.193 a 28.193 dólares por VSA. Em 2005, Papadakis et al. ^23^ publicaram a primeira revisão sistemática de estudos sobre custo-efetividade da RCV como prevenção secundária em pacientes portadores de DAC e IC. ^23^ Em artigo de 2018, ^54^ a avaliação de estudos publicados após 2001 mostrou uma relação de custo-efetividade muito semelhante à descrita anteriormente, sendo o aumento dos gastos com a adição da RCV ao tratamento convencional situado entre 2.555 e 23.598 dólares por VSA.

Cabe ainda destacar que, apesar de mais de 75% das mortes por DCV ocorrerem em países de média e baixa renda *per capita* , ^55^ há uma escassez de dados sobre custo-efetividade da RCV nesses países. ^56^ A maioria das informações é oriunda de nações de alta renda *per capita* , como Estados Unidos, Canadá e países europeus, dificultando a extrapolação dos resultados para a realidade brasileira. Entretanto, vale ressaltar que os poucos estudos disponíveis nos países de média e baixa renda mostram a mesma tendência. No Brasil, a incorporação da reabilitação ao tratamento convencional de pacientes com IC resultou em um aumento de gasto de 21.169 dólares por VSA. ^57^

No entanto, apesar dos claros benefícios clínicos e econômicos da RCV, o percentual de pacientes elegíveis que efetivamente participam desse tipo de serviço está muito aquém do desejado. Segundo dados internacionais, apenas em torno de 30% frequentam um programa de RCV e, no Brasil, estima-se que a situação seja ainda pior, estando certamente muito abaixo de 15%, ^26 , 58 , 59^ pois na maioria dos estados, inclusive na maior parte das capitais e grandes cidades brasileiras, não existe sequer um único serviço de RCV.

Neste contexto, a utilização de modelos de RCVD tem crescido. Inicialmente, a preocupação quanto à segurança da prática do exercício físico fez com que a RCVD fosse destinada somente a pacientes de baixo risco. No entanto, com a demonstração de que não há inferioridade quanto à segurança e com benefícios clínicos semelhantes em relação à estratégia convencional, ^60 - 62^ além do avanço tecnológico de dispositivos que permitem o monitoramento à distância, tem sido ampliada a utilização deste tipo de serviço para o atendimento de pacientes com perfil de risco mais elevado.

Estudos recentes mostram que a RCVD apresenta efetividade semelhante à tradicional, conforme demonstraram Ades et al., ^60^ que compararam os efeitos dos dois modelos em pacientes com DAC de risco leve e moderado em intervenções realizadas por 3 meses, após evento coronariano agudo. Apesar de o grupo de pacientes que atendeu ao programa tradicional ter realizado um volume maior de exercícios físicos, não houve diferença quanto ao ganho em capacidade funcional ou em qualidade de vida entre os dois grupos. Jolly et al. ^62^ compararam os desfechos relacionados aos fatores de risco cardiovasculares entre os programas tradicional e domiciliar por um período mais longo, com seguimentos de 6, 12 e 24 meses, e não observaram diferenças nos resultados.

Recentemente, uma revisão sistemática de estudos com pacientes após IAM, CRVM ou IC, realizada por Anderson et al., ^61^ também não encontrou diferenças significativas entre as duas propostas em relação aos desfechos morte, eventos cardíacos, capacidade funcional, qualidade de vida e fatores de risco modificáveis, no curto prazo (3 a 12 meses) e no longo prazo (até 24 meses).

Assim, programas de RCVD devem ser considerados como estratégia para facilitar o acesso, a adesão e a consequente disseminação da intervenção. Entretanto, existem apenas poucos estudos demonstrando que a RCVD apresenta um custo semelhante ao dos programas tradicionais, ^61 , 63 , 64^ havendo uma grande lacuna de pesquisas que possibilitem a comparação das suas propostas em termos de custo-efetividade. ^65 - 67^

Diante dos fatos, é insustentável que países de todos os níveis de renda e, mais preocupantemente os de média e baixa, continuem fornecendo massivamente e sem critérios de indicação mais rigorosos, intervenções terapêuticas de alto custo e persistam negligenciando em relação a uma estratégia altamente efetiva, economicamente viável e de grande aplicabilidade como a RCV. Portanto, há necessidade da implementação de políticas de saúde pública, com o objetivo de aumentar a disponibilidade, a participação e a adesão dos pacientes elegíveis aos programas de RCV tradicionais e domiciliares.

Por fim, considerando a relevância da RCV, fundamentada em seu amplo benefício clínico e custo-efetividade, impõe-se a adoção de estratégias que modifiquem a cultura médica e favoreçam a disseminação de programas estruturados. Nesse contexto, torna-se relevante que serviços de referência em cardiologia ofereçam a RCV aos seus pacientes durante a internação e após a alta hospitalar. A disponibilidade de um serviço de RCV deveria inclusive ser considerada como um pré-requisito obrigatório para que uma instituição médica fosse reconhecida ou acreditada como de excelência em cardiologia.

## 5. Reabilitação Cardiovascular Domiciliar

O acesso e a adesão dos pacientes a um programa presencial de RCV apresenta diversas barreiras, ^24 - 27 , 68^ que, aliadas a um reduzido encaminhamento médico e uma baixa disponibilidade de serviços, conduzem a uma participação efetiva muito reduzida dos pacientes em programas de exercícios físicos supervisionados. Nesse contexto, programas de supervisão indireta, realizados no ambiente domiciliar (RCVD), surgem como alternativa ou complementação aos programas tradicionais e presenciais de RCV. Em virtude da sua maior abrangência, a RCVD pode ser considerada o principal modo de intervenção quando se trata de estratégia de saúde pública, visando à massificação da RCV na população.

Uma revisão sistemática da Cochrane ^61^ incluiu 23 estudos com 2.890 pacientes cardiopatas (pós-infarto, pós-revascularização, com angina ou IC) e foram comparados os efeitos das RCV convencional e domiciliar. Não foram encontradas diferenças em mortalidade, capacidade física e qualidade de vida. Portanto, a escolha da participação em programas formais ou domiciliares depende da disponibilidade de serviços e das preferências individuais dos pacientes.

Entende-se como RCVD a prática de exercícios físicos sem supervisão presencial, mas orientada e acompanhada pelos profissionais do serviço de RCV. Por essa razão, também é chamada de reabilitação semi-supervisionada, com supervisão indireta ou à distância. As indicações e os objetivos da RCVD são os mesmos do modelo convencional, exigindo os mesmos cuidados em relação à avaliação pré-participação e à prescrição de exercícios. A maioria das sessões é realizada sob supervisão indireta, mas a participação em algumas sessões presenciais, especialmente no início do programa, é de fundamental importância para consolidar o aprendizado das orientações sobre a prescrição e esclarecer dúvidas. Os exercícios podem ser realizados no próprio domicílio ou em parques, vias públicas, ginásios, academias, entre outros, com automonitoramento pelos pacientes, seguindo as orientações recebidas.

Sendo assim, para se obter uma adequada RCVD como estratégia populacional, primeiramente é necessário ampliar a disponibilidade e capacidade de atendimento de programas presenciais de RCV, a fim de possibilitar avaliação inicial, orientações, prescrição dos exercícios físicos e seguimento das sessões domiciliares, com ajustes periódicos por reavaliações. A estratégia domiciliar deve estar alinhada com a da RCV convencional, pois as duas modalidades são paralelas, com pacientes de diferentes perfis de risco, ou sequenciais, com o mesmo paciente em dois momentos clínicos diferentes.

Portanto, assim como a RCV convencional, a primeira etapa da RCVD é o encaminhamento pelo médico assistente, seguido da avaliação pelo médico da reabilitação e demais profissionais, idealmente com realização do teste de esforço (TCPE ou TE) e/ou outras avaliações de aptidão física. Após a avaliação pré-participação, os pacientes definidos como de alto risco podem ser priorizados para a RCV presencial. Já aqueles de menor risco, capazes de automonitoramento e conforme preferências individuais, podem ser direcionados à RCVD. Após receberem as instruções sobre a prescrição dos exercícios, os pacientes executam as sessões por conta própria, podendo haver documentação dos exercícios em planilhas impressas ou eletrônicas, com utilização de recursos como cardiofrequencímetros, pedômetros ou medidores de velocidade e distância percorrida por GPS. Aplicativos de *smartphones* podem intermediar a troca de informações entre os pacientes e a equipe assistencial.

Em alguns casos, um programa de RCV combinado, com sessões presenciais e domiciliares, pode ser a opção para pacientes de risco moderado, em aprendizagem sobre o automonitoramento ou com dificuldade de comparecer às sessões presenciais por problemas sociais ou de deslocamento. A proporção dessa combinação pode variar de acordo com as características clínicas do paciente e a estrutura logística do serviço.

Portanto, o foco é tornar os pacientes fisicamente mais ativos, sendo imperativa a redução do sedentarismo e suas nefastas consequências. Para tal, é fundamental a utilização isolada ou combinada dos recursos disponíveis, seja a atividade física informal, a reabilitação domiciliar ou a convencional.

## 6. Reabilitação Cardiovascular Integrando o Tratamento Clínico Pleno das Doenças Cardiovasculares

A RCV deve estar integrada ao tratamento clínico pleno das DCV, que consiste na ação sinérgica das mudanças estruturadas de estilo de vida com o tratamento farmacológico otimizado, com intervenções indicadas quando existe instabilização clínica e/ou refratariedade ao tratamento clínico inicial. Nos pacientes com DAC estável, até mesmo com isquemia moderada ou grave, a adição de tratamentos intervencionistas não têm se mostrado superiores na redução de desfechos maiores (morte cardiovascular, morte por todas as causas, IAM, IC). ^69 , 70^

Para aumentar a eficácia e a segurança da RCV, é importante que o tratamento farmacológico da DCV esteja adequadamente ajustado, visando aumentar a tolerância ao esforço, o que favorece a execução dos exercícios físicos, reduzindo o risco de eventos. ^3 , 5 , 71 - 73^ Neste contexto, podem ser necessários ajustes de doses e/ou adição de fármacos previamente ao início do programa de exercícios físicos. Por outro lado, após o início da RCV e adequada adesão aos exercícios, alguns pacientes podem requerer retirada ou reduções de doses de fármacos, em virtude da assimilação ao treinamento físico, como, por exemplo, em casos de hipotensão sintomática, bradicardia acentuada e hipoglicemia. ^74 , 75^

### 6.1. Recomendações Gerais para Incremento da Atividade Física e Prática de Exercícios Físicos

Existe associação entre o tempo de sedentarismo, com atividades como assistir à televisão, e maior mortalidade por todas as causas, bem como mortalidade cardiovascular. ^76^ Por isso, para a promoção da saúde e prevenção de DCV, as diretrizes médicas têm recomendado, no mínimo, a prática de exercício físico de intensidade moderada por, pelo menos, 150 minutos semanais ou de alta intensidade por 75 minutos semanais (recomendação 1 B). ^77 - 83^ A prática de mais de 300 minutos semanais de exercício de intensidade moderada a alta pode conferir benefício adicional, conforme já foi evidenciado em pacientes com DAC. ^84^

De acordo com a avaliação individual, a prescrição dos exercícios físicos pode variar em relação às suas diversas características, como tipo (aeróbico, resistência muscular, flexibilidade), modalidade (caminhada, corrida, bicicleta, dança) e duração (tempo de execução), devendo se considerar a frequência semanal e a intensidade ( Tabelas 2 e 3 ).

**Tabela 2:** – Classificações do exercício físico

Denominação		Característica
Pela via metabólica predominante	Anaeróbico alático	Grande intensidade e curtíssima duração
	Anaeróbico lático	Grande intensidade e curta duração
	Aeróbico	Baixa ou média intensidade e longa duração
Pelo ritmo	Fixo, constante ou contínuo	Sem alternância de ritmo ao longo do tempo
	Variável, intermitente ou intervalado	Com alternância de ritmo ao longo do tempo
Pela intensidade relativa*	Baixa ou leve	Respiração tranquila, muito pouco ofegante (Borg < 4)
	Média ou moderada	Respiração acelerada, ofegante, mas controlada. Consegue falar uma frase (Borg 4 a 7)
	Alta ou pesada	Respiração muito acelerada, muito ofegante. Fala dificultada (Borg > 7)
Pela mecânica muscular	Estático	Não ocorre movimento, apenas tensão/recrutamento muscular.
	Dinâmico	Ocorre movimento com a contração muscular realizada.

* Para a classificação, considerou-se a versão da escala de Borg, que varia entre 0 e 10.

**Tabela 3:** – Métodos de prescrição de intensidade moderada para os exercícios físicos aeróbicos

Método	Descrição
Sensação subjetiva de esforço (Borg)	Exercícios com a autopercepção de esforço como moderado, médio ou pesado, situando-se entre 2 e 4 na escala de Borg 0-10 ou 10 a 13 na escala 6-20
Teste da fala	Execução dos exercícios em intensidade em que a respiração seja ofegante, porém controlada, de modo que se consiga completar uma frase sem pausas
Percentuais da FC pico	Exercícios na intensidade entre 70 e 85% da FC pico* FC alvo = FC pico x percentual
FC de reserva (Karvonen)	Exercícios na intensidade entre 50 a 80% da FC de reserva (FC pico – FC repouso)* FC alvo = FC repouso + (FC pico – FC de repouso) x percentual
Limiares no teste cardiopulmonar	Execução dos exercícios em intensidade entre os limiares ventilatórios 1 e 2 (limiar anaeróbico e ponto de compensação respiratória)

FC: frequência cardíaca. * É preferencial a utilização da FC pico obtida em um teste de esforço máximo, visto que existem variações individuais que causam erros na predição da FC por idade, especialmente em pacientes em uso de medicações com efeito cronotrópico negativo.

Pacientes sedentários devem iniciar os exercícios no limite inferior da prescrição, progredindo gradativamente ao longo das semanas seguintes. A progressão inicial pode ser feita na duração da sessão e, posteriormente, na intensidade dos exercícios. Pacientes já fisicamente ativos podem realizar, desde o início, exercícios em níveis mais intensos, objetivando um mínimo de 75 minutos, divididos em duas ou mais sessões semanais.

Os exercícios de resistência muscular localizada, seja de fortalecimento ou de potência, têm se mostrado bastante benéficos para a saúde geral e para os sistemas cardiovascular e osteomuscular, sendo de fundamental importância nos pacientes com sarcopenia e/ou osteopenia. Devem ser realizados, pelo menos, duas vezes por semana, privilegiando grandes grupos musculares de MMSS, MMII e tronco. Podem ser feitos utilizando o próprio peso corporal do indivíduo ou usando implementos como pesos livres, caneleiras, faixas elásticas, aparelhos de musculação, entre outros recursos. A carga ou peso, para cada exercício ou movimento, deve ser individualmente ajustada, além de se ter a devida atenção à execução dos movimentos para que a técnica e a postura sejam corretas.

Existem diferentes protocolos para exercícios resistidos, com variações no número de exercícios utilizados por sessão, como, por exemplo, de 6 a 15; na quantidade de séries para cada exercício, em geral de 1 a 3; e no número de repetições, que pode oscilar entre 6 e 20. A intensidade dos exercícios resistidos pode ser ajustada de acordo com a intensidade relativa da força máxima e pode ser expressa em função da carga máxima possível para realizar uma repetição máxima (Teste de 1 repetição máxima ou 1RM). Carga de intensidade leve seria até 30% de 1RM; intensidade média, entre 30 e 60 ou 70% de 1RM; e intensidade alta, acima de 60 ou 70% de 1RM. Outra possibilidade é a prescrição dos exercícios físicos resistidos de modo subjetivo, pela sensação de esforço (ver Tabela 2 ).

Um modo prático é o método de repetição variável, que tem como objetivo executar uma faixa de repetições (p. ex., de 10 a 15 repetições). Se o paciente não conseguir realizar corretamente o movimento na repetição mínima prescrita, significa que a carga aplicada está elevada. Por outro lado, se conseguir a repetição máxima prescrita de modo fácil, é porque a carga está leve. Assim, a carga será ajustada para que o treinamento ocorra dentro da faixa de repetições proposta. Esse método pode ser aplicado aos mais variados exercícios localizados e pode ser utilizado na progressão da prescrição, sendo que os limites da repetição podem ser modificados, dependendo dos objetivos almejados (força, hipertrofia ou resistência muscular).

Os exercícios de flexibilidade podem oferecer benefícios osteomioarticulares, na qualidade de vida relacionada à saúde e na prevenção de queda em idosos. Ao contribuírem para uma movimentação articular mais fácil e eficiente, reduzem a demanda por oxigênio em situações de movimento, favorecendo o desempenho do sistema cardiovascular. Nesses exercícios, procura-se alcançar a amplitude máxima do movimento, chegando até o ponto de leve desconforto, devendo a posição ser mantida estaticamente por 10 a 30 segundos.

Dependendo da faixa etária, das condições clínicas e dos objetivos do programa de exercício para um dado paciente, outros tipos de exercício podem ser incluídos na prescrição, como os de coordenação motora e de equilíbrio. Além disso, devem ser considerados os inúmeros benefícios decorrentes de formas mais lúdicas e socializantes de exercícios, como a dança e outras modalidades. ^85 , 86^

A avaliação da aptidão física aeróbica e não aeróbica possibilita uma prescrição mais individualizada dos exercícios físicos, com o objetivo de se obterem os melhores resultados e, por meio da estratificação de risco e da busca de eventuais anormalidades, minimizar os riscos da prática. De modo geral, a avaliação inicial tem como base a anamnese, o exame físico e o ECG. Avaliações mais detalhadas deverão ser individualizadas, com realização de TCPE ou TE, avaliação antropométrica, de força/potência muscular e de flexibilidade. Na avaliação inicial, pode-se quantificar o déficit funcional frente ao desejável, bem como estabelecer metas a serem alcançadas. É importante enfatizar que os pacientes com baixa aptidão física inicial são os que mais se beneficiam da RCV, após adequada aderência ao programa de exercício supervisionado. ^87^ É também possível obter subsídios clínicos e funcionais que possibilitem um adequado aconselhamento da atividade sexual, com base no modelo do KiTOMI, que foi proposto por autores brasileiros em 2016. ^88^ Além disso, é fundamental para o paciente a reavaliação, com o intuito de estimular o comprometimento e mensurar a evolução e os benefícios obtidos.

Finalizando, vale ressaltar a fundamental importância do estabelecimento de um sistemático esquema de reavaliações, que, além de estimular o comprometimento dos pacientes, torne possível mensurar a evolução e os benefícios obtidos, produzindo relatórios que estimulem os ajustes do tratamento e que, portanto, devem ser sempre encaminhados aos médicos assistentes, os quais obviamente devem integrar ativamente o tratamento clínico pleno.

### 6.2. Hipertensão Arterial Sistêmica

A hipertensão arterial sistêmica (HAS) permanece como um dos maiores fatores de risco para o desenvolvimento de DAC, IC, DRC e acidente vascular cerebral (AVC) isquêmico ou hemorrágico, representando, social e economicamente, um enorme desafio à saúde pública mundial. ^89^ Houve um aumento global do número de hipertensos de 594 milhões em 1975 para 1,13 bilhão em 2015, em grande parte creditado aos países subdesenvolvidos e em desenvolvimento. ^90^ Considerando que a maioria dos casos está relacionada ao estilo de vida, com o sedentarismo ocupando lugar de destaque, fica clara a importância dos exercícios físicos ao lado de outras medidas comportamentais, além do uso de medicações, sempre que indicadas. ^72^

#### 6.2.1. Benefícios Terapêuticos dos Exercícios Físicos

A HAS apresenta fisiopatologia complexa e multifatorial, com modificações estruturais e fisiológicas, em particular, nos sistemas vascular (rarefação capilar, aumento da rigidez arterial e da razão parede/diâmetro das arteríolas), renal (diminuição da filtração glomerular, aumento da renina plasmática e da reabsorção de sódio e água) e neural (aumento da atividade simpática e de quimiorreceptores, diminuição da atividade parassimpática e da sensibilidade barorreflexa). ^91^ A prática regular de exercícios físicos exerce efeito terapêutico na reestruturação fisiológica desses sistemas, com redução do estresse oxidativo e da inflamação, correção da disfunção barorreflexa, aumento do tônus vagal, diminuição da atividade simpática, reversão do remodelamento hipertrófico arteriolar em tecidos exercitados e redução da resistência vascular periférica, com consequente diminuição da PA e controle dos níveis pressóricos semelhante, ou mesmo superior, ao proporcionado pela farmacoterapia. ^92 , 93^

No tecido vascular, a HAS caracteriza-se por desorganização das células musculares lisas, aumento dos depósitos de colágeno e diminuição da razão elastina/colágeno, além da formação de fibra elástica anormal e lâmina elástica interna com menor área fenestrada. ^94^ Todas essas alterações estruturais da parede do vaso, que ocorrem tanto em território arterial como arteriolar, elevam a rigidez do sistema vascular, com consequente aumento da velocidade da onda de pulso do fluxo sanguíneo, da pressão de pulso (diferença entre a PA sistólica [PAS] e a diastólica [PAD]) e da pressão hidrostática no capilar. Soma-se a todo esse desequilíbrio estrutural a disfunção do endotélio, com o aumento de substâncias vasoconstritoras, de mediadores inflamatórios e de agentes oxidantes, em detrimento da produção de agentes vasodilatadores e antioxidantes. ^95 , 96^

O exercício físico, por meio do aumento do estresse tangencial derivado da fricção do fluxo sanguíneo na superfície endotelial da parede do vaso (definido como força de cisalhamento e comumente descrito pelo termo “ *shear stress* ”), estimula positivamente o tecido endotelial, com aumento da produção de enzimas antioxidantes e agentes vasodilatadores, além de diminuição da ação dos radicais livres, das citocinas pró-inflamatórias, das moléculas de adesão e dos agentes vasoconstritores, restaurando, assim, o equilíbrio do funcionamento endotelial. ^97 , 98^ Estudos experimentais ^94^ em ratos espontaneamente hipertensos demonstram a reorganização de todas as estruturas vasculares da artéria aorta após a implementação de um período de exercício aeróbico. O treinamento aeróbico promove adaptações vasculares nas artérias de condutância (com diminuição da rigidez arterial e melhora da função endotelial), nas arteríolas (pela diminuição da razão parede/luz do vaso) e nos capilares, estimulando a angiogênese. ^99 , 100^

Dessa maneira, a prática de exercícios físicos atua de modo multifatorial na HAS, sendo considerada intervenção-chave para mitigar o ônus da doença e suas comorbidades, ^101^ com efeito anti-hipertensivo semelhante ao das medicações, ^102^ embora essa ação possa se superpor às dos fármacos e, eventualmente, exigir ajustes das doses medicamentosas.

Os exercícios físicos aeróbicos são os mais bem estudados e com maiores evidências de benefícios na redução pressórica em hipertensos, algo corroborado na meta-análise de Cornelissen et al., que mostrou redução média da PAS de 8,3 mmHg e da diastólica de 5,2 mmHg, decorrente dos exercícios aeróbicos.

Os exercícios de resistência, que também exercem efeito anti-hipertensivo, ^103^ agem na preservação ou no aumento de massa muscular, força e potência, fatores que diminuem a intensidade relativa para realização de tarefas do cotidiano, com consequente amortecimento da resposta pressórica, além de possivelmente promoverem uma melhora da sensibilidade barorreflexa. ^104^

Além dos exercícios aeróbicos e resistidos dinâmicos, existem alguns estudos sobre exercícios isométricos (resistidos estáticos), que têm demonstrado efeitos expressivos na redução dos níveis tensionais. ^105 - 107^ Em uma meta-análise, foi demonstrado que o exercício isométrico de *handgrip* , realizado por 12 minutos, 3 a 5 vezes por semana, reduziu a PA em 5,2/3,9 mmHg. ^108^ No entanto, há falta de estudos sobre a segurança e a eficácia dessas modalidades em longo prazo.

#### 6.2.2. Indicações de Exercícios Físicos na Hipertensão Arterial Sistêmica

Maiores níveis de atividade física têm sido associados a uma diminuição no risco de desenvolvimento de HAS. Com o advento dos rastreadores eletrônicos de atividade e do monitoramento ambulatorial da PA, tornou-se cada vez mais viável a realização de estudos que correlacionem a atividade física e a PA. ^109^ A aptidão física, medida objetivamente por meio de testes de esforço graduados, atenua o aumento da pressão com a idade e impede o desenvolvimento de hipertensão. Em uma coorte de homens de 20 a 90 anos de idade, que foram seguidos por 3 a 28 anos, uma maior aptidão física diminuiu a taxa de aumento pressórico ao longo do tempo e atrasou o período até o início da HAS. ^110^ Estudos epidemiológicos têm revelado associação inversa entre o nível de atividade física e a aptidão cardiorrespiratória, com a presença de hipertensão arterial. ^111 , 112^

Os grandes ensaios clínicos randomizados e as meta-análises têm confirmado que o exercício físico regular pode reduzir os níveis pressóricos. ^102 , 112^ Além disso, a prática constante de atividades físicas pode ser benéfica tanto na prevenção quanto no tratamento da hipertensão, reduzindo a morbimortalidade cardiovascular. Indivíduos ativos apresentam um risco até 30% menor de desenvolver hipertensão que os sedentários ^111^ e o aumento da atividade física diária reduz a pressão aterial de maneira significativa. ^113^

A inatividade física tem sido um dos maiores problemas de saúde pública do mundo moderno, ^114^ por ser o mais prevalente dos fatores de risco cardiovasculares e um dos principais fatores contribuintes para mortalidade no mundo. ^115^ A sobrevida é menor em pessoas que passam a maior parte do tempo sentadas do que naquelas que passam pouco tempo desse modo. ^116^ Há relação direta entre o período sentado ou o tempo de televisão com níveis elevados de PA, morbidade e mortalidade cardiovascular. ^117^ Por esta razão, para a redução do tempo sentado, recomenda-se levantar-se por, pelo menos, 5 minutos a cada 30 minutos sentado, como medida válida de prevenção. A prática de exercícios físicos está indicada para todos os pacientes com HAS ( Tabela 4 ). ^72 , 73 , 118^

**Tabela 4:** – Indicação de exercícios físicos na hipertensão arterial sistêmica

Indicação	Recomendação	Nível de evidência
Exercícios físicos aeróbicos na prevenção do desenvolvimento de HAS ^110-112^	I	A
Exercícios físicos aeróbicos no tratamento da HAS ^93,102,103,112^	I	A
Exercícios físicos de resistência muscular dinâmicos no tratamento da HAS ^103,112^	I	B
Exercícios físicos isométricos no tratamento da HAS ^105-108^	IIa	B

*HAS: hipertensão arterial sistêmica.*

Além dos exercícios, o tratamento da hipertensão requer outras modificações do estilo de vida, como alimentação correta, controle do peso e remoção de fatores de risco como o tabagismo e o excesso de consumo alcoólico.

Além do efeito direto dos exercícios na HAS, outro componente importante da RCV se relaciona ao manejo da terapia farmacológica, a qual pode ser otimizada no ambiente da reabilitação, por meio de educação sobre a doença, aconselhamento quanto à necessidade de tratamento e informações sobre os efeitos colaterais e importância da adesão. ^119^

#### 6.2.3. Avaliação Pré-participação

Obviamente, cabe ao médico assistente estabelecer o diagnóstico da HAS, pesquisar outros fatores de risco cardiovasculares e rastrear lesões em órgãos-alvo e outras doenças associadas, de modo a definir a estratégia terapêutica, que pode ser farmacológica e/ou composta de uma ou mais modificações comportamentais. ^72^

Na avaliação para a prescrição de exercícios físicos é relevante a realização de um TCPE ou TE, especialmente se houver suspeita de cardiopatia, lesões em órgão-alvo ou presença de três ou mais fatores de risco. ^72^ Quando o TCPE ou TE for utilizado para prescrição de exercícios físicos, o ideal é que seja executado na vigência das medicações habituais, principalmente das que inibam a resposta cronotrópica, a fim de mimetizar a condição que estará presente durante as sessões de treinamento físico, possibilitando a utilização da FC pico do TE ou os limiares ventilatórios do TCPE para a determinação da zona-alvo de treinamento com base na FC.

#### 6.2.4. Particularidades na Prescrição e no Acompanhamento dos Exercícios Físicos

A recomendação de exercício para pacientes hipertensos, de maneira semelhante ao proposto para a população em geral, é de, pelo menos, 150 minutos por semana (5 sessões de 30 minutos) de atividade aeróbia de moderada a alta intensidade, sendo aconselhável associar duas a três sessões de exercícios resistidos por semana. Na ausência de contraindicações, pode ocorrer aumento gradativo, visando a meta de 300 min/semana de exercícios aeróbios de intensidade moderada ou 150 min/semana de exercícios aeróbios de alta intensidade, para obtenção de maiores benefícios.

Durante o treinamento, é importante que a PA seja avaliada em repouso e em esforço. Para pacientes com valores em repouso superiores a 160/100 mmHg ou com lesão de órgãos-alvo (hipertrofia ventricular esquerda, retinopatia, nefropatia e outras), é recomendado o ajuste dos fármacos anti-hipertensivos para melhor controle pressórico antes de iniciar ou retornar às sessões de exercício, ^37^ ou a redução da intensidade de treinamento até a obtenção de melhor controle pressórico. Em programas de RCV supervisionados, flexibilizações dessas recomendações podem ser realizadas individualmente, de acordo com a avaliação do médico da reabilitação e as respostas observadas no teste de esforço e nas sessões de exercícios. Durante o exercício, é recomendado que a PA se mantenha inferior a 220/105 mmHg. Se estiver superior a esse nível, deve-se considerar a interrupção da sessão ou a redução da intensidade de cargas, considerando o ajuste das medicações. ^37^

Após a sessão de exercício, a PA deve ser verificada e é comum a identificação de valor inferior ao observado antes do início das atividades. Em hipertensos, o efeito anti-hipertensivo agudo de uma sessão tende a ser maior com níveis mais intensos de exercícios aeróbios. ^120^ Esse efeito agudo do treinamento físico pode causar hipotensão sintomática após o término, que geralmente melhora com repouso e hidratação. Pacientes em uso de alfabloqueadores, betabloqueadores, bloqueadores de canais de cálcio e vasodilatadores podem ter maior risco de hipotensão pós-exercício, necessitando de atenção especial no desaquecimento. A recorrência dessa situação, que costuma decorrer do resultado da assimilação do treinamento que se soma aos efeitos anti-hipertensivos dos fármacos, exige considerar a necessidade de ajustes das doses ou mesmo suspensão de medicamentos.

Há poucos dados quanto ao efeito do exercício em pacientes com hipertensão resistente, que se caracteriza pela PA acima da meta apesar do uso de três ou mais medicações anti-hipertensivas. Em relação a esses pacientes, que requerem maior monitoramento, um ensaio clínico randomizado unicêntrico mostrou que exercício em água aquecida (30 a 32ºC) resultou em pronunciada redução da PA (36/12 mmHg) após 3 meses. ^121^ Embora tais efeitos precisem ser reproduzidos em mais estudos, exercício em água aquecida parece ser apropriado para pacientes com hipertensão arterial resistente.

## 6.3. Coronariopatia Estável após Evento Agudo ou Revascularizações

As DCV, lideradas pela DAC, são responsáveis pela maior parte das mortes da população adulta. ^122 - 124^ Os mecanismos subjacentes da DAC estável incluem obstrução aterosclerótica dos vasos epicárdicos, doença microvascular e espasmo coronário, isolados ou em associação. ^5^ Clinicamente a manifestação mais comum da DAC estável é a angina do peito, que se caracteriza por episódios reversíveis de dor torácica por isquemia miocárdica, decorrentes do desequilíbrio entre oferta e consumo de oxigênio pelo miocárdio, em geral desencadeados pelo esforço físico ou estresse emocional, que cessam com o repouso ou uso de nitrato de ação rápida. ^5^

A DAC estável tem bom prognóstico, com mortalidade anual estimada em 1,5% e incidência de infarto não fatal de 1,4%, ^125^ sendo fundamental o tratamento clínico pleno, com otimização do tratamento farmacológico e prática de exercícios físicos regulares, além de outras modificações comportamentais relacionadas a tabagismo, dieta e composição corporal. Revascularizações eletivas também podem ser indicadas nos pacientes com DAC estável, a depender da sintomatologia e do risco cardiovascular. ^5^ Porém, vale ressaltar que, quando o quadro é estável, mesmo nos pacientes com angina, o tratamento exclusivamente clínico não tem se mostrado inferior ao tratamento com adição de abordagem intervencionista. ^70 , 126 , 127^ A ocorrência de eventos agudos de instabilização da doença, com quadros de IAM ou angina instável, está relacionada com elevado aumento do risco cardiovascular, frequentemente exigindo ajustes da terapia farmacológica e revascularização cirúrgica ou percutânea de urgência. ^128 - 131^

### 6.3.1. Benefícios Terapêuticos dos Exercícios Físicos

Estão cientificamente demonstrados os efeitos benéficos do exercício físico regular realizado em curto e longo prazos nos portadores de DAC estável. Em um período inicial de reabilitação cardiovascular, de 8 a 12 semanas, destacam-se o aumento do limiar isquêmico, ^132 - 136^ a melhora da capacidade funcional cardiorrespiratória ^132 , 134 , 136^ e a melhora perfusional cintilográfica. ^137 - 140^ Os benefícios adquiridos persistem com a manutenção da prática regular de exercícios físicos, ^103 , 141 - 144^ que contribui para a melhora da qualidade de vida ^1 , 146^ e redução das taxas de mortalidade cardiovascular e hospitalização. ^1 , 144 , 146 - 148^

Em pacientes com DAC estável, diferentes mecanismos explicam o aumento do limiar isquêmico, permitindo gradativamente cargas superiores de atividade física. A redução do duplo produto para cargas submáximas de trabalho está associada, dentre outros mecanismos, à melhora da modulação autonômica cardíaca. ^144^ Destaca-se ainda um aumento da perfusão miocárdica decorrente da melhora da resposta vasodilatadora dependente do endotélio ^149 - 151^ e do aumento do recrutamento de vasos colaterais durante o exercício, ^134 , 144 , 152^ algo que reflete na atenuação da depressão do segmento ST durante o exercício. ^35 , 132 , 137^ Ressalta-se, ainda, que o treinamento físico associado à dieta pobre em gorduras pode influenciar na progressão da placa aterosclerótica. ^152 , 153^

A RCV é uma terapia adjuvante eficaz no tratamento de pacientes após evento coronariano agudo, CRVM e ICP. Revisão sistemática e meta-análise ^1^ de 63 estudos envolvendo 14.486 pacientes com idade entre 47 e 71 anos revelaram que a RCV reduziu a mortalidade cardiovascular em 26% e a hospitalização global em 18%, com melhora adicional na qualidade de vida nessa população, devendo ser encorajada sempre que possível.

A melhora da capacidade cardiorrespiratória é um dos fatores responsáveis pelos achados na redução da mortalidade total. Em coorte realizada com 5.641 pacientes participantes de RCV no Canadá, verificou-se que cada 1 MET de aumento na capacidade cardiorrespiratória durante a RCV reduziu a mortalidade total em 25%. ^154^ Outros estudos similares reportaram redução da mortalidade cardíaca ou total entre 8 e 34% para cada MET de melhora na capacidade cardiorrespiratória. ^155 , 156^

Além disso, a RCV oferece efeito adicional na redução de eventos cardiovasculares após ICP, conforme evidenciado pelo estudo ETICA ( *Exercise Training Intervention After Coronary Angioplasty* ), em que houve aumento de 26% no VO _2_ pico, melhora de 27% na qualidade de vida e redução de 20% nos eventos cardíacos, incluindo diminuição de IAM e menor número de hospitalizações em pacientes que realizaram RCV após angioplastia, quando comparados aos que permaneceram sedentários. ^157^

### 6.3.2. Quando Indicar Reabilitação

A RCV está indicada em todos os casos de DAC ( Tabela 5 ), sendo considerada útil e efetiva, tanto quando é centrada somente no exercício físico como quando é acompanhada de conteúdo educacional, manejo de fatores de risco e aconselhamento psicológico. ^146^

**Tabela 5:** – Indicação de reabilitação cardiovascular na doença arterial coronariana

Indicação	Recomendação	Nível de evidência
RCV para redução da isquemia miocárdica ^132-140,158^	I	A
RCV para aumento da capacidade física ^132,134,140^	I	A
RCV para redução de mortalidade ^1,154,155^	I	A
RCV após evento coronariano ou revascularização ^140,157^	I	A
RCV precoce (uma semana após evento agudo) ^159,160^	IIa	A
RCV em pacientes com angina refratária ^161,162^	IIb	C

*RVC: reabilitação cardiovascular.*

Apesar de tratamentos intervencionistas cada vez mais precoces e diminuição no tempo de permanência hospitalar após síndrome coronariana aguda, não é incomum o paciente iniciar a reabilitação apenas após seu retorno ambulatorial com médico assistente, o que pode significar 15 dias ou mais após o evento. O início precoce é possível e pode interferir direta e positivamente na aderência e nos benefícios clínicos alcançados após o evento agudo.

Uma das maiores preocupações do treinamento físico precoce refere-se ao seu efeito no processo de remodelamento ventricular. Enquanto alguns autores reportam efeitos negativos, ^163^ outros relatam efeitos positivos ^158 , 164^ ou mesmo neutros ^139^ sobre esse processo. Uma revisão sistemática e meta-análise ^159^ realizada para responder a essa questão identificou que as mudanças observadas na função e no diâmetro ventriculares, bem como a capacidade funcional, foram diretamente relacionadas ao tempo de início do treinamento. As maiores mudanças no remodelamento ventricular e na capacidade funcional foram obtidas quando os programas eram iniciados na fase aguda após o evento (após 6 horas a 7 dias), com diminuição desses efeitos entre 7 e 28 dias e superior a 29 dias, quando progressivamente se perdia o efeito positivo sobre o remodelamento ventricular. É importante ressaltar que não houve diferença em relação a eventos entre as fases de início do treinamento e que a amostra estudada foi prioritariamente de homens jovens, o que reforça a necessidade de mais estudos, principalmente em outras populações, como a de idosos e mulheres. Para cada 1 semana de atraso no início dos exercícios após o infarto, poderá ser necessário 1 mês adicional de treinamento para obtenção de benefícios similares no volume sistólico final e na fração de ejeção do ventrículo esquerdo (FEVE). ^160^

Embora referendada amplamente pela literatura médica por seus efeitos benéficos e custo-efetividade, somente uma minoria dos pacientes elegíveis participa de programas de RCV, algo explicável por múltiplas barreiras, como inexistência de programas, dificuldade de acesso aos serviços, reduzido número de encaminhamentos, mobilidade urbana de má qualidade, entre outros, afetando principalmente mulheres, idosos e minorias étnicas. ^165 - 168^ Sendo assim, mudanças políticas, sociais, estruturais e na cultura médica são necessárias para modificar esse cenário.

### 6.3.3. Avaliação Pré-participação e Prescrição de Exercícios

Tanto nos pacientes com DAC estável como naqueles após evento coronário e/ou revascularizações, é fundamental a estratificação de risco para a RCV, por meio de avaliação clínica focada no conhecimento detalhado da DCV e nos tratamentos realizados, sejam medicamentosos ou intervencionistas. Questões relacionadas com existência de sintomas, função ventricular, capacidade funcional, presença de arritmias e possibilidade de isquemia residual auxiliam na estratificação e devem fazer parte da avaliação inicial. O ideal é que essa avaliação médica seja realizada por profissional integrado à equipe da RCV (médico da reabilitação).

O perfil de um paciente encaminhado à RCV pode ser bastante variado, desde o que é submetido a tratamento de maneira eletiva até aquele com síndrome coronariana aguda complicada e internação prolongada. Uma avaliação mais ampla, incluindo questões nutricionais, psicológicas e osteomusculares, deve fazer parte da anamnese, pois esses fatores podem impactar diretamente no processo da RCV. Sempre devem ser realizadas cuidadosas avaliações, seja do local de punção arterial, principalmente no acesso femoral, nos pacientes submetidos à ICP, assim como das feridas cirúrgicas, em especial quanto à estabilidade esternal e eventuais infecções, nos pacientes submetidos à CRVM. A presença de situação que implique necessidade de cuidados especiais exige as pertinentes orientações à equipe responsável pelo treinamento físico dos pacientes.

A avaliação pré-participação para a RCV, por meio de provas funcionais, objetiva o melhor conhecimento da capacidade funcional, a avaliação de isquemia residual e a pesquisa de arritmias induzidas pelo esforço. A identificação de isquemia miocárdica ao esforço é realizada por meio da ocorrência de sintomas como angina de peito e/ou por alterações eletrocardiográficas. O limiar isquêmico identificado no TE pelo início dessas alterações clínicas e/ou eletrocardiográficas, pode eventualmente ser caracterizado segundo a carga de trabalho e FC, a partir dos quais a isquemia se manifesta, algo que poderia ser utilizado na prescrição de exercício.

O TE, para fins de prescrição, deverá será realizado sob o uso das medicações habituais, principalmente as que causam interferência na FC, para que haja reprodução na avaliação da condição que estará presente durante as sessões de treinamento. Por exemplo, em situações de pacientes que alteram a dose de betabloqueador durante a reabilitação, o ideal seria realizar um novo teste para ajuste da prescrição. Em caso de impossibilidade dessa conduta, o uso da percepção subjetiva de esforço poderá auxiliar na prescrição até a realização de novo exame.

Em alguns casos, os pacientes ingressantes na RCV podem estar com alguma limitação clínica para realização de um teste funcional máximo. Nestes, pode-se realizar um teste inicial submáximo para guiar a prescrição, com posterior teste máximo após a melhora clínica e/ou otimização do tratamento farmacológico. Considerando a possibilidade de grandes erros, devido à intensa variação individual da resposta cronotrópica, não devem ser usadas fórmulas que consideram a idade para definição da FC pico, sendo este erro ainda maior nos pacientes em uso de betabloqueadores.

Quando a reabilitação for iniciada sem a execução de um teste funcional, a prescrição poderá basear-se na escala de percepção subjetiva de esforço (escala BORG entre 11-15, na escala de 6-20) e com limitação da FC de treinamento de modo arbitrário, ou seja, a utilização da FC de repouso + 20 bpm para pacientes que tiveram síndrome coronariana aguda, ou FC de repouso + 30 bpm para aqueles após cirurgia ou tratamento intervencionista eletivo. ^131^ A intensidade também pode ser determinada pela ventilação pulmonar, sendo a atividade considerada de moderada intensidade quando o indivíduo permanece apenas discretamente ofegante, conseguindo falar frases completas sem interrupções (ver Tabela 3 ).

Quando o TE for realizado, a intensidade dos exercícios prescritos poderá situar-se entre 40 e 80% da FC de reserva [método de Karvonen: (FC pico – FC de repouso) x percentual de intensidade + FC de repouso]. Nesses casos, habitualmente se inicia com a FC no limite inferior da prescrição, sendo realizadas progressões, conforme a evolução clínica e melhora da capacidade funcional. A maioria dos pacientes terá intensidade prescrita entre 50 e 70% da FC de reserva. Os mais limitados ou com disfunção ventricular importante poderão trabalhar em intensidades menores, entre 40 e 60%, e aqueles previamente ativos e com melhor capacidade funcional, entre 50 e 80%. Os percentuais da FC pico também podem ser utilizados, sendo que intensidades moderadas correspondem de 70 a 85% da FC pico (ver Tabela 3 ).

O TCPE, por meio da análise da resposta do pulso de oxigênio, contribui para o aumento da sensibilidade e especificidade para o diagnóstico da isquemia miocárdica. ^169^ Na presença de platô precoce do pulso de oxigênio ou, principalmente, queda dessa variável durante o esforço, a prescrição da intensidade do exercício pode ser limitada às cargas abaixo dessa ocorrência. Desse modo, o TCPE é considerado o padrão-ouro na avaliação para a prescrição dos exercícios e deve ser utilizado sempre que estiver disponível. ^169 - 171^ Nesses casos, a recomendação de intensidades moderadas poderá ser realizada entre os limiares ventilatórios (limiar anaeróbico e ponto de compensação respiratória), com evolução da intensidade de maneira progressiva.

Em relação ao volume dos exercícios, recomenda-se, no mínimo, 150 minutos por semana, que poderá ser distribuído em 3 a 5 sessões. De acordo com a tolerância, adaptações ao treinamento e preferências individuais, além das considerações sobre o quadro clínico, este volume poderá aumentar para 300 minutos ou mais por semana.

Em relação ao treinamento resistido, o método considerado padrão-ouro para a prescrição da intensidade seria o teste de uma repetição máxima. Entretanto, na prática, muitos programas de reabilitação não o utilizam pela limitação de tempo para aplicá-lo, ou mesmo por razões clínicas, como em pacientes submetidos a CRVM, que podem ter limitações não apenas pela esternotomia, mas também por lesões da safenectomia. Nesses casos, a percepção subjetiva de esforço é um método prático e útil.

Em pacientes com esternotomia, trabalhos com MMSS devem ter cargas restritas durante 5 a 8 semanas e limitadas a baixas intensidades. Exercícios com amplitude de movimentos com os braços poderão ser permitidos após essa fase, se não existir instabilidade de esterno, embora novos estudos estejam avaliando a segurança de prescrição mais precoce do exercício após a CRVM. ^172 , 173^

Os pacientes devem sempre ser orientados quanto à maneira correta de execução do movimento e à respiração, evitando a manobra de Valsalva. O intervalo entre as séries dos exercícios resistidos pode ser entre 45 segundos e 1 minuto, a depender das cargas aplicadas e da tolerância do paciente.

### 6.3.4. Particularidades na Prescrição e Acompanhamento dos Exercícios Físicos

***6.3.4.1. Angina Refratária***

Pacientes com angina refratária são caracterizados por angina limitante com evolução superior a 3 meses, em tratamento clínico otimizado, com documentação de isquemia miocárdica e não considerados elegíveis para intervenção coronariana percutânea e/ou cirúrgica. ^174 , 175^ Tais pacientes geralmente não são referenciados aos programas de RCV, devido ao temor de eventos adversos durante o treinamento físico, embora a reabilitação já tenha sido considerada como uma possibilidade terapêutica exequível e segura para esses pacientes. ^175^

O objetivo das intervenções terapêuticas nesse cenário contribui para a melhora da qualidade de vida, facilitando a realização de atividades da vida diária. ^176 - 178^ Há um único estudo controlado envolvendo RCV em pacientes com angina refratária. Ele avaliou 42 indivíduos, randomizados para o programa de exercícios de RCV ou manutenção do tratamento clínico habitual, durante 8 semanas. Os pacientes do grupo de exercício receberam a prescrição de FC de treino entre 60 e 75% da FC de reserva (para aqueles com função ventricular preservada) e entre 40 e 60% da FC de reserva quando a FEVE era inferior a 40%. Os pacientes do grupo de reabilitação aumentaram em 50 m a distância total no teste de caminhada (avaliado pelo *Shuttle Walk test* ), sem mudança na intensidade ou frequência da angina e sem eventos adversos em ambos os grupos. ^161^

Um estudo brasileiro randomizado ainda em andamento ^162^ está avaliando a segurança e eficácia de um programa de exercícios realizado durante um período de 12 semanas, supervisionado em ambiente hospitalar e com monitoramento eletrocardiográfico contínuo. A prescrição está sendo individualizada e pautada nos parâmetros do TCPE e no limiar de isquemia e/ou angina. Até o momento, 42 pacientes foram incluídos, e não houve documentação de eventos cardiovasculares e hospitalizações relacionadas aos exercícios. A troponina T ultrassensível, preditor conhecido de pior prognóstico, ^179^ não apresentou oscilação no seu nível sérico em 32 pacientes submetidos a uma sessão aguda de exercício aeróbico (no limiar de isquemia) de 40 min de duração, no momento da inclusão no estudo ( *dados não publicados* ).

Nos pacientes com angina refratária e baixo limiar isquêmico, a utilização de nitratos de ação rápida antes do início da sessão de treinamento físico pode contribuir para um treino mais duradouro e até maiores intensidades de exercícios. ^180^

***6.3.4.2. Treinamento com Indução de Isquemia Miocárdica***

Tradicionalmente, existe a recomendação de que os exercícios físicos nos pacientes com DAC sejam realizados abaixo do limiar isquêmico clínico e eletrocardiográfico. Porém, isso pode ser difícil de controlar. Estudos prévios já demonstraram que os exercícios físicos, prescritos conforme recomendações da literatura, podem desencadear defeitos de perfusão cintilográficos, que não são evidenciados por meio de anormalidades no ECG e angina, ^181 , 182^ pois as alterações contráteis e os defeitos perfusionais precedem as alterações clínicas e eletrocardiográficas isquêmicas. ^183 , 184^

O significado funcional da indução de isquemia cintilográfica ainda é incerto, mas estudos com realização de treinamentos acima do limiar isquêmico já foram realizados. Em um estudo com realização de uma única sessão de treinamento com duração de 20 min acima do limiar isquêmico, não foram identificados indícios de dano miocárdico agudo. ^185^ Outros autores demonstraram em pequena série de pacientes que, após seis semanas de treinamento em pacientes com DAC, estímulos isquêmicos repetitivos também não resultaram em danos, disfunções miocárdicas e arritmias significativas. ^186 , 187^

Portanto, existem evidências que sugerem a possibilidade da aplicação de treinos intervalados em pacientes com DAC estável, modalidade que tem se revelado segura e efetiva em melhorar o condicionamento físico, a função endotelial e a função ventricular esquerda, acima dos resultados obtidos no treinamento moderado contínuo. ^187 , 188^ Adicionalmente, cabe enfatizar as evidências da superioridade da combinação de treinos aeróbicos e resistidos em relação a treinos aeróbicos isolados nos pacientes com DAC. ^189^

***6.3.4.3. Ajustes de Fármacos Diante da Assimilação do Treinamento Físico***

Os pacientes portadores de DAC estável geralmente utilizam medicamentos para alívio da sintomatologia, redução de isquemia, melhora da função endotelial, estabilização da placa aterosclerótica, controle dos fatores de risco e adequação do padrão hemodinâmico. Por exemplo, níveis elevados de PAS e/ou FC (aumento do duplo produto), aumentando o consumo de oxigênio miocárdico, obviamente contribuem para piorar a tolerância ao esforço e situação clínica.

Nos programas de RCV, particular atenção deve ser dada à melhora do limiar anginoso antes do início do treinamento, já que possibilita maior tolerância à progressão da intensidade de exercícios e, com isso, a obtenção dos efeitos benéficos almejados. Sendo assim, a otimização do tratamento farmacológico é fundamental para uma RCV segura e eficaz.

O paciente submetido à RCV pode apresentar uma série de adaptações fisiológicas relacionadas ao exercício, como, por exemplo, modulação favorável do sistema nervoso autônomo (maior tônus vagal), com maior variabilidade de FC, redução da FC basal, redução do duplo produto de repouso e melhora da função endotelial, ^190^ o que pode significar menor necessidade de fármacos usados no tratamento das DCV, sendo papel do médico de reabilitação discutir com o médico assistente sobre a eventual necessidade de ajustes farmacológicos.

## 6.4. Insuficiência Cardíaca

A IC crônica é uma síndrome complexa que compromete múltiplos sistemas, ocasionando como principais sintomas a dispneia e intolerância progressiva ao esforço físico. Apesar dos recentes avanços na terapêutica farmacológica, com redução da elevada morbimortalidade, os sintomas tendem a persistir, comprometendo a qualidade de vida dos pacientes. Existem evidências consistentes de que a redução do nível de atividade física na IC desencadeia um círculo vicioso, que contribui para aumentar os sintomas e a intolerância ao exercício, secundários à redução da capacidade funcional, produzindo efeitos psicológicos negativos, ^191^ deterioração da vasorreatividade periférica com disfunção endotelial ^192^ e inflamação crônica. ^193^ Nesse contexto, o exercício físico se estabeleceu como estratégia terapêutica segura, que atenua os efeitos do descondicionamento físico progressivo decorrente da evolução natural da doença. ^194^

Estudos randomizados pequenos, revisões sistemáticas e meta-análises têm consistentemente demonstrado que o treinamento físico regular é seguro, aumenta a tolerância aos exercícios, melhora a qualidade de vida e reduz hospitalizações por IC. ^195 - 197^ No entanto, um único e grande estudo randomizado multicêntrico, o HF-ACTION, ^198^ revelou apenas uma modesta, mas não significativa, redução nos desfechos primários de morte e hospitalizações por todas as causas, embora tenha demonstrado benefícios importantes na qualidade de vida e redução da taxa de hospitalizações por IC. Como crítica à pesquisa, há que se considerar que a baixa adesão aos exercícios provavelmente prejudicou a eficácia da intervenção, hipótese que foi confirmada posteriormente em outro estudo, que demonstrou ser a adesão aos exercícios fator determinante para a obtenção de benefícios a médio prazo. ^199^

Em uma revisão sistemática ^2^ sobre treinamento físico em pacientes com IC, que analisou 33 estudos randomizados com inclusão de 4.740 pacientes com predomínio FEVE reduzida, houve tendência à redução da mortalidade total com os exercícios físicos após um ano de seguimento. Comparado ao controle, o grupo de treinamento físico teve menor taxa de hospitalização por IC e melhora da qualidade de vida. Quanto aos benefícios nas mulheres com IC, os estudos disponíveis sugerem que são positivos e equivalentes aos observados nos homens. ^200^

Para pacientes com sintomas avançados (classe IV da NYHA – *New York Heart Association* ), ainda não há dados suficientes para indicar programas de exercício, pois apenas um estudo randomizado brasileiro testou um programa de exercícios diários em cicloergômetro com ventilação não invasiva. Foram avaliados pacientes internados com IC descompensada, sendo observados benefícios funcionais e redução do tempo de internação. ^201^ Portanto, para um grau de recomendação mais forte, há necessidade de mais estudos que confirmem os resultados iniciais.

Na IC com FEVE preservada há evidências recentes provenientes de estudos randomizados pequenos e revisão sistemática que mostraram benefícios no VO _2_ pico medido pelo TCPE, ^202 , 203^ na qualidade de vida ^203 , 204^ e na função diastólica avaliada pelo ecocardiograma. ^205 , 206^

Diante dessas evidências, a RCV com exercícios é recomendada na IC ( Tabela 6 ), quer a FEVE esteja preservada ou reduzida. Assim, políticas públicas devem ser adotadas para que maior número de pacientes elegíveis se beneficie do tratamento em programas estruturados de RCV. ^207^

**Tabela 6:** – Indicação de reabilitação cardiovascular na insuficiência cardíaca

Indicação	Recomendação	Nível de evidência
Exercícios aeróbicos regulares em pacientes com IC para aumentar a capacidade funcional, reduzir sintomas e melhorar qualidade de vida ^2,195-199,205^	I	A
Exercícios aeróbicos regulares em pacientes com FEVE reduzida para diminuir hospitalizações por IC ^2,198^	I	A
Exercícios aeróbicos em pacientes com FEVE preservada para aumentar capacidade funcional e melhorar a função diastólica ^203,205,206^	IIa	B
Exercícios aeróbicos de baixa intensidade na fase hospitalar da IC com ventilação não invasiva ^201^	IIb	B/C


*IC: insuficiência cardíaca; FEVE: fração de ejeção do ventrículo esquerdo.*

Os exercícios físicos, apenas não devem ser prescritos para pacientes com IC clinicamente instáveis, com quadro de miocardite aguda ou na ocorrência de processos infecciosos agudos sistêmicos (Classe IIIC).

### 6.4.1. Prescrição dos Exercícios Físicos e Avaliação Pré-participação

**Figura 2:**
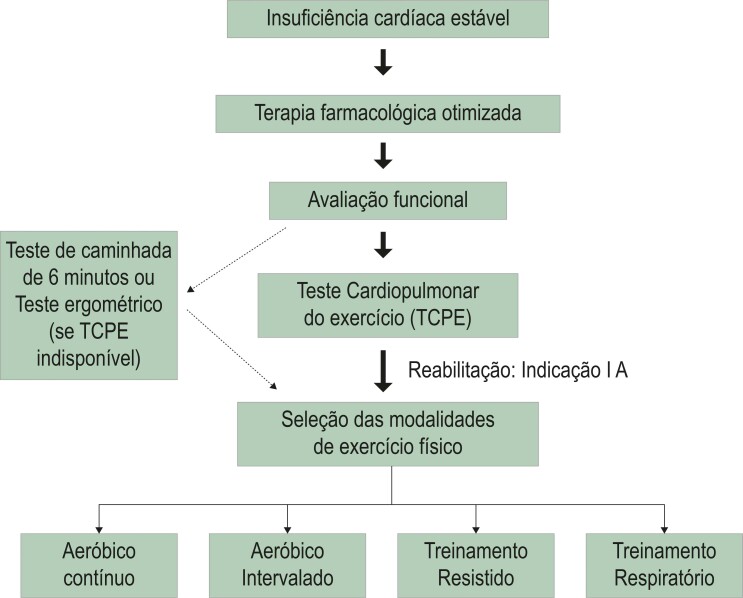
– Fluxograma da reabilitação cardiovascular no paciente com insuficiência cardíaca.

Internacionalmente, os programas de RCV são implementados com vários formatos, utilizando-se modalidades isoladas ou associadas. Os exercícios aplicados podem ser aeróbicos (moderada e/ou alta intensidade), de resistência muscular localizada e treinamento de musculatura respiratória ( Figura 2 ).

Antes de iniciar o programa de treinamento, é fundamental que o paciente esteja com o tratamento farmacológico otimizado e clinicamente estável; o ideal é que realize uma avaliação funcional, preferencialmente com TCPE ou TE. Na indisponibilidade das provas funcionais citadas, o teste de caminhada de 6 minutos pode servir de parâmetro de acompanhamento dos ganhos funcionais. ^208^ Os testes funcionais devem ser realizados em uso das medicações prescritas para mimetizar a condição que estará presente durante o treinamento.

Os treinamentos aeróbicos recomendados podem ser contínuos de moderada intensidade (TCMI), que correspondem à zona de FC delimitada pelos limiares ventilatórios do TCPE, ou, no caso do TE, à zona situada entre 60 e 80% da FC pico ou 50 e 70% da FC de reserva. Pacientes mais graves e com maior limitação funcional podem iniciar no limite inferior da prescrição. Progressões de intensidade até o limite superior podem ser realizadas com a evolução do treinamento.

Recentemente, tem aumentado a utilização de exercícios aeróbicos de alta intensidade realizados de modo intervalado, denominado de treinamento intervalado de alta intensidade (TIAI). Ele alterna períodos mais intensos com momentos de recuperação passiva ou ativa, o que possibilita maior duração total de exercícios na alta intensidade e, consequentemente, pode produzir maior estímulo para adaptações fisiológicas centrais e periféricas.

Em pacientes com IC e FEVE reduzida, Wisløff et al. ^209^ demonstraram que o TIAI foi superior ao TCMI em promover a melhora na capacidade funcional e em diferentes parâmetros cardiovasculares. Posteriormente, outros ensaios clínicos foram realizados e meta-analisados. No que tange ao efeito do TIAI sobre a capacidade funcional, a superioridade do método em relação ao TCMI foi confirmada em uma meta-análise. ^210^ Entretanto, o maior estudo multicêntrico publicado, o Smartex-HF, ^211^ comparou as modalidades de exercícios contínuos de moderada intensidade com os intervalados de alta intensidade. A conclusão foi que os benefícios são semelhantes, não havendo superioridade de modalidade em nenhum aspecto. Portanto, a escolha do protocolo vai depender de experiência da equipe, condições clínicas, capacidade física e preferências do paciente.

Além disso, o modo de utilizar o TIAI pode variar bastante, com vários protocolos descritos. ^212^ Um deles é composto por 4 minutos de exercícios de alta intensidade (90 a 95% da FC máxima), alternados com 3 minutos de leve intensidade (70% da FC máxima). ^209^ Protocolos com duração da carga intensa bem menores, com 30 ou 90 s, já foram descritos, e a tolerância a diferentes modelos de TIAI pode variar de acordo com a escolha e a capacidade física do paciente. ^213^ Portanto, a utilização e o modo de execução vai depender das características clínicas e escolhas do paciente, bem como da experiência e das preferências da equipe de RCV.

Além do treinamento aeróbico, a adição de exercícios de resistência muscular localizada tem sido sugerida para obtenção de benefícios adicionais. ^214^ Eles podem ser prescritos como percentuais da contração voluntária máxima ou de acordo com a percepção subjetiva ao esforço. As cargas e repetições recomendadas podem variar de acordo com as limitações funcionais do paciente e devem ser individualizadas, com progressão de acordo com evolução na RCV.

Os exercícios respiratórios têm sido indicados para programas de treinamento de pacientes com fraqueza da musculatura respiratória. ^215^ A meta-análise de Smart et al., ^216^ que avaliou 11 estudos com 287 participantes com IC, sendo 148 submetidos ao treinamento da musculatura inspiratória (TMI) comparados com 139 controles sedentários, mostrou significativos ganhos devidamente documentados: a) no TCPE, pelo aumento de consumo de oxigênio miocárdico no pico do esforço (VO _2_ pico) e melhora da eficiência ventilatória observada na relação da ventilação pulmonar com a produção de dióxido de carbono (VE/VCO _2_ slope); b) na espirometria, pelo aumento da pressão inspiratória máxima; c) no teste de caminhada de 6-minutos, pela maior distância percorrida; d) melhora da qualidade de vida. Portanto, o TMI proporcionou ganhos da aptidão cardiorrespiratória e na qualidade de vida de similar magnitude aos obtidos com o treinamento convencional, devendo ser considerado alternativa válida para os pacientes com IC gravemente descondicionados fisicamente e muito debilitados, em uma transição para os exercícios físicos convencionais.

### 6.4.2. Considerações Finais

É fundamental que pacientes com IC realizem exercícios físicos, idealmente com prescrição individualizada, no contexto de um programa de RCV, levando-se em consideração a combinação de treinamentos aeróbicos de moderada e/ou alta intensidade, exercícios de resistência muscular localizada e treinamento da musculatura respiratória (treinamento ventilatório). Para isso, devem ser levadas em consideração, além do quadro clínico e limitações funcionais a ele relacionadas, preferências do paciente e a experiência da equipe. Por fim, é relevante ressaltar a existência de alternativas válidas mesmo para os pacientes muito debilitados e gravemente descondicionados. ^214 , 217^

## 6.5. Transplante Cardíaco

O transplante cardíaco (TxC) é o tratamento de escolha para pacientes com IC refratária, que permanecem com sintomas graves mesmo em uso de todo o arsenal farmacológico disponível e na realização de procedimentos cirúrgicos indicados.

Nos últimos anos ocorreram avanços significativos no TxC, com surgimento de novas técnicas cirúrgicas e desenvolvimento de substâncias imunossupressoras mais eficientes. No Brasil, houve um crescimento substancial na quantidade de procedimentos, o que estava estagnado desde 2015, com taxa de 1,7 TxC por milhão da população (pmp). Em 2019 houve um crescimento de 17,6%, chegando a 2 TxC pmp, muito próximo da meta estabelecida para o ano (2,1 pmp). Em 2018 foram realizados 357 procedimentos, e até março de 2019, 104 corações já foram transplantados no Brasil. ^218^

O TxC tem como objetivo promover a melhora na qualidade de vida, assim como aumento da sobrevida. ^219 , 220^ Os receptores são capazes de retornar ao trabalho e ter uma vida normal, com mínimos sintomas ou mesmo assintomáticos. ^221^ A taxa de sobrevida no 1º ano é estimada em 90% e em 5 anos em cerca de 70%. ^222^

Embora o TxC melhore significativamente a capacidade funcional dos pacientes, o VO _2_ pico ainda se encontra reduzido quando comparado ao de indivíduos saudáveis, pareados por idade. ^223 , 224^ Dentre outros fatores, isso pode ser explicado por: 1) imediatamente no período pós-transplante, o aloenxerto apresenta ausência de inervação simpática e parassimpática (denervação autonômica), provocando aumento da FC de repouso, o que atenua a sua elevação natural como resposta ao exercício e prejudica a recuperação após o esforço ^224 , 225^ ; 2) ocorrência de disfunção muscular esquelética (às vezes chegando à caquexia), na qual a terapia imunossupressora, associada à IC prévia, exerce papel de destaque ^226^ ; 3) comprometimento da função vascular e diastólica. ^227^ Em pacientes com TxC, na fase aguda do exercício, o aumento do débito cardíaco depende fundamentalmente do mecanismo de Frank-Starling, do aumento do retorno venoso, do inotropismo, do cronotropismo e da redução da pós-carga. ^228 , 229^ Além disso, ocorre aumento das concentrações de catecolaminas circulantes, ^227^ que reduzem lentamente após o término do exercício, justificando uma lenta recuperação da FC. ^230^

A imunossupressão pode predispor a maior risco de outras complicações, ^231^ e os pacientes transplantados podem evoluir com desenvolvimento de HAS, diabetes melito e coronariopatia. ^232^ Por sua vez, o treinamento físico é conhecido como terapêutica de excelência para o manejo dessas doenças crônicas, ^93 , 233^ sendo eficaz na otimização do controle autonômico. ^230 , 234^

O treinamento físico após o TxC contribui para o aumento do VO _2_ pico e a melhora do controle hemodinâmico, da força muscular e da densidade mineral óssea, ^233 - 236^ contribuindo para melhorar o prognóstico. ^19^ Embora existam inúmeras possibilidades de prescrição de treinamento, o principal método preconizado permanece sendo o exercício aeróbico, que pode ser realizado de maneira contínua ou intervalada, ^170^ sempre que possível devem ser realizados também exercícios resistidos. ^6^

### 6.5.1. Benefícios dos Exercícios Físicos

No estudo pioneiro de Richard et al., ^237^ os pesquisadores observaram que, após um período de 46 meses pós TxC, pacientes que realizaram treinamento aeróbico, apresentaram capacidade funcional e função cronotrópica semelhantes às verificadas em indivíduos saudáveis. ^234 , 238 - 240^

Uma meta-análise da Cochrane, que reuniu nove ensaios clínicos randomizados, totalizando 284 pacientes, comparou o efeito do treinamento físico aos cuidados usuais em pacientes pós TxC, ^234^ evidenciando aumento médio do VO _2_ pico de 2,5 ml.kg ^- 1^ .min ^- 1^ nos que realizaram treinamento, em relação aos alocados para cuidados usuais. Rosembaun et al. ^241^ estudaram a relação entre a participação precoce em um programa de RCV de pacientes após TxC e verificaram que o número de sessões realizadas nos primeiros 90 dias esteve relacionado diretamente com melhor sobrevida em 10 anos.

Haykowsky et al. ^242^ descreveram melhoras significativas no VO _2_ pico de pacientes após TxC, com aumento médio de 3,1 ml.kg ^- 1^ .min ^- 1^ após 12 semanas de treinamento combinado (resistido e aeróbico). Kobashigawa et al. ^243^ estudaram 27 pacientes após TxC, os quais foram submetidos a uma combinação de treinamento aeróbico, resistido e de flexibilidade durante 6 meses *versus* grupo-controle. A duração e a intensidade das sessões de exercícios aeróbicos tiveram como meta, no mínimo, 30 min de exercício contínuo de intensidade moderada em bicicleta estacionária. O grupo intervenção apresentou aumento médio de 4,4 ml ^- 1^ .kg ^- 1^ .min no VO _2_ pico, *versus* 1,9 ml.kg ^- 1^ .min ^- 1^ no grupo-controle. Esses dados fornecem informações valiosas da importância de ambos os tipos de treinamento após TxC.

Em relação ao treinamento de alta intensidade em pacientes após TxC, os resultados são motivadores, mas ainda há um pequeno número de estudos. Em um estudo *crossover* , Dall et al. ^244^ verificaram efeito superior do TIAI em relação ao TCMI no VO _2_ pico, com ganho adicional de 2,3 ml.kg ^- 1^ .min ^- 1^ e melhora superior na qualidade de vida. Em meta-análise que reuniu três ensaios clínicos randomizados que compararam o TIAI (blocos intensos: 80 a 100% do VO _2_ pico ou 85 a 95% da FC pico) aos cuidados usuais, os pacientes que realizaram TIAI apresentaram aumento adicional no VO _2_ pico de 4,45 ml.kg ^- 1^ .min ^- 1^ após o período de intervenção, que variou de 8 a 12 semanas, com três a cinco sessões semanais. ^233^

Nytrøen et al. ^224^ avaliaram os efeitos de um programa de TIAI após TxC em 43 pacientes. Foram observados os efeitos na progressão da vasculopatia do enxerto, avaliada por ultrassom intracoronário, constatando menor progressão de placas de ateroma no grupo TIAI. Porém, mais estudos ainda são necessários para esclarecer melhor os benefícios dessa modalidade de treinamento. ^245^

Sabe-se que alguns dos efeitos adversos comuns ao uso de glicocorticoides após o TxC são atrofia e fraqueza musculares. Em 1998, Braith et al. ^235^ estudaram, pela primeira vez, o efeito do treinamento resistido na miopatia induzida por glicocorticoide em receptores de TxC. Um grupo realizou treinamento e foi comparado com um grupo-controle. Após 6 meses, apesar de ambos terem apresentado aumento na força muscular do quadríceps e dos extensores lombares, houve um aumento até 6 vezes maior no grupo treinado.

O treinamento resistido também parece ter importante efeito terapêutico para melhoria do metabolismo ósseo. Após o TxC, os pacientes não raramente apresentam perda óssea significativa na cabeça do fêmur e perda óssea mineral total. Em pacientes arrolados para treinamento resistido, após 2 meses da realização do TxC, o treinamento de força se mostrou capaz de restaurar a densidade mineral óssea a níveis pré-transplante. ^236^

### 6.5.2. Avaliação Pré-participação e Particularidades

Os pacientes após TxC devem realizar anamnese, exame físico, ECG de repouso de 12 derivações, ecocardiograma com Doppler colorido ou outros exames a critério dos profissionais envolvidos. O ideal é a realização de um teste funcional em exercício, preferencialmente o TCPE realizado por médico experiente com o método. O TCPE considerado o padrão-ouro para avaliação da capacidade funcional, permite a determinação das respostas cardiopulmonar e metabólicas ao esforço físico, por meio de diversas variáveis que são de grande utilidade para a avaliação clínica e prescrição otimizada dos exercícios físicos. ^246^ Os fisioterapeutas e profissionais de educação física devem atuar na prescrição, aplicação, supervisão e orientação dos exercícios, mas seguindo os limites de segurança recomendados pelos médicos responsáveis pela avaliação pré-participação. ^6 , 247^

A impossibilidade da realização do TCPE não deve ser um impedimento para prática dos exercícios; na ausência dele, sugere-se um TE convencional. ^170^ Quando nem mesmo este estiver disponível, o teste de caminhada de 6 minutos poderá auxiliar na avaliação clínica e prescrição de exercício, sendo parâmetro válido de comparação da capacidade funcional no decorrer do treinamento. ^248 , 249^

### 6.5.3. Prescrição do Treinamento Físico

O treinamento aeróbico, ressaltando que o TCMI tem sido o método usado na maioria dos estudos, tem sido o mais preconizado, devendo ser complementado pelo treinamento resistido a partir da 6ª semana após TxC. Mas, diferentes metodologias de treinamento vêm sendo estudadas de maneira isolada ou combinada e têm se mostrado eficazes na promoção da saúde cardiovascular nos indivíduos em RCV, abrindo um leque de possibilidades a serem consideradas. ^6 , 170^

De acordo com a condição clínica do paciente, a intensidade do exercício aeróbico pode aumentar gradualmente de moderada a alta ao longo do treinamento, pois a intensidade está diretamente associada à magnitude das desejáveis adaptações cardiovasculares. ^250^ Nesse sentido, programas que incluíram o treinamento intervalado, até mesmo de alta intensidade, mostraram bons resultados. ^233^ Porém, para uma otimizada e segura prescrição, deve haver adequada individualização de cada componente da sessão de treinamento. ^170^

A determinação de zonas-alvo de treinamento é recomendável, visando otimização da prescrição do exercício. ^170^ Entretanto, tendo em vista a resposta cronotrópica ainda comprometida, ^251^ a prescrição com base nos percentuais da FC máxima ou nas FC dos limiares não é possível nas primeiras sessões de treinamento, podendo ser úteis quando ocorre melhora na resposta autonômica. ^224^ Por esse motivo, a contínua avaliação do comportamento da FC durante o exercício e na recuperação se torna de suma importância. Quando um TCPE for disponível, a prescrição do exercício aeróbico poderá ser pautada nas cargas atingidas nos limiares ventilatórios ou nos percentuais estabelecidos do VO _2_ pico. Outra estratégia simples e viável é a avaliação da percepção subjetiva do esforço, por meio da escala BORG, ^4 , 170 , 252^ devendo haver empenho da equipe multiprofissional em educar o paciente em relação à percepção de esforço e às manifestações de sintomas. ^4 , 6^

Além da avaliação e prescrição dos exercícios aeróbicos, é fundamental a realização de exercícios resistidos. Tradicionalmente têm sido utilizados, para a avaliação e prescrição destes exercícios, os testes de carga de uma repetição máxima, cuja aplicação, entretanto, é questionável, principalmente após o TxC recente, pois sua segurança carece de investigações clínicas nos transplantados. Uma alternativa seria o teste de sentar e levantar da cadeira em 30 segundos, ^253^ que foi validado em idosos ativos e se mostrou razoavelmente confiável em fornecer informações sobre a força de MMII, sendo bastante utilizado em centros de reabilitação e em estudos científicos de diferentes condições clínicas. ^254 - 256^

Uma possibilidade de grande aplicabilidade é a prescrição dos exercícios físicos resistidos de modo subjetivo, segundo a percepção de esforço considerado moderado, associado ao método de repetição variável, com o objetivo de executar uma faixa de repetições (por exemplo, de 10 a 15 repetições). Se o paciente não conseguir executar o mínimo, a carga aplicada está elevada; se executar o máximo de modo fácil, a carga está leve. Desse modo, a carga pode ser ajustada para que o treinamento seja realizado dentro da faixa de repetições proposta.

Durante o treinamento, especial atenção deve ser dada às complicações como as infecções relacionadas ao procedimento do transplante. Em levantamento realizado nos Estados Unidos, foi evidenciado que 36% dos receptores são hospitalizados ao longo do primeiro ano, e 61%, dentro de um período de 4 anos após TxC, ^257 , 258^ o que deixa evidente a importância da supervisão dos pacientes ao longo do treinamento, com eventual necessidade de que as sessões sejam temporariamente interrompidas. Tendo em vista o exposto, alguns autores sugerem que os pacientes não devam realizar exercício físico durante o período de administração de terapia com pulsos de esteroides e nos dias de biópsia miocárdica. ^170^

### 6.5.4. Reabilitação Cardiovascular Domiciliar

Estudos têm demonstrado que os programas de RCVD são seguros e efetivos, ^1^ sendo recomendáveis como uma alternativa para a RCV tradicional em pacientes de menor risco. ^71^

Wu et al. ^259^ conduziram um estudo prospectivo e randomizado para avaliar o efeito de um programa de exercícios domiciliares durante 2 meses em 37 pacientes após TxC. O grupo controle manteve o estilo de vida habitual durante o período de estudo. Os indivíduos do grupo intervenção realizaram um programa de exercícios no mínimo três vezes na semana, que englobou 5 min de aquecimento, atividades de fortalecimento de MMSS e MMII, 15 a 20 min de exercício aeróbico em intensidade de 60 a 70% do VO _2_ pico, além de 5 min de desaquecimento. Para garantir a execução domiciliar correta, inicialmente foi realizado um período supervisionado para orientação e prescrição. Ao final de 2 meses, os pacientes melhoraram força e resistência muscular, índice de fadiga e qualidade de vida no domínio físico. Por meio do TCPE, foi observado aumento da carga de trabalho, mas sem modificação do VO _2_ pico, provavelmente pelo curto período de seguimento ou pela metodologia da prescrição do treinamento, que foi de menor intensidade. Outro estudo, ^260^ com protocolo de treinamento aeróbico equivalente, porém maior duração, cinco vezes por semana durante 6 meses, documentou melhora no VO _2_ pico, na carga de trabalho e na PA de indivíduos após TxC. Além disso, ocorreram sinais de reinervação simpática cardíaca e restauração da sensibilidade à modulação autonômica nas artérias, sendo que nenhuma alteração foi observada no grupo controle.

Mesmo com período superior a 5 anos após o TxC, a RCVD melhora a capacidade funcional, conforme demonstra um estudo em que 21 pacientes foram instruídos a realizar um programa de treinamento físico domiciliar por 1 ano em bicicleta ergométrica, enquanto nove pacientes serviram como controle. Para garantir o adequado controle, os pacientes receberam um cartão inteligente, programado para um aquecimento de 6 min e uma carga de trabalho constante durante 20 min, com ajuste de carga de acordo com a prescrição e o monitoramento da FC. Ao final de 12 meses, houve modesta melhora no VO _2_ pico. ^261^

Karapolat et al. ^262^ em estudo publicado em 2008, compararam os efeitos de programa domiciliar e presencial sobre a capacidade de exercício e variáveis cronotrópicas em 28 pacientes após TxC. Foram observadas melhoras significativas no VO _2_ pico e na FC de reserva apenas no grupo da RCV tradicional. Porém, novos estudos, com inclusão de um maior número de pacientes, são necessários para melhor elucidação desta superioridade da RCV presencial observada neste estudo.

### 6.5.5. Recomendações

Com base nas diversas evidências expostas, o efeito benéfico do treinamento físico em indivíduos após TxC é inequívoco e essa terapia se mostra segura e exequível, podendo ser realizada no ambiente hospitalar ou domiciliar ( Tabela 7 ). No entanto, embora ambas as estratégias sejam eficazes em promover aumento na capacidade funcional, existem indícios de que a magnitude do efeito seja maior quando o treinamento é realizado em ambientes supervisionados.

**Tabela 7:** – Indicação de reabilitação cardiovascular no transplante cardíaco

Indicação	Recomendação	Nível de evidência
RCV com exercícios aeróbicos moderados são recomendados para pacientes após TxC ^234,239,241,243^	I	A
RCV com exercício aeróbico de alta intensidade é recomendada para pacientes após TxC ^233,238,244^	IIa	B
RCV com exercícios físicos resistidos é recomendada para pacientes após TxC ^235,236^	I	B

*RCV: reabilitação cardiovascular; TxC: Transplante cardíaco.*

A RCV deve ser iniciada entre 6 e 8 semanas após o TxC, sendo o direcionamento realizado na alta hospitalar. Em casos selecionados e após criteriosa avaliação da equipe, o início pode ser mais precoce. Assim como em qualquer situação na qual o paciente seja submetido a esternotomia, um cuidado especial em relação a não realizar exercícios que sobrecarreguem a musculatura torácica e levem à tração do esterno deve ser salientado, principalmente nos primeiros 90 dias após o procedimento cirúrgico.

A prescrição ideal inclui exercícios para promoção das diferentes valências físicas, sempre enfatizando o que é preconizado para cada condição. No cenário após Txc, assim como em outras indicações de RCV, o exercício aeróbico é a parte principal das sessões de treinamento, devendo ser complementado pelos resistidos e de flexibilidade, dentro de um programa individualizado e periodizado. As sessões devem sempre iniciar com um período de aquecimento, assim como encerrar com um desaquecimento controlado. Tal estratégia visa, além do aquecimento muscular, um período para ajuste da FC e da PA, que estão alteradas nesses pacientes pela denervação do coração, especialmente no início do programa de treinamento após o procedimento.

O exercício aeróbico pode ser realizado em forma de caminhada ou ciclismo, tanto *indoor* , utilizando recursos como esteiras e/ou bicicletas ergométricas, ou quanto *outdoor* . Recomenda-se frequência semanal de três a cinco sessões, com duração de 20 a 40 minutos. A frequência e duração das sessões serão ajustadas conforme condições prévias do paciente e devem progredir ao longo do treinamento. O controle da intensidade é fundamental e, devido ao maior número de evidências, preconiza-se o TCMI (Entre o 1º e o 2º limiar ventilatório), com uma percepção de esforço referida entre 11 e 13 na Escala Borg modificada. O treinamento intervalado pode ser adotado, em casos selecionados, com objetivo de variação na forma do treino e busca de um ganho funcional potencialmente maior.

Os exercícios resistidos têm papel fundamental, principalmente na fase inicial após TxC. Muitos apresentaram IC de longa duração, estiveram internados por longos períodos e passaram pelo estresse cirúrgico. No início do treinamento, atividades sem carga externa, ou seja, apenas com peso corporal, podem ser consideradas como estímulo suficiente para esses pacientes. Em seguida, bandas elásticas, halteres, caneleiras e aparelhos de musculação podem ser incluídos no programa de treinamento. Maior cuidado deve ser dado aos exercícios de MMSS, devido à toracotomia, levando em consideração que, com o uso de corticosteroides, o período de cicatrização pode ser maior.

Outras informações e exemplos de protocolos de treinamento nesses pacientes podem ser obtidos em outras publicações. ^263 - 265^

## 6.6. Miocardiopatias

Nesta seção, serão abordadas a miocardiopatia hipertrófica (MCH), a miocardite e outras miocardiopatias, cujas indicações de RCV estão listadas na Tabela 8 .

**Tabela 8:** – Indicação de exercícios físicos nas miocardiopatias

Indicação	Recomendação	Nível de evidência
Exercícios aeróbicos moderados, para pacientes selecionados, com MCH ^266,267^	IIa	B
Exercício físico vigoroso ou competitivo para pacientes com MCH ^268,269^	III	C
Exercícios aeróbicos moderados, para pacientes selecionados, após 3 a 6 meses do quadro agudo de miocardite	IIb	C
Exercícios aeróbicos leves a moderados para pacientes selecionados com CAVD ^270^	IIb	B
Exercícios físicos de alta intensidade ou competitivos para pacientes com CAVD ^268,269^	III	C

*MCH: miocardiopatia hipertrófica; CAVD: cardiomiopatia arritmogênica do ventrículo direito.*

### 6.6.1. Miocardiopatia Hipertrófica

A MCH é uma doença caracterizada por hipertrofia do ventrículo esquerdo, geralmente com câmaras ventriculares não dilatadas, na ausência de outra doença cardíaca ou sistêmica capaz de produzir a magnitude da hipertrofia evidenciada, ^271^ sendo a doença cardíaca herdada mais comum na população e causada por uma gama de mutações de genes responsáveis pelas proteínas do sarcômero cardíaco. ^268^ A principal característica é uma expressão clínica heterogênea, com alterações fisiopatológicas peculiares e uma história natural variável. Até 10% dos casos são causados por outras doenças genéticas, incluindo metabólicas e neuromusculares hereditárias, anormalidades cromossômicas e síndromes genéticas. ^272^ Alguns pacientes apresentam outros distúrbios que podem mimetizar formas da doença, como, por exemplo, amiloidose. ^273^

A prevalência populacional é estimada em torno de 0,2% ou 1:500. ^268^ No entanto, essa estimativa parece ser distinta na prática clínica, o que permite inferir que uma parcela dos indivíduos afetados são assintomáticos. Diversos padrões de hipertrofia assimétrica do ventrículo esquerdo são comuns à MCH, e pode haver fenótipos diversos em familiares de primeiro grau. Tipicamente, uma ou mais regiões do ventrículo esquerdo têm espessura parietal aumentada quando comparadas com outras, e podem ocorrer transições e variações de espessura em áreas adjacentes ou áreas não contíguas. Contudo, apesar da hipertrofia septal assimétrica ser a mais comumente debatida, não existe um padrão clássico de MCH e, virtualmente, todos os padrões possíveis de hipertrofia ventricular esquerda podem ocorrer. Mesmo a ausência de hipertrofia pode ser encontrada em indivíduos geneticamente acometidos (fenótipo negativo).

Diferentes estudos de coorte retrospectivos e observacionais, de populações multicêntricas, esclareceram a história natural e o curso clínico dessa cardiopatia. Alguns mais recentes têm mostrado mortalidade anual em torno de 1%, valor muito menor do que em pesquisas mais antigas. ^274^ Notavelmente, apenas em um pequeno subgrupo de pacientes com MCH ocorre morte prematura e complicações significativas relacionadas à doença, as quais podem ocorrer por obstrução da via de saída do ventrículo esquerdo, IC com disfunção diastólica e/ou sistólica e morte súbita (MS) ou arritmias cardíacas (fibrilação atrial e taquicardia ou fibrilação ventricular). ^275^ A MS na MCH pode acontecer em qualquer faixa etária, embora seja mais comum em adolescentes e adultos jovens; por isso, a identificação de indivíduos sob maior risco é muito importante na avaliação pré-participação esportiva. ^276^

Em muitos casos, a MS pode ser a primeira manifestação da doença nesses indivíduos, ocorrendo mais comumente naqueles sem sintomas de alerta, os quais não haviam sido diagnosticados previamente ao evento. Entretanto, a maioria dos pacientes com MCH apresenta uma expectativa de vida normal ou quase normal, com mortalidade relacionada a outras doenças, algumas, inclusive, de etiologia não cardiovascular. ^277 - 279^ Portanto, incentivar um estilo de vida saudável para pacientes com MCH é essencial para reduzir o risco global de doença.

***6.6.1.1. Benefícios Terapêuticos do Exercício Físico***

O nível de aptidão cardiorrespiratória está associado ao risco de mortalidade cardiovascular e por todas as causas na população em geral. ^19^ Em paciente com MCH, forma obstrutiva e minimamente sintomática, também já foi observada associação de mortalidade com a aptidão aeróbica. ^280 , 281^ Pacientes com VO _2_ pico abaixo de 18 ml.kg ^- 1^ .min ^- 1^ no TCPE apresentaram maior mortalidade e sintomatologia mais exuberante em comparação aos que obtiveram valores iguais ou superiores. A presença de VO _2_ pico inferior a 60% do previsto significou pior sobrevida em 4 anos, em torno de 60%. ^280^

O aumento da fibrose miocárdica e o desarranjo miofibrilar podem estar por trás do risco aumentado de MS na MCH, pois essa alteração atua como substrato para arritmias fatais. ^271^ Evidências sugerem que o treinamento físico intenso poderia acelerar tais alterações, mas esse ainda é um tema controverso. Entretanto, sabe-se que o aumento da fibrose miocárdica está associado a menor VO _2_ pico nessa população. ^282^

Sendo assim, avaliar a aptidão aeróbica, preferencialmente pelo TCPE, é importante nos pacientes com MCH. ^281^ Quando existe redução do VO _2_ pico, a prática de exercícios físicos pode contribuir para aumentar a capacidade funcional.

Até o momento, apenas um ensaio clínico randomizado controlado examinou o efeito do treinamento físico em pacientes com MCH (RESET-MCH). Esse estudo, que contemplou 136 pacientes, demonstrou aumento no VO _2_ pico após treinamento de moderada intensidade depois de 16 semanas de intervenção (+ 1,35 ml.kg ^- 1^ .min ^- 1^ ou < 0,5 MET). ^266^ Outro estudo prospectivo não randomizado incluiu 20 pacientes com MCH e mostrou aumento significativo na duração do TE, assim como na capacidade funcional estimada (+ 2,5 MET). ^267^ Nesse estudo, os pacientes completaram um programa de RCV com sessões de 60 min de exercício moderado a vigoroso, realizados em esteira ou cicloergômetro, 2 vezes por semana. A intensidade do exercício progrediu de 50 para 85% da FC de reserva, o que resultou em aumento gradual do condicionamento e pode ter minimizado o risco de eventos adversos, como arritmias induzidas pelo exercício. Em nenhum desses estudos com treinamento houve ocorrência de eventos adversos sérios, como morte, MS cardíaca abortada, terapia do cardioversor desfibrilador implantável (CDI) ou taquicardia ventricular sustentada. ^266 , 267^

***6.6.1.2. Quando indicar exercícios físicos***

A intensidade de exercício liberado para pacientes com MCH representa um grande desafio. Se por um lado, o exercício físico intenso pode ser deletério, com aumento do risco de arritmias potencialmente fatais; por outro, a restrição excessiva de atividade física conduz ao descondicionamento e pode ter efeitos negativos na saúde e na qualidade de vida do paciente, podendo até aumentar o risco cardiovascular, visto que existe associação entre aptidão física e mortalidade. ^280 , 281^

A *American Heart Association* , em seu posicionamento oficial para o tratamento da MCH, desencoraja os pacientes com a doença a se envolverem em esportes competitivos de intensidade moderada a vigorosa (ver Tabela 8 ). A limitação serviria para minimizar as mudanças súbitas na PA e no aumento do débito cardíaco, de modo a, supostamente, proteger dos efeitos negativos do exercício em um coração patologicamente hipertrofiado. ^283^

O exercício como gatilho para arritmias em curto prazo e remodelamento adverso em longo prazo são os efeitos mais temidos na MCH. O receio da MS cardíaca durante o esporte estende-se a atividades atléticas não competitivas, embora exista uma clara falta de evidência sobre a segurança do exercício neste perfil de pacientes. No entanto, deve ser ressaltado que esse risco dos exercícios é teórico e as recomendações para as limitações da atividade física têm sido advogadas com cautela, pela opinião de especialistas e não por evidências mais robustas. ^284^

Desse modo, os pacientes com MCH recebem pouca ou nenhuma orientação em relação à melhor dose ou quantidade de atividade física para manutenção da saúde geral e do bem-estar, sendo que maior foco é dado nas restrições às atividades físicas. Como resultado, mais de 50% dos pacientes com MCH não alcançam o mínimo de atividade física recomendada, devido à crença de que são incapazes de exercê-la e/ou de que a atividade física pode piorar a doença.

Sendo assim, o equilíbrio parece ser o mais adequado e os extremos devem ser evitados (nem exercício vigoroso competitivo, nem sedentarismo), pois ambos poderiam aumentar o risco cardiovascular.

Em virtude das novas evidências, pode-se considerar haver efeito positivo dos exercícios físicos moderados em pacientes selecionados com MCH, com a avaliação de risco e a prescrição realizadas individualmente. Ressalta-se que as evidências sugerem benefícios para o treinamento moderado contínuo, sendo que outras modalidades ainda devem ser mais estudadas.

Entretanto, a presença das seguintes características poderiam ser consideradas contraindicações maiores à prática de exercício: história de MS abortada e ausência de CDI; história de síncope aos esforços; ocorrência de taquicardia ventricular induzida pelo exercício; aumento do gradiente com o exercício (superior a 50 mmHg) e resposta pressórica anormal ao esforço.

***6.6.1.3. Avaliação Pré-participação***

A definição da liberação para iniciar os exercícios deve ser realizada pela avaliação médica pré-participação, realizando-se anamnese, exame físico e ECG de 12 derivações.

Uma grande parcela dos indivíduos com MCH é assintomática ou oligossintomática e a suspeita clínica é dada por alterações no ECG de repouso, o qual é anormal em até 95% dos pacientes com a doença. ^285^ As alterações eletrocardiográficas podem preceder doença estruturalmente detectada por alguns anos, o que torna a realização do ECG de suma importância nesse cenário. ^269^ Somente uma minoria dos pacientes com MCH apresentam ECG normal, em geral aqueles sem outra manifestação fenotípica da doença (genótipo positivo/fenótipo negativo).

O ecocardiograma segue como o exame mais empregado para o diagnóstico de MCH, restando a ressonância magnética nuclear (RMN) como alternativa para os casos em que o primeiro não for conclusivo, ou para avaliar situações de hipertrofia mais localizada (formas apicais, por exemplo). Em atletas jovens, a diferenciação entre a hipertrofia fisiológica do coração de atleta e a hipertrofia patológica da MCH é um desafio. Isso porque, tal como acontece com indivíduos sedentários com MCH, a maioria dos atletas com a doença mostra um padrão assimétrico de hipertrofia do ventrículo esquerdo. Em contraste, aqueles com hipertrofia fisiológica do ventrículo esquerdo mostram distribuição mais homogênea e simétrica da espessura da parede, com apenas pequenas diferenças entre os segmentos contíguos e um padrão simétrico de hipertrofia do ventrículo esquerdo. ^286^

O teste de exercício, previamente ao início da RCV, está sempre recomendado nesses pacientes, seja para avaliação da capacidade funcional ou para detecção de respostas anormais da PA e sinais de aumento da obstrução dinâmica da via de saída com o esforço. Para melhor detecção de obstrução da via de saída durante o exercício, a associação de exame de imagem (ecocardiograma) com teste de esforço é a melhor modalidade disponível e deve ser encorajada sempre que possível. Pacientes com ausência de obstrução no repouso podem apresentar gradientes significativos no esforço, sendo reclassificados em relação ao prognóstico. ^287^

Quando estiver disponível, sugere-se a realização do TCPE para uma melhor avaliação em esforço, com medida direta do VO _2_ pico, em virtude do seu documentado valor prognóstico. ^280 , 281^ Além disso, a obtenção dos limiares ventilatórios contribui para uma prescrição mais individualizada.

**6.6.1.4. Particularidades na Prescrição e no Acompanhamento dos Exercícios Físicos**

Algumas particularidades dos exercícios em pacientes com MCH devem ser destacadas:

- Atividades do tipo “explosão” (p. ex., basquete, futebol e tênis), em que há potencial para rápida aceleração e desaceleração, devem ser evitadas;
- Atividades com consumo de energia estável e constante (p. ex., corrida leve ou natação) são preferidas;
- Exercício em condições ambientais adversas, incluindo calor ou frio extremos, deve ser evitado, pois há um aumento do risco de exacerbação das alterações fisiológicas induzidas pelo exercício;
- Programas de treinamento que visem competitividade, ou obtenção de níveis mais altos de condicionamento físico e excelência, devem ser evitados, pois normalmente motivam os pacientes a se esforçarem além dos limites.
- Exercícios estáticos (isométricos) intensos, como levantamento de peso, devem ser evitados, pois há risco aumentado de provocar obstrução da via de saída do ventrículo esquerdo, devido à intensa manobra de Valsalva;
- Treinamento resistido com baixa carga e maior número de repetições foi considerado seguro para pacientes com DCV, embora não haja evidência sólida do seu uso na MCH.

Em relação ao uso de medicações, algumas observações são importantes. O uso de betabloqueadores e antagonistas do cálcio pode estar indicado no tratamento da MCH. Como essas medicações atenuam a resposta da FC, é possível ocorrer uma resposta cronotrópica muito reduzida ao esforço, o que pode ocasionar aumento da intolerância ao exercício e indicar necessidade de ajuste da medicação. O uso de diuréticos em excesso pode ser deletério por aumentar o gradiente da via de saída. Sendo assim, devem ser utilizados com cautela. Do mesmo modo que os diuréticos, a desidratação pelo exercício pode elevar o gradiente. Assim, é importante atenção a uma adequada hidratação durante o treinamento.

### 6.6.2. Miocardite

A patogênese da miocardite consiste em três fases: lesão aguda, geralmente de etiologia viral; resposta imune do hospedeiro; e recuperação, ou transição para fibrose e miocardiopatia dilatada, sendo que, clinicamente, não existe uma distinção clara entre essas fases. A lesão inicial pode causar dano agudo ao miocárdio, com comprometimento contrátil mediado por citocinas produzidas pelo processo inflamatório local. Esse quadro inflamatório agudo pode evoluir, na fase tardia, para fibrose extensa, o que pode causar dilatação e disfunção ventricular.

A miocardite aguda é suspeitada quando existe a presença dos seguintes critérios: ^283^

- Síndrome clínica com IC aguda, dor torácica do tipo angina ou miopericardite com menos de 3 meses de duração;
- Elevação inexplicada na dosagem de troponina sérica;
- Alterações eletrocardiográficas sugestivas de isquemia miocárdica;
- Anormalidades contráteis globais ou segmentares e/ou derrame pericárdico na ecocardiografia.
- RMN com alterações características no sinal tecidual em T2, ou imagens ponderadas em T1, e presença de realce tardio com gadolínio.

Em relação à participação em programas de RCV de pacientes com miocardites após a resolução da fase aguda, o assunto é muito pouco estudado e não existem estudos científicos sobre a segurança e eficácia da intervenção. No entanto, relatos de casos de RCV nesse perfil de pacientes têm demonstrado benefícios na qualidade de vida e na aptidão física, especialmente quando há comprometimento funcional, mesmo após a melhora do quadro agudo e otimização do tratamento medicamentoso. ^288 - 290^

Antes de iniciar a prática de exercícios, os pacientes com quadro prévio de miocardite devem ser submetidos a ecocardiograma, Holter de 24 horas e teste de exercício em um período não inferior a 3 a 6 meses após a doença aguda. ^269 , 283^ Depois dessa avaliação, casos selecionados podem iniciar exercícios moderados na RCV, visando os benefícios gerais obtidos com os pacientes com IC.

No âmbito esportivo, é razoável que atletas retornem a sua rotina normal de treinamento apenas se houver: retorno da função sistólica a valores normais; marcadores de necrose miocárdica e inflamação dentro dos valores normais e ausência de arritmias clinicamente significativas no Holter e no teste de exercício. Ressalta-se que o significado clínico da permanência de realce tardio na ressonância de pacientes pós-miocardite, com resolução do quadro, ainda é desconhecido. Sendo assim, parece razoável que aqueles com pequenas áreas de realce e sem arritmias significativas no Holter e no exercício possam retornar à atividade esportiva, mantendo acompanhamento clínico. ^269^

Em casos crônicos, em que a disfunção ventricular persiste ao longo do seguimento, o paciente deve seguir as recomendações gerais para a RCV descritas para a IC crônica (ver Tabela 6 ).

### 6.6.3. Outras Miocardiopatias

***6.6.3.1. Cardiomiopatia Arritmogênica do Ventrículo Direito***

A cardiomiopatia arritmogênica do ventrículo direito (CAVD) é uma doença hereditária que está associada à MS em jovens e atletas. Patologicamente, ocorre perda de miócitos, com substituição fibroadiposa, principalmente no miocárdio do ventrículo direito, embora o acometimento isolado do ventrículo esquerdo ou o biventricular também possam ocorrer. ^291^

Há evidências, em modelo experimental animal, de que o exercício aumenta a penetrância e o risco de arritmias em portadores de mutações tradicionais da CAVD. ^292^ Em indivíduos com genótipos positivos, o aumento do risco de arritmias com o exercício também já foi confirmado. Os eventos de taquiarritmias ventriculares e MS geralmente ocorrem durante o esforço, incluindo esportes e exercícios de *endurance* , com um aumento no risco de taquicardia, fibrilação ventricular e IC. ^293^

Já foi demonstrado que indivíduos com CAVD envolvidos em esportes competitivos apresentaram maior ocorrência de taquiarritmias ventriculares e MS, além de início mais precoce dos sintomas, comparados com aqueles que participaram apenas de atividade física leve e sedentários. ^270^ A redução da intensidade do exercício foi associada à diminuição substancial do risco de taquiarritmias ventriculares ou morte, principalmente nos pacientes sem mutação desmossomal detectada e com CDI para prevenção primária. ^294^ Portanto, a evidência científica sugere que a participação em esportes e exercício intenso está associada ao início precoce dos sintomas e maior risco de arritmias ventriculares e eventos maiores em pacientes com CAVD. Sendo assim, devem ser desqualificados para a prática esportiva. ^269 , 276^

Em relação à participação em programas de RCV, não há dados científicos que indiquem ou que sugiram benefícios dos exercícios físicos para os pacientes com CAVD. Por outro lado, mantê-los sedentários, contribuindo para a baixa aptidão física, também pode não ser apropriado, visto que existe associação geral de baixa aptidão física com mortalidade. ^14 , 21^

Em um pequeno estudo observacional com pacientes com CAVD, não foi observada diferença na taxa de mortalidade entre os indivíduos inativos e os que realizaram apenas atividades físicas recreacionais. ^270^ Desse modo, pode-se supor que a participação dos pacientes em um programa de RCV supervisionado, com exercícios de leve a moderada intensidade, pode não ser deletéria. Dependendo de outras características clínicas dos indivíduos, como presença de fatores de risco cardiovasculares, os exercícios físicos poderiam ser prescritos para o controle dessas condições.

Portanto, a inclusão de um paciente com CAVD em programas de RCV somente deve ser realizada após a avaliação médica pré-participação e o rigoroso ponderamento entre os riscos e benefícios dos exercícios físicos. Devem ser discutidas as opções com o paciente, expondo a ausência de benefícios comprovados *versus* riscos potenciais do sedentarismo e baixa aptidão física. Cabe ao paciente escolher a opção, de acordo com suas preferências pessoais.

No contexto da RCV, extrapolando os achados em atletas, sugere-se também restrição a maiores intensidades de treinamento. Os pacientes com CAVD poderiam realizar exercícios físicos supervisionados de leve a moderada intensidade.

***6.6.3.2. Miocardiopatia Não Compactada***

A miocardiopatia não compactada (MNC) é uma doença cardíaca que ocorre devido à interrupção embrionária da compactação miocárdica. Caracteriza-se por espessamento segmentar das paredes do ventrículo esquerdo, consistindo em duas camadas: uma epicárdica compactada e uma endocárdica com marcadas trabeculações e recessos intratrabeculares profundos, onde os espaços são preenchidos pelo fluxo sanguíneo. ^295 , 296^

Sua incidência e prevalência são incertas, segundo alguns registros ecocardiográficos, em torno de 0,02 a 0,05%. ^297^ Clinicamente, pode ser assintomática ou cursar com sintomas de IC, arritmias ventriculares e/ou atriais, pré-excitação, eventos tromboembólicos ou MS. Não existem critérios universalmente aceitos para o diagnóstico morfológico. Contudo, a relação entre miocárdio não compactado/compactado superior a 2,1:1 no final da sístole ao ecocardiograma ou 2,3:1 no final da sístole na RMN tem sido o critério proposto mais aceito atualmente. ^298^

Ainda não está estabelecido como o treinamento físico pode influenciar a MNC ou a frequência de aparecimento da morfologia de não compactação na população. ^299 , 300^ Em estudos recentes, atletas revelaram alta prevalência de aumento da trabeculação ventricular, quando comparados a um grupo controle (18,3 *versus* 7%). Acredita-se que o aumento da trabeculação ventricular ou a existência de critérios ecocardiográficos isolados para miocardiopatias tenham, provavelmente, pequena significância e possam ser parte do espectro do coração de atleta. ^300 , 301^ Portanto, nem todos os atletas com não compactação ventricular isolada têm o diagnóstico de MNC. Diante disso, existe a necessidade de se considerarem parâmetros funcionais, como a FEVE, para decisão de conduta. ^301^

Não existem, até o momento, evidências de estudos com RCV ou treinamento na MNC. Sendo assim, pacientes que apresentem disfunção ventricular esquerda devem seguir as mesmas recomendações de exercício para aqueles com IC crônica (ver Tabela 6 ).

## 6.7. Valvopatias

Pacientes com valvopatias representam um grupo bastante heterogêneo e podem ter grande variabilidade quanto a faixa etária, etiologia, valvas acometidas e gravidade das lesões, seja por estenose, insuficiência ou lesões mistas. Entretanto, a maioria das valvopatias tem características em comum, que são as manifestações clínicas induzidas pelo esforço físico, como dor torácica, dispneia e/ou limitações funcionais. A gravidade desses sintomas em pacientes com valvopatias graves pode ser utilizada como um dos critérios para a indicação de intervenção cirúrgica ou percutânea. Além disso, a identificação de redução da aptidão aeróbica, documentada pelo TCPE ou pelo TE, também se constitui em critério utilizado para definição de indicação de intervenções. ^302 - 304^

Um dos problemas no seguimento clínico dos pacientes com valvopatias é que a doença tem longa evolução. Os sintomas e limitações funcionais podem ter lenta instalação e progressão, o que pode levar o paciente a, espontaneamente, reduzir a sua prática de atividade física, em virtude de sintomatologia aos esforços. Com isso, o sedentarismo pode contribuir para a redução da aptidão física aeróbica e amplificar sintomas.

Desse modo, dúvidas podem surgir na condução clínica sobre a necessidade de intervenções quando o paciente realizar um TE ou TCPE, como: a identificação de eventuais reduções da aptidão física poderia ser decorrente da evolução da valvopatia, do sedentarismo ou de ambas as situações? Nesse contexto, a prática regular de exercícios físicos e a consequente manutenção ou até melhora da aptidão física são importantes para dirimir dúvidas no seguimento de pacientes valvopatas.

A participação de pacientes valvopatas em programas de RCV ainda é objeto de estudo sobre o significado em termos de custo-efetividade. ^305^ Porém, o aumento da capacidade funcional dos indivíduos encaminhados à RCV tem sido consistentemente encontrado, ^306 , 307^ justificando o encaminhamento a programas embasados em exercícios físicos (nível de evidência C).

A atuação da reabilitação no cenário da valvopatia pode ser subdividida em duas fases: pré e pós-intervenção, seja esta cirúrgica ou percutânea.

### 6.7.1. Fase Pré-intervenção

Pacientes com valvopatias moderadas a graves, na fase pré-intervenção, são menos comuns em programas de RCV. O treinamento é realizado principalmente em casos assintomáticos, nos quais ainda não existe indicação de correção da valvopatia.

A RCV pode ser útil por manter o paciente fisicamente ativo durante a espera pela futura intervenção. Afinal, o sedentarismo pode deteriorar sua capacidade funcional e, com isso, aumentar o risco de complicações no pós-operatório, principalmente quando a intervenção é realizada em idosos com múltiplas comorbidades e fragilidade. ^308 - 310^

Além disso, o monitoramento realizado durante as sessões supervisionadas da RCV pode ser útil para observar mudanças na sintomatologia e aptidão física, as quais podem indicar uma possível progressão da valvopatia e sugerir a necessidade de reavaliações médicas.

### 6.7.2. Fase Pós-intervenção

Pacientes na fase pós-intervenção são mais comuns em programas de RCV, nos quais o exercício estruturado e sob supervisão é útil para a observação do comportamento hemodinâmico da nova condição valvar. A obtenção de informações relativas às respostas ao exercício físico pode ajudar o médico assistente em relação à necessidade de ajustes farmacológicos e/ou revisões da função valvar. Além disso, a prática supervisionada dos exercícios confere maior segurança ao paciente para retornar às suas atividades diárias, de lazer e esporte.

Apesar de não existir nenhum prazo de tempo consensualmente definido para o encaminhamento à RCV no cenário da valvopatia, quanto mais precocemente o paciente iniciar os exercícios, menores serão os prejuízos relacionados à inatividade física. ^305 - 307 , 310^ A troca de informações entre o médico assistente e o da reabilitação configura-se como a melhor estratégia para definição do momento mais propício para o encaminhamento, e a avaliação pré-participação tem um papel fundamental na consolidação dessa decisão compartilhada.

### 6.7.3. Avaliação Pré-participação

A avaliação pré-participação sempre terá como pilares básicos e fundamentais a anamnese, o exame físico e a avaliação dos exames complementares. A história clínica deve contemplar: tempo de internação; complicações relacionadas ao procedimento, como derrame pleural, pericárdico, mediastinite e infecções; tipo e tamanho da prótese utilizada; técnica cirúrgica; e se houve CRVM associada, além de outras informações clínicas que possam ser pertinentes, relativas a outras comorbidades.

No exame físico, as auscultas cardíaca e pulmonar são importantes. Além disso, atenção deve ser dada à cicatriz cirúrgica, com verificação de sinais de inflamação e infecção, instabilidade do esterno e dor ou desconforto à palpação. Caso tenha sido realizada revascularização concomitante, deve-se observar a região da safenectomia e/ou da retirada da artéria radial. Em casos de procedimentos percutâneos, verifica-se a via de acesso em busca de sinais de complicações vasculares periféricas.

A busca por anemia no exame físico e na avaliação dos exames complementares é importante nos pacientes pós-intervenção, pois é uma situação frequente e pode impactar negativamente na capacidade funcional. ^311^ A avaliação laboratorial da coagulação é relevante nos pacientes que receberam próteses valvares e iniciaram uso de anticoagulantes. A adequação do nível de anticoagulação é importante na prevenção de complicações.

O ECG de repouso deve ser realizado para avaliar a ocorrência de arritmias e distúrbios do ritmo e da condução. O exame mais comumente utilizado na avaliação das valvopatias é o ecocardiograma com Doppler, que possibilita a avaliação da função ventricular e de diâmetros cavitários, a mensuração de gradientes transvalvares, a estimativa da pressão sistólica da artéria pulmonar e as medidas dos fluxos, o que dá uma visão ampla do funcionamento do aparelho valvar e da função cardíaca em repouso. Sendo assim, o ecocardiograma deve ser realizado antes do início da RCV para avaliar o risco de complicações nos exercícios. ^312^

A avaliação da capacidade funcional pelo TCPE ou TE é de importância ímpar na análise complementar. ^313 - 316^ Esses exames, principalmente o TCPE, fornecem informações relativas à aptidão aeróbica e à repercussão hemodinâmica da lesão valvar, que pode estar subestimada pela avaliação em repouso. Além disso, identifica parâmetros que são utilizados para guiar a prescrição dos limites de intensidade e restrições causadas pela valvopatia. Na indisponibilidade do TCPE ou TE, a utilização de outros testes funcionais, como o teste de caminhada de 6 minutos e o teste de degraus, deve ser considerada. ^317 - 320^

É importante ressaltar que o TCPE ou TE em pacientes com lesões estenóticas configuram situações de maior risco. Por esta razão, devem ser realizados somente por médicos com experiência nesse tipo de avaliação e em serviço com retaguarda de segurança. ^321^

Além da indicação dos testes funcionais na avaliação pré-participação da RCV, sua utilização também é adequada para esclarecer dúvidas em relação à sintomatologia de pacientes valvopatas na fase pré-intervenção. A associação com o ecocardiograma ajuda a avaliar a resposta em esforço físico do gradiente transvalvar e da pressão sistólica de artéria pulmonar, principalmente quando há discrepância entre os achados do ecocardiograma em repouso e os sinais e sintomas clínicos. ^304 , 322 , 323^

Outra questão relevante é a avaliação de pacientes idosos, que frequentemente são acometidos pelas doenças valvares e apresentam uma alta prevalência de fatores de risco e comorbidades. ^324^ Em virtude do elevado risco cirúrgico, tais pacientes têm sido submetidos a procedimentos percutâneos das valvas aórtica ^325^ e mitral. ^326^ Nessa situação, a RCV pode ser considerada antes da intervenção, com o objetivo de diminuir as taxas de complicações, o tempo de internação, a mortalidade e a morbidade relacionadas à síndrome da fragilidade. ^327^ Após a realização da intervenção, a RCV permite monitorar e otimizar os resultados do procedimento em todos os seus aspectos. ^328 - 331^

A utilização de instrumentos de avaliação da síndrome de fragilidade ainda é objeto de discussão na literatura, sem um consenso de qual o melhor protocolo para avaliar os resultados da RCV. A avaliação deve incluir testes objetivos e instrumentos de abordagem do risco em vários domínios: mobilidade, massa e força musculares, independência nas atividades da vida diária, função cognitiva, nutrição, ansiedade e depressão. ^304 , 308 , 332^

### 6.7.4. Particularidades na Prescrição e no Acompanhamento dos Exercícios Físicos

Nesta seção, são abordadas as orientações e recomendações para exercícios em pacientes com lesões valvares de grau moderado ou grave, visto que não há restrição para a prática nos casos de lesões leves. A participação em esportes competitivos deve observar as publicações específicas sobre o assunto. ^276 , 333 , 334^ A evidência científica é escassa quanto ao impacto do exercício físico regular na progressão da doença valvar e de suas complicações. Portanto, as recomendações são fundamentadas em opiniões de especialistas (nível de evidência C).

Agudamente, o exercício provoca um aumento do tônus adrenérgico e da carga hemodinâmica imposta ao sistema cardiovascular, o que causa preocupação com relação aos potenciais efeitos deletérios cardiovasculares nos pacientes com valvopatias, tais como progressão de aortopatias, deterioração funcional, hipertensão pulmonar, remodelamento cardíaco, isquemia miocárdica e arritmias.

Pacientes com doenças valvares que iniciarão um programa de RCV devem ser submetidos a um teste de esforço para avaliação e prescrição dos exercícios. A Tabela 9 resume as recomendações para pacientes assintomáticos, sem intervenção prévia, com doenças valvares moderadas ou importantes. Em geral, o treinamento será realizado com combinação dos exercícios aeróbicos e resistidos. Quando não houver restrições, as recomendações para prescrição de exercício serão as mesmas utilizadas para indivíduos sem cardiopatia.

**Tabela 9:** – Exercícios físicos em indivíduos assintomáticos com valvopatias

Valvopatia	Exercício aeróbico	Exercício resistido
Insuficiência aórtica	**Moderada ou importante** (função ventricular normal; DSVE < 50 mm em homens ou < 40 mm em mulheres; boa capacidade funcional) **Sem restrições**	**Moderada ou importante Evitar alta intensidade**
Estenose aórtica	**Moderada ou importante** (função ventricular normal; boa capacidade funcional; ausência de isquemia miocárdica, arritmia ventricular complexa ou resposta em platô/queda da PAS) **Evitar alta intensidade**	**Moderada** Evitar alta intesidade **Importante** Limitada a baixa intensidade para manutenção das atividades cotidianas
Insuficiência mitral	**Moderada ou importante** (boa função ventricular; DDVE < 60 mm; PSAP < 30 mmHg) **Sem restrições**	**Moderada ou importante** Evitar alta intensidade
Estenose mitral	**Moderada ou importante** (boa capacidade funcional) **Evitar alta intensidade**	**Moderada ou importante** (boa capacidade funcional) **Evitar alta intensidade**

*DDVE: diâmetro diastólico do ventrículo esquerdo; DSVE: diâmetro sistólico final do ventrículo esquerdo; PAS: pressão arterial sistólica; PSAP: pressão sistólica da artéria pulmonar.*

Para pacientes sintomáticos, sem indicação de correção cirúrgica ou que não apresentam as características descritas na Tabela 9 , a intensidade do exercício deve ser limitada, conforme a ocorrência de anormalidades observadas no teste de esforço, pois se assume que insultos repetidos nessa intensidade poderiam aumentar o risco dos exercícios e induzir, a longo prazo, potenciais efeitos deletérios na valvopatia. A prescrição de exercício deve ser limitada à intensidade de esforço equivalente a 10 bpm abaixo da FC em que ocorreu a anormalidade no teste de esforço. As cargas e a percepção subjetiva de esforço podem ser utilizadas em situações em que a FC não é um bom parâmetro de controle, como na fibrilação atrial ou em ritmos controlados pelo marcapasso artificial. ( Tabela 10 ).

**Tabela 10:** – Alterações no teste de exercício que indicam limites para prescrição de intensidade do treinamento em valvopatas

Alterações induzidas pelo esforço	Detalhamento
Sinais e sintomas	Início de angina, equivalente anginoso ou outros sinais/sintomas indicativos de intolerância ao exercício
Pressão arterial	Início do comportamento em platô ou queda da PAS; ou PAS > 220 mmHg; ou PAD > 115 mmHg
Segmento ST	Início do infradesnivelamento (horizontal ou descendente) do segmento ST superior a 1 mm
Função ventricular	Evidência de queda da função ventricular no esforço ou início de anormalidade moderada a importante da mobilidade parietal do ventrículo esquerdo
Pulso O _2_ no TCPE	Sinais de platô precoce ou queda no esforço, apesar do aumento da carga
Arritmias	Bloqueio AV de graus 2 e 3, fibrilação atrial, taquicardia supraventricular ou arritmia ventricular complexa

*TCPE: teste cardiopulmonar de exercício; PAS: pressão arterial sistólica; PAD: pressão arterial diastólica, AV: atrioventricular.*

Em pacientes que foram submetidos à correção cirúrgica da valvopatia, os limites de intensidade da prescrição dependerão da doença de base, do resultado do procedimento, da presença de lesões residuais, da função ventricular e da resposta ao teste de exercício. Sendo assim, cada caso deve ser analisado individualmente e os limites definidos pela avaliação médica e pelos resultados dos exames complementares realizados.

## 6.8. Portadores de Marcapasso cardíaco ou Cardioversor Desfibrilador Implantável

Esta seção se destina às particularidades sobre os dispositivos implantáveis: marcapasso cardíaco (MP) e cardioversor desfibrilador implantável (CDI). O MP é indicado em função de anormalidades elétricas, que podem ser isolada (doença do nó sinusal, bloqueio atrioventricular (AV) de grau avançado) ou associada a cardiopatias estruturais. O CDI é indicado para a prevenção primária ou secundária de MS, em pacientes com doenças elétricas e/ou cardiopatias graves. Dependendo da cardiopatia presente, devem ser consideradas as recomendações sobre a RCV abordadas anteriormente.

Uma das preocupações nos exercícios físicos em portadores de MP ou CDI é relativa ao risco de complicações com o dispositivo, especialmente em atividades com chances de colisão corporal. Nos portadores de CDI, há o receio de choques, o que pode levar a modificações comportamentais nos pacientes, com redução da atividade física diária e participação em exercícios de moderada intensidade. ^335 , 336^ Os profissionais de saúde também compartilham desses receios, ^337^ o que pode reduzir as orientações para a prática de exercícios. Porém, tem sido demonstrado que o exercício físico é seguro e não está associado ao aumento do risco de choques ou de outros eventos adversos. ^338 - 342^ Além disso, não têm sido observadas complicações relativas ao CDI, mesmo em atletas competitivos. ^343 , 344^

Entretanto, é de fundamental importância, para a adequada liberação dos exercícios, que se conheçam o motivo do implante e os parâmetros de programação do dispositivo, que deverão ser investigados na avaliação pré-participação.

### 6.8.1. Benefícios Terapêuticos dos Exercícios Físicos

Uma meta-análise ^342^ que englobou 14 estudos com um total de 2.681 pacientes portadores de CDI comprovou efeito benéfico do exercício físico na capacidade funcional desses indivíduos, com um aumento médio de VO _2_ de 2,4 ml.kg ^- 1^ .min ^- 1^ . Em outra meta-análise, com cinco estudos randomizados e um não randomizado em pacientes com IC e CDI, ^341^ o resultado na capacidade física foi semelhante, com aumento no VO _2_ pico de 1,98 ml.kg ^- 1^ .min ^- 1^ em relação ao grupo controle.

Quanto às terapias por CDI e treinamento físico, uma das meta-análises não encontrou diferenças significativas. O percentual de choques associados aos exercício variou de 0 a 20% entre os estudos, com uma média de 2,2%, similar ao percentual de choques em um período de seguimento não relacionado ao exercício. ^342^ Sendo assim, apesar dos receios previamente descritos, o treinamento físico não se associou a aumento dos choques pelo CDI e se mostrou seguro.

Outra meta-análise relatou menor probabilidade de choques ao longo do seguimento nos pacientes participantes da RCV em relação aos controles, corroborando o resultado anterior de um estudo observacional, que relatou maior incidência de choques pelo CDI em pacientes que não participavam de programas de RCV. ^341 - 345^

Uma das possíveis explicações para a menor incidência de arritmias e choques nos pacientes em RCV seria a melhora da capacidade física, pois já foi previamente documentado que maior aptidão física está associada a menor incidência de arritmias. ^16 , 17 , 346^ Além disso, os exercícios poderiam reduzir a arritmogenicidade miocárdica, em função do remodelamento e da menor excitabilidade simpática. ^347^

Em um estudo nacional com 10 anos de acompanhamento, que contou com 150 pacientes com CDI em programa de RCV, submetidos a TCPE ou TE para prescrição do treinamento, ocorreram apenas três eventos de choques apropriados, o que reforça a segurança das avaliações e da RCV nesses indivíduos. ^348^

### 6.8.2. Quando Indicar Reabilitação Cardiovascular

O exercício físico pode e deve ser indicado desde que a condição clínica do paciente seja estável e o tratamento clínico, otimizado. Além dos benefícios potenciais na cardiopatia de base, a RCV contribui para o aumento da capacidade física e pode atuar na redução das arritmias e nos choques pelo CDI ( Tabela 11 ).

**Tabela 11:** – Indicações de exercícios físicos e outros tratamentos em pacientes com cardiodesfibrilador implantável

Indicação	Recomendação	Nível de evidência
Exercícios físicos para aumento de capacidade física em pacientes estáveis e portadores de CDI ^341,342^	I	A
Exercícios físicos para possível redução da probabilidade de choques em pacientes portadores de CDI ^341^	IIa	B
Utilização de eletroestimulação neuromuscular em portadores de dispositivos com sensores bipolares, quando realizada em musculaturas distantes do implante ^349^	IIb	B

*CDI: cardioversor desfibrilador implantável. Considerar as recomendações em relação à cardiopatia estrutural, se presente.*

### 6.8.3. Avaliação Pré-participação

Nos portadores de dispositivos implantáveis há necessidade de conhecer o motivo do implante, a função ventricular, a presença de arritmias e, principalmente, os parâmetros de ajuste do dispositivo. Dentre os ajustes do MP, é importante saber o modo de programação, os limites programados de FC, o tipo e a adaptação do sensor de frequência. Nos pacientes com CDI, é fundamental obter informações relativas às FC programadas para terapias de choque ou *burst* .

Além da avaliação clínica habitual, a avaliação ao esforço é de suma importância, sendo ideal a realização do TCPE ou TE para determinação da capacidade funcional e análise do comportamento do dispositivo em esforço. Entretanto, a impossibilidade de realização desses exames não deverá ser um fator limitante para a prática de exercícios. Nesses casos, o monitoramento das sessões poderá dar indícios da necessidade de adequações na programação do dispositivo, geralmente em relação aos ajustes da FC máxima programada e da resposta do sensor.

Durante as sessões da RCV, poderá ser utilizado o monitoramento eletrocardiográfico, que pode ser feito com o uso de sistemas de telemetria. Dispositivos para controle da FC, como os cardiofrequencímetros, também podem ser usados para monitoramento durante as sessões de RCV desses pacientes. ^350^ Em virtude das alterações do traçado, causadas pelo comando artificial, pode haver erro na determinação automática da FC, tanto pela análise eletrocardiográfica quanto pelos frequencímetros. Sendo assim, é importante atenção a esses possíveis erros, com verificação manual, se necessária.

### 6.8.4. Particularidades na Prescrição e no Acompanhamento dos Exercícios Físicos

Na prescrição e definição de limites de intensidade para o treinamento físico aeróbico, deve-se ter conhecimento da programação do CDI e limitar a intensidade a 10 a 20 bpm abaixo da FC programada para a terapêutica (choque ou *burst* ). Esse cuidado é especialmente importante em indivíduos jovens, que podem ter FC elevada no treinamento. Em pacientes mais idosos, com IC e uso de altas doses de betabloqueadores, a FC pico observada no TE ou TCPE costuma estar abaixo da FC de terapia do CDI.

Os pacientes portadores de MP podem ter diferentes respostas cronotrópicas, observadas no TCPE ou TE, o que irá impactar na prescrição dos exercícios aeróbicos. Adicionalmente, o ritmo próprio do indivíduo, o tipo de MP e a presença de sensor influenciarão a resposta da FC ao esforço e, consequentemente, a prescrição. ^351^

A seguir estão listados quatro tipos de possíveis respostas do MP ao esforço:

**1) Resposta cronotrópica sinusal normal ou deprimida. MP sem atuação (inibido).** A resposta cronotrópica em esforço é mediada pelo ritmo sinusal e pode estar normal ou deprimida (por doença do nó sinusal e/ou efeito medicamentoso). A condução ventricular ocorre pela via própria, e o MP não atua no esforço. Em alguns casos, ele pode atuar em repouso e em cargas iniciais, com comando atrial e/ou ventricular. Porém, no esforço, o MP se inibe, predominando as respostas sinusais e a condução ventricular pela via própria. Nesse tipo de resposta ao esforço, a prescrição de intensidade segue as rotinas habituais e não é influenciada pela presença do MP.

**2) Resposta cronotrópica sinusal normal ou deprimida. MP com comando ventricular no esforço.** A resposta cronotrópica em esforço é mediada pelo ritmo sinusal, que é percebida pelo MP com subsequente comando ventricular, de modo sincronizado e de acordo com os intervalos AV programados. Nesse caso, se a programação do limite máximo de resposta de FC do MP for adequada à resposta sinusal do paciente, não haverá problema para a prescrição de intensidade por FC, pois o ventrículo estará pareado com a atividade sinusal. Porém, se a FC máxima programada do MP for inferior à resposta sinusal do paciente, em moderada a alta carga haverá uma perda do pareamento da atividade ventricular com a sinusal. Então, o MP bloqueará alguns estímulos sinusais por meio de um *Wenckebach* mediado pelo dispositivo ou eletrônico, ^352^ de modo a manter a FC ventricular dentro do limite programado, havendo um platô na resposta cronotrópica ao esforço. Nessas situações, a perda do pareamento do ritmo sinusal com a frequência ventricular limitará a utilização da FC para controle de intensidade. A prescrição deverá ser feita por cargas relativas e/ou sensação subjetiva de esforço.

No caso do *Wenckebach* eletrônico, é necessária a extrema atenção no TCPE ou TE, após iniciar a sua ocorrência. É fundamental ter a informação precisa de qual é a FC atrial em que o MP inicia o bloqueio 2:1, pois nela o comando ventricular será na proporção 2:1, podendo ocorrer queda súbita da FC em esforço, a qual pode ser sintomática, por redução abrupta do débito cardíaco. Sendo assim, a menos que as FC programadas de *Wenckebach* eletrônico e de bloqueio 2:1 sejam muito distantes, a FC do *Wenckebach* eletrônico poderá tornar-se um limite para o TCPE ou TE e para a prescrição dos exercícios.

Nesses casos, deve-se considerar e discutir com o médico assistente a reprogramação do MP, para melhor pareamento com a resposta sinusal do paciente. Outra opção, a depender do quadro clínico, é a otimização de medicações cronotrópicas negativas, como os betabloqueadores. Com isso, a menor resposta sinusal poderá evitar a ocorrência descrita.

**3) Resposta cronotrópica mediada pelo MP e fixa, com ausência de sensor.** Alguns pacientes podem não ter atividade sinusal, como na fibrilação atrial. Nesses casos, em indivíduos com bloqueio AV completo, haverá total dependência de comando ventricular pelo MP. Se não houver sensor, ou se este estiver desativado, haverá ausência de resposta cronotrópica ao esforço e o MP terá FC fixa. Esse tipo de MP ou programação é muito raro atualmente e tal resposta limita completamente a utilização da FC na prescrição, que deve se basear na determinação da intensidade por cargas e/ou pela sensação subjetiva do esforço.

**4) Resposta cronotrópica mediada pelo MP com presença de sensor.** Nos pacientes com fibrilação atrial e bloqueio AV, conforme descrito anteriormente, mas com o sensor do MP presente e ativado, haverá dependência do comando ventricular, mas a ativação do sensor no esforço conduzirá a uma resposta cronotrópica mediada pelo MP. Em pacientes com ritmo sinusal, mas com grande déficit cronotrópico, por doença do nó sinusal e/ou efeito medicamentoso, poderá ocorrer resposta cronotrópica ao esforço também mediada pelo sensor do MP, com comando atrial seguido ou não de comando ventricular.

A velocidade e magnitude da resposta do sensor ao esforço são programáveis, com possibilidades de ajustes do limiar da ativação do sensor, da velocidade de incremento da FC ao esforço e sua redução na recuperação, bem como do limite máximo da FC do sensor. Na realização do TCPE ou TE, poderá ser verificada a adequação da resposta, com identificação de possíveis necessidades de reprogramações do MP, que devem ser discutidas com o médico assistente.

Nesses casos, como a resposta cronotrópica será mediada artificialmente pelo dispositivo, a prescrição de intensidade de exercícios por FC poderá ser imprecisa. Sendo assim, a utilização das cargas relativas e/ou a percepção do esforço serão preferenciais.

Dispositivos com sensores do tipo acelerômetro e detecção do movimento axial, que são os mais usuais, têm boa resposta ao esforço na esteira, caminhada ou corrida. Porém, na bicicleta estacionária não há movimento vertical e o sensor não ativa ou é pouco ativado. Com isso, há menor resposta cronotrópica no ergômetro, que pode variar de acordo com a resposta individual do paciente.

### 6.8.5. Treinamento Resistido

A prática de exercícios resistidos é importante na RCV em diversas cardiopatias. Entretanto, após o implante do dispositivo, alguns cuidados são necessários até a completa cicatrização, a fim de evitar lesão vascular, deslocamento do gerador e fratura de eletrodos. Recomenda-se, por exemplo, cautela ao executar exercícios com pesos e elevação excessiva dos MMSS durante as primeiras seis semanas após o implante. Além disso, movimentos repetitivos e intensos com o membro relacionado ao implante do marcapasso devem ser evitados.

Entretanto, tais orientações estão mais ligadas a pacientes envolvidos com esportes, sendo improváveis no caso de exercícios realizados em centros de RCV. Em um estudo com mobilização precoce e supervisionada da cintura escapular após o implante imediato de MP, não foram observadas complicações ao dispositivo. ^353^

### 6.8.6. Estimulação Elétrica Neuromuscular

A utilização da eletroestimulação neuromuscular (ENM) em pacientes com IC tem sido difundida, principalmente naqueles impossibilitados de praticar exercícios físicos pela gravidade clínica. A ENM pode melhorar a capacidade aeróbica, a força muscular e a área transversa da musculatura do quadríceps, demonstrando ser uma efetiva opção de exercício passivo nessa população. ^354 - 356^ Entretanto, também é crescente o uso de dispositivos eletrônicos nesses pacientes (CDI, MP e ressincronizadores), o que causa preocupação quanto ao uso da ENM, pela possibilidade de interferência eletromagnética.

Uma revisão sistemática ^349^ demonstrou que a ENM na musculatura de quadríceps parece ser segura e viável em pacientes com IC e CDI com sensores bipolares. Entretanto, a própria revisão ressalta que o número de estudos e pacientes avaliados é muito pequeno para conclusões mais abrangentes e conclui que o uso pode ser feito se forem satisfeitas as seguintes condições:

1. Se os riscos individuais (dependência do MP, IC aguda, angina instável, arritmia ventricular nos últimos 3 meses) tiverem sido excluídos antes de iniciar a ENM.
2. Se o uso da ENM for realizado apenas nos músculos de quadríceps e glúteos.
3. Se o tratamento for regularmente supervisionado por um médico e o dispositivo for avaliado após o uso da ENM.

Portanto, no momento, apesar de parecer ser segura a utilização da ENM em portadores de dispositivos com sensores bipolares, quando realizada em musculaturas distantes do implante, ainda há necessidade de estudos com maior número de pacientes, para que haja possibilidade de uso amplo sem avaliação detalhada do dispositivo.

## 6.9. Doença Arterial Obstrutiva Periférica

O AVC tem sido justamente tratado como doença grave e de grande repercussão em saúde pública. Porém, outras doenças arteriais periféricas também são muito prevalentes e apresentam grande morbimortalidade, embora não tenham sido devidamente abordadas, prejudicando a prevenção, o diagnóstico e o efetivo tratamento. ^357 , 358^ Nesse contexto, destaca-se a doença arterial obstrutiva periférica (DAOP) de MMII, que, em seu estágio mais grave, a isquemia crítica, apresenta elevado risco de eventos cardiovasculares, amputação de MMII e morte. A isquemia crítica dos MMII, com o crescimento de fatores de risco, tais como idade, diabetes e tabagismo, tem aumentado a sua prevalência e acomete, atualmente, cerca de 2 milhões de indivíduos somente nos EUA. ^359^

A presença de DAOP é suspeitada quando há dor em MMII ao esforço, sem aparente etiologia ortopédica, e o índice tornozelo-braquial (ITB) é menor que 0,90 em repouso. ^360 , 361^ O ITB tem sido recomendado como recurso diagnóstico a ser usado anteriormente à realização de métodos de imagem. ^362^ Testes funcionais em esforço podem ser necessários para auxiliar no diagnóstico, especialmente quando o ITB for maior que 0,91, e também para classificação funcional e prescrição de exercícios na RCV.

A caminhada pode ser avaliada por meio de testes de campo, que possibilitam o diagnóstico de claudicação intermitente, com determinação das distâncias percorridas para o início da sintomatologia (claudicação inicial) e para o surgimento da total limitação funcional (claudicação absoluta).

Em esteira, tem sido proposta a utilização diagnóstica de teste funcional em esforço, com medida do ITB em repouso e após exercício. A presença de DAOP é sugerida quando ocorre redução do ITB pós-exercício superior a 20% em relação ao repouso, ou diminuição da pressão pós-exercício maior que 30 mmHg em relação ao repouso. ^363^ Outro estudo relatou notas de corte menores, sendo sugerida DAOP quando há redução do ITB pós-exercício acima de 18,5% e diminuição da pressão pós-exercício maior que 15 mmHg. ^364^

Considerando o risco cardiovascular global desses pacientes, o tratamento clínico otimizado deve sempre ser instituído. Além disso, a interrupção do tabagismo e a terapia farmacológica com estatinas e antiagregantes plaquetários devem ser consideradas, bem como o adequado controle glicêmico e pressórico. Em relação ao uso do Cilostazol, não há um consenso nas diretrizes de sociedades médicas. ^362 , 363^

Em pacientes sintomáticos, os exercícios têm potencial para influenciar na morbimortalidade, com redução dos sintomas, melhora da qualidade de vida e aumento da distância máxima caminhada ( Tabela 12 ). ^365^ As atividades físicas realizadas sob supervisão direta têm se mostrado mais efetivas do que sem supervisão. ^366^

**Tabela 12:** – Tratamento da doença arterial obstrutiva periférica de membros inferiores

Indicação	Recomendação	Nível de evidência
Exercício físico supervisionado para melhora funcional, da qualidade de vida e redução da claudicação ^365,369,375,376^	I	A
Exercício físico domiciliar ou outras modalidades de treinamento para melhora funcional ^366,370,371^	IIa	A
Em pacientes sintomáticos, um programa de exercícios físicos supervisionados deve ser discutido como opção de tratamento antes da revascularização ^375,376^	I	B

Em 14 ensaios clínicos (1.002 participantes), com intervenção entre 6 semanas e 12 meses, a caminhada livre de dor aumentou cerca de 180 metros a mais no treinamento sob supervisão direta, quando comparado ao treinamento sob supervisão indireta. O treinamento físico tem se mostrado seguro. Na maioria dos estudos são realizados exercícios de caminhadas, com indução do sintoma de claudicação, em programas com duração mínima de 3 meses e, pelo menos, três sessões semanais. ^367^

Nos paciente com DAOP, o treinamento sob supervisão direta tem sido superior em termos de custo-efetividade, ^368^ embora aquele sob supervisão indireta (RCV domiciliar) tenha se mostrado uma boa alternativa, com efeitos positivos sobre a qualidade de vida e a tolerância à caminhada, sendo significativamente superior à mera recomendação para caminhar. ^369 , 370^

Quando a caminhada não puder ser realizada, outros tipos de atividades, como ciclismo, exercícios resistidos e ergômetro de MMSS, têm se mostrado efetivos. ^371^ Cabe ainda ressaltar que exercícios físicos não podem ser realizados por pacientes com isquemia crítica, mas devem ser considerados o mais breve possível após tratamento intervencionista com sucesso. ^371 - 373^

Uma revisão sistemática de 12 ensaios clínicos, com um total de 1.548 pacientes, comparando os claudicantes em tratamento farmacológico (em treinamento físico), os com intervenção endovascular e os com cirurgia aberta, mostrou que todas as alternativas proporcionaram aumento da distância caminhada, redução de sintomas e melhora da qualidade de vida. ^374^ A intervenção endovascular e a cirurgia aberta têm comprovadamente se mostrado eficazes para o alívio de sintomas, aumento da distância caminhada e melhora da qualidade de vida. Além disso, estão indicadas quando, após a realização do tratamento clínico pleno ou otimizado (exercícios físicos e tratamento farmacológico otimizado), persistirem sintomas graves que influenciem negativamente na vida diária.

Em um ensaio clínico randomizado com 111 pacientes com DAOP aortoilíaca e seguimento de 6 meses, foi evidenciado que o aumento do tempo de exercício no TE incremental foi maior no grupo que realizou exercícios supervisionados do que no que realizou revascularização com *stent* . ^375^ Entretanto, após 18 meses de seguimento, os benefícios funcionais e na qualidade de vida foram equivalentes nos grupos com treinamento ou com revascularização e, em ambos os casos, foram superiores aos do grupo que realizou somente tratamento farmacológico. ^376^

Vários ensaios clínicos compararam a eficácia e efetividade do exercício físico supervisionado, a angioplastia e o tratamento clínico otimizado, utilizando uma infinidade de desenhos diferentes. A maioria dos ensaios consistia em dois braços de tratamento. Revisões sistemáticas já citadas sugeriram que o exercício físico supervisionado pode ser superior ao tratamento farmacológico otimizado ou à angioplastia. Essas meta-análises, no entanto, incluíram estudos com comparações diretas entre dois braços de tratamento específicos (p. ex., angioplastia *vers* us treinamento físico supervisionado) ou utilizaram uma abordagem que não permitia a inclusão e a comparação direta de todos os tratamentos disponíveis da claudicação intermitente. ^377^

Por esses motivos, uma recente meta-análise buscou estabelecer todas as comparações entre os tratamentos disponíveis, no intuito de verificar a melhor conduta no manejo do paciente com DAOP sintomática. Foram incluídos 2.983 pacientes com claudicação intermitente (média de idade de 68 anos e 54,5% de homens). As comparações foram realizadas entre tratamento clínico otimizado (n = 688), treinamento físico supervisionado (n = 1.189), angioplastia (n = 511) e angioplastia mais treinamento físico supervisionado (n = 395). O seguimento médio foi de 12 meses. Comparados ao tratamento medicamentoso otimizado isoladamente, a angioplastia e o treinamento físico supervisionado superaram todas as outras estratégias terapêuticas, havendo ganho de distância máxima de caminhada de 290 m (IC 95%: 180 a 390 m; p < 0,001) ou ganho proporcional de 141% (IC 95%: 86,85 a 188,3%; p < 0,001), com período médio de acompanhamento de 12 meses. ^378^

O treinamento físico supervisionado isoladamente e a angioplastia associada ao treinamento físico supervisionado novamente superaram as demais modalidades de tratamento, com ganho de distância máxima de caminhada de 110 m (IC 95%: 16 a 200 m; p < 0,001) ou incremento proporcional de 66% (IC 95%: 9,66 a 121%; p < 0,001). O treinamento físico supervisionado, com aumento de distância máxima de caminhada de 180 m (IC 95%: 130 a 230 m) e ganho proporcional de 87% (IC 95%: 63 a 111%) foi superior à angioplastia isolada, mas inferior ao treinamento físico supervisionado associado à angioplastia, no quesito distância máxima de caminhada. ^378^

Esses estudos de revisão têm implicações importantes para a prática clínica. Isso porque todos os pacientes com claudicação intermitente devem receber tratamento clínico otimizado, tendo em vista as evidências que demonstram redução de eventos cardiovasculares futuros e melhora de desfechos relacionados aos membros. ^379 , 380^ Nesse contexto, o treinamento físico supervisionado e a angioplastia são fundamentais para melhorar a distância de caminhada e a qualidade de vida. Essa recente meta-análise citada sugere fortemente que o treinamento físico supervisionado, associado à angioplastia, deve fazer parte do tratamento de primeira linha, sempre no contexto da terapia medicamentosa otimizada. A oferta de angioplastia sem treinamento físico otimizado deve ser evitada sempre que possível. ^378^ Porém, frequentemente, os centros de tratamento da DAOP oferecem primeiramente a angioplastia, devido à carência de centros voltados ao treinamento físico supervisionado. Não se pode negligenciar que o treinamento físico supervisionado enfrenta resistência por parte do próprio paciente, provocando pouca adesão ao tratamento, o que justifica, em parte, a conduta majoritária pelo tratamento percutâneo. ^381^

Entretanto, esses estudos recentes, que investigaram as modalidades de tratamento da DAOP sintomática com treinamento físico isolado ou associado à angioplastia, têm demonstrando os benefícios da combinação dos tratamentos, o que pode aumentar a probabilidade de que a RCV se torne cada vez mais difundida e acessível. ^378 , 382^

Sendo assim, além do tratamento clínico otimizado, a angioplastia combinada ao treinamento físico supervisionado parece ser a estratégia ideal para tratamento inicial dos pacientes com claudicação intermitente, tanto para melhorar a máxima distância de caminhada, como para a qualidade de vida. No entanto, os dados dessas últimas revisões são incapazes de afirmar se primeiramente deve ser proposto o treinamento físico supervisionado e, posteriormente, a angioplastia, ou vice-versa.
