# Supplementary material for: Diretriz Brasileira de Reabilitação Cardiovascular – 2020
Source: Arq Bras Cardiol. 2020 May 22;114(5):943–87. [Article in Portuguese] doi: 10.36660/abc.20200407 (PMC8387006; doi:10.36660/abc.20200407)
Supplement: Translation

# Translation: Brazilian Cardiovascular Rehabilitation Guideline – 2020

**Authors:** Tales de Carvalho, Mauricio Milani, Almir Sergio Ferraz, Anderson Donelli da Silveira, Artur Haddad Herdy, Carlos Alberto Cordeiro Hossri, Christina Grüne Souza e Silva, Claudio Gil Soares de Araújo, Eneas Antonio Rocco, José Antonio Caldas Teixeira, Luciana Oliveira Cascaes Dourado, Luciana Diniz Nagem Janot de Matos, Luiz Gustavo Marin Emed, Luiz Eduardo Fonteles Ritt, Marconi Gomes da Silva, Mauro Augusto dos Santos, Miguel Morita Fernandes da Silva, Odilon Gariglio Alvarenga de Freitas, Pablo Marino Corrêa Nascimento, Ricardo Stein, Romeu Sergio Meneghelo, Salvador Manoel Serra

**Affiliations:** 1 Brazil Clínica de Prevenção e Reabilitação Cardiosport, Florianópolis, SC – Brazil; 2 Brazil Universidade do Estado de Santa Catarina (Udesc), Florianópolis, SC – Brazil; 3 Brazil Fitcordis Medicina do Exercício, Brasília, DF – Brazil; 4 Brazil Instituto Dante Pazzanese de Cardiologia, São Paulo, SP – Brazil; 5 Brazil Programa de Pós-Graduação em Cardiologia e Ciências Cardiovasculares da Universidade Federal do Rio Grande do Sul (UFRGS), Porto Alegre, RS – Brazil; 6 Brazil Hospital de Clínicas de Porto Alegre, Universidade Federal do Rio Grande do Sul (HCPA/UFRGS), Porto Alegre, RS – Brazil; 7 Brazil Vitta Centro de Bem Estar Físico, Porto Alegre, RS – Brazil; 8 Brazil Instituto de Cardiologia de Santa Catarina, Florianópolis, SC – Brazil; 9 Brazil Unisul: Universidade do Sul de Santa Catarina (UNISUL), Florianópolis, SC – Brazil; 10 Brazil Hospital do Coração (Hcor), São Paulo, SP – Brazil; 11 Brazil Clínica de Medicina do Exercício, Clinimex, Rio de Janeiro, RJ – Brazil; 12 Brazil Hospital Samaritano Paulista, São Paulo, SP – Brazil; 13 Brazil Universidade Federal Fluminense (UFF), Rio de Janeiro, RJ – Brazil; 14 Brazil Instituto do Coração (Incor) do Hospital das Clínicas da Faculdade de Medicina da Universidade de São Paulo (HC-FMUSP), Rio de Janeiro, RJ – Brazil; 15 Brazil Hospital Israelita Albert Einstein, São Paulo, SP – Brazil; 16 Brazil Hospital Cardiológico Costantini, Curitiba, PR – Brazil; 17 Brazil Hospital Cárdio Pulmonar, Salvador, BA – Brazil; 18 Brazil Escola Bahiana de Medicina e Saúde Pública, Salvador, BA – Brazil; 19 Brazil SPORTIF – Clínica do Exercício do Esporte, Belo Horizonte, MG – Brazil; 20 Brazil ACE Cardiologia do Exercício, Rio de Janeiro, RJ – Brazil; 21 Brazil Instituto Nacional de Cardiologia, Rio de Janeiro, RJ – Brazil; 22 Brazil Universidade Federal do Paraná, Curitiba, PR – Brazil; 23 Brazil Minascor Centro Médico, Belo Horizonte, MG – Brazil; 24 Brazil Instituto Estadual de Cardiologia Aloysio de Castro (IECAC), Rio de Janeiro, RJ – Brazil

**Development:** *Department of Exercise Testing, Sports Exercise, Nuclear Cardiology, and Cardiovascular Rehabilitation of the Brazilian Society of Cardiology (SBC)*

**Norms and Guidelines Council:** *Brivaldo Markman Filho, Antonio Carlos Sobral Sousa, Aurora Felice Castro Issa, Bruno Ramos Nascimento, Harry Correa Filho, Marcelo Luiz Campos Vieira*

**Norms and Guidelines Coordinator:** *Brivaldo Markman Filho*

**Guideline Coordinators:** *Tales de Carvalho e Mauricio Milani*

**Table:** 

Declaration of potential conflict of interests of authors/collaborators of the Brazilian Cardiovascular Rehabilitation Guideline – 2020 If, within the last 3 years, the author/collaborator of the guideline:							

Names of guideline collaborators	Participated in clinical and/or experimental studies sponsored by pharmaceutical or equipment companies related to this guideline	Spoke at events or activities sponsored by industry related to this guideline	Was (is) a member of a board of advisors or a board of directors of a pharmaceutical or equipment industry	Participated in normative committees of scientific research sponsored by industry	Received personal or institutional funding from industry	Wrote scientific papers in journals sponsored by industry	Owns stocks in industry
Almir Sergio Ferraz	Novartis, Amgen, Sanofi	No	No	No	No	Boehringer	No
Anderson Donelli da Silveira	No	No	No	No	No	No	No
Artur Haddad Herdy	No	No	No	No	No	No	No
Carlos Alberto Cordeiro Hossri	No	No	No	No	No	No	No
Christina Grüne Souza e Silva	No	No	No	No	No	No	No
Claudio Gil Soares de Araújo	No	No	No	No	Inbramed	No	No
Eneas Antonio Rocco	No	No	No	No	No	No	No
José Antonio Caldas Teixeira	No	No	No	No	No	No	No
Luciana Diniz Nagem Janot de Matos	No	No	No	No	No	No	No
Luciana Oliveira Cascaes Dourado	No	No	No	No	No	No	No
Luiz Gustavo Marin Emed	No	No	No	No	No	No	No
Luiz Eduardo Fonteles Ritt	No	No	No	No	No	No	No
Marconi Gomes da Silva	No	No	No	No	No	No	No
Mauricio Milani	No	No	No	No	No	No	No
Mauro Augusto dos Santos	No	No	No	No	No	No	No
Miguel Morita Fernandes da Silva	No	No	No	No	Novartis	No	No
Odilon Gariglio Alvarenga de Freitas	No	No	No	No	No	No	No
Pablo Marino Corrêa Nascimento	No	No	No	No	No	No	No
Ricardo Stein	No	No	No	No	No	No	No
Romeu Sergio Meneghelo	No	No	No	No	No	No	No
Salvador Manoel Serra	No	No	No	No	No	No	No
Tales de Carvalho	No	No	No	No	No	No	No

Content

1. Introduction 946

1.1. Strengths (Grades) of Recommendation 947

1.2. Levels of Evidence 947

2. Structure of a Cardiovascular Rehabilitation Program 947

2.1. Staffing and Individual Responsibilities 947

**2.1.1. Primary Physician 947**

**2.1.2. Lead Physician of Cardiovascular Rehabilitation Program 947**

**2.1.3. Other Health Care Workers 947**

**2.1.4. Physical Therapists and Physical Educators 947**

**2.1.5. Nurses 947**

2.2. Physical Infrastructure of a Rehabilitation Service 948

**2.2.1. General Aspects 948**

**2.2.2. Fitness and Exercise Equipment 948**

***2.2.2.1. Aerobic Exercise* 948**

***2.2.2.2. Strength Training* 948**

***2.2.2.3. Other Exercise Modalities* 948**

**2.2.3. Monitoring 948**

**2.2.4. Safety 948**

3. Phases of Cardiovascular Rehabilitation and Risk Stratification 949

3.1. High Clinical Risk 949

3.2. Intermediate Clinical Risk 950

3.3. Low Clinical Risk 951

4. Cost-Effectiveness of Cardiovascular Rehabilitation 951

5. Home-Based Cardiovascular Rehabilitation 952

6. Integration of Cardiovascular Rehabilitation into Optimized Clinical Care of Cardiovascular Diseases 953

6.1. General Guidance for Increasing Physical Activity and Engagement in Physical Exercise 953

6.2. Hypertension 955

**6.2.1. Therapeutic Benefits of Physical Exercise 955**

**6.2.2. Indications for Physical Exercise in Hypertension 955**

**6.2.3. Pre-Exercise Evaluation 956**

**6.2.4. Special Considerations for the Prescription and Follow-Up of Physical Exercise Programs 956**

6.3. Stable Coronary Artery Disease after an Acute Event or Revascularization 957

**6.3.1. Therapeutic Benefits of Physical Exercise 957**

**6.3.2. When Is Rehabilitation Indicated? 957**

**6.3.3. Pre-Exercise Evaluation and Exercise Prescription 958**

**6.3.4. Special Considerations for the Prescription and Follow-Up of Physical Exercise Programs 959**

***6.3.4.1. Refractory Angina* 959**

***6.3.4.2. Exercise Training with Myocardial Ischemia* 959**

***6.3.4.3. Drug Adjustments in Response to Physical Exercise* 959**

6.4. Heart Failure 960

**6.4.1. Pre-Exercise Evaluation and Exercise Prescription 960**

**6.4.2. Final Considerations on Heart Failure 962**

6.5. Heart Transplantation 962

**6.5.1. Benefits of Physical Exercise 962**

**6.5.2. Pre-Exercise Evaluation and Unique Features 963**

**6.5.3. Exercise Prescription 963**

**6.5.4. Home-Based Cardiovascular Rehabilitation 963**

**6.5.5. Recommendations 964**

6.6. Cardiomyopathies 964

**6.6.1. Hypertrophic Cardiomyopathy 964**

***6.6.1.1. Therapeutic Benefits of Physical Exercise* 965**

***6.6.1.2. When Is Physical Exercise Indicated?* 966**

***6.6.1.3. Pre-Exercise Evaluation* 966**

***6.6.1.4. Unique Features in the Prescription and Follow-Up of Physical Exercise Programs* 966**

**6.6.2. Myocarditis 967**

**6.6.3. Other Cardiomyopathies 967**

***6.6.3.1. Arrhythmogenic Right Ventricular Cardiomyopathy* 967**

***6.6.3.2. Noncompaction Cardiomyopathy* 968**

6.7. Valvular Heart Disease 968

**6.7.1. Pre-Intervention Phase 968**

**6.7.2. Post-Intervention Phase 968**

**6.7.3. Pre-Exercise Evaluation 969**

**6.7.4. Special Considerations for the Prescription and Follow-Up of Physical Exercise Programs 969**

6.8. Patients with Artificial Pacemakers or Implantable Cardioverter-Defibrillators 970

**6.8.1. Therapeutic Benefits of Physical Exercise 970**

**6.8.2. When Is Cardiovascular Rehabilitation Indicated? 971**

**6.8.3. Pre-Exercise Evaluation 971**

**6.8.4. Special Considerations for the Prescription and Follow-Up of Physical Exercise Programs 971**

**6.8.5. Resistance Training 973**

**6.8.6. Neuromuscular Electrical Stimulation 973**

6.9. Peripheral Arterial Occlusive Disease 973

References 975

## 1. Introduction

It is common sense—and has been scientifically proven – that physical activity helps to preserve and restore the health of both body and mind. The favorable effects of cardiovascular rehabilitation (CVR), with an emphasis on physical exercise, include significant reductions in cardiovascular and overall morbidity and mortality, ^1^ reductions in hospitalization rate, ^1 , 2^ and significant gains in quality of life, ^1 , 2^ as consistently documented in the literature, including in meta-analyses of randomized clinical trials. These effects justify the consensual, emphatic recommendation of CVR by major medical societies worldwide. ^3 - 6^

Sedentary behavior, which is highly prevalent in Brazil and elsewhere, is strongly associated with cardiovascular disease (CVD) and early mortality. ^7 , 8^ Conversely, higher levels of physical activity are positively associated with better quality of life and longer life expectancy. ^9 - 13^ In addition, there is a strong, inverse association of the various components of physical fitness with all-cause mortality and with occurrence of adverse cardiovascular events: the lower the level of physical fitness, the higher the mortality rate. ^14 - 21^

Therefore, the main objective of CVR, with an emphasis on physical exercise, is to improve the various components of physical fitness, both aerobic and non-aerobic (muscle strength/endurance, flexibility, balance). This requires a combination of different exercise modalities and types of training. In this view, beyond rehabilitation, CVR aims to provide the highest achievable level of physical fitness – in order to reduce the risk of further cardiovascular events – and to promote all of the other benefits derived from regular physical exercise. ^14 - 21^

However, despite its documented benefits and excellent cost-effectiveness, ^22 , 23^ CVR is underutilized worldwide. In Brazil, considering the size of the country and the diversity of its population, several barriers limit access to RCV, ^24 , 25^ such as a scarcity of structured services, limited urban mobility, and high rates of violence in cities. ^26 , 27^ Within this context, so-called home-based cardiovascular rehabilitation (HBCR) programs, in which most sessions take place in the patient’s home under indirect supervision, can supplement or serve as an alternative to traditional programs in which training sessions are always carried out under direct supervision.

As in previous documents on this topic published by the Brazilian Society of Cardiology, ^6 , 28 - 31^ this guideline will exclusively address interventions based on physical exercise. The strength (or grade) of recommendation will always be proportional to the level of evidence available, as explained below.

### 1.1. Strengths (Grades) of Recommendation

- **Grade I**: there is conclusive evidence, or, failing that, a consensus that the procedure is safe and useful/effective;
- **Grade II**: there is conflicting evidence and/or divergent opinions on the safety and utility/effectiveness of the procedure:

**– Grade IIA**: weight of the evidence/opinion is in favor of the procedure. Most experts approve;
**– Grade IIB**: safety and utility/effectiveness are less well established, with no predominance of opinions in favor.

- **Grade III**: there is evidence and/or expert consensus that the procedure is not useful/effective and, in some cases, can even be harmful.

### 1.2. Levels of Evidence

- **Level A**: data obtained from multiple, large, concordant randomized studies and/or robust meta-analyses of randomized clinical studies;
- **Level B**: data obtained from a less robust meta-analysis, based on a single randomized trial or on non-randomized (observational) studies;
- **Level C**: data obtained from consensus expert opinions.

## 2. Structure of a Cardiovascular Rehabilitation Program

### 2.1. Staffing and Individual Responsibilities

The makeup of CVR teams must be adjusted to its objectives, target audience, and availability of human and material resources, taking into account regional characteristics, the modality of rehabilitation (direct or indirect supervision), and the site or setting (hospital, outpatient clinic, etc.). A multidisciplinary CVR team is usually composed of physicians, physical educators, physical therapists, and nurses, but may also include other professionals, such as dietitians, psychologists, and social workers. ^31 , 32^

#### 2.1.1. Primary Physician

CVR is an integral part of the optimized clinical treatment of patients with stable CVD. Thus, it is essential that the CVR team and the patient’s primary physician work in an integrated manner. When referring a patient for rehabilitation, primary clinicians must be aware of its indications and potential benefits and should carry out the necessary pre-exercise evaluation. As this integration will involve frequent progress reports, potential needs for adjustment of drug therapy, awareness of complications and intercurrent events, etc., it is very important that mechanisms be established to facilitate communication between the patient’s primary physician and the CVR team. ^31^

#### 2.1.2. Lead Physician of Cardiovascular Rehabilitation Program

The lead physician coordinates all medical activities. In Brazil, this role usually falls to the general coordinator of the CVR program. He or she must have in-depth subject knowledge of CVR and be trained to act in cardiovascular emergencies. ^6 , 32 - 34^

Some of the main activities of this position include:

- Perform pre-exercise evaluation, including cardiopulmonary exercise testing as needed, to provide inputs for the initial prescription of CVR training sessions. ^31^
- Train the CVR team to identify high-risk situations and provide appropriate care in emergencies.
- Establish restrictions and set limits for the exercise prescription.
- Lead and interact with other team members, to optimize the quality and safety of exercise prescription.
- Schedule follow-up evaluations, always in coordination with the patient’s primary physician.

#### 2.1.3. Other Health Care Workers

Like physicians, the other members of the team, when carrying out their respective duties, must follow the program’s rules and guidelines as well as the formal recommendations of their respective professional associations. ^31^

#### 2.1.4. Physical Therapists and Physical Educators

Physical therapists and physical educators are directly involved in the prescription and supervision of physical exercise, within the targets and limits defined by the physician as a result of the pre-exercise evaluation and follow-ups. They must have specific knowledge of CVD and exercise physiology and training in basic life support, including the use of an automated external defibrillator. Such training must be periodically refreshed to ensure continued competence. In addition to their direct role during CVR sessions, these professionals can provide patient guidance and contribute to other lifestyle measures aimed at adopting healthy habits.

#### 2.1.5. Nurses

In a CVR program, nurses and other nursing professionals can assist in clinical evaluation and obtain and provide information on the patient’s medical status, including through contact with family members. Nurses can also be in charge of blood glucose measurements and blood pressure (BP) checks before and/or during exercise sessions. In case of complications or intercurrent events, nurses can provide direct care, assist the physician and administer medications. Nurses must, of course, also be trained in basic life support, including the use of an automated external defibrillator.

2.2. Physical Infrastructure of a Rehabilitation Service

#### 2.2.1. General Aspects

A CVR program can be run out of various types of facilities, depending on its objectives and on the available resources. Most often, CVR programs are carried out indoors, in air-conditioned environments, although exercise sessions can be held in outdoor venues such as running tracks, courts, gymnasiums, parks, or public recreation spaces. ^29^

Indoor venues should be adequately sized and appointed, with dimensions and characteristics varying according to local resources and service capacity. The space should be large enough for patients to exercise in, ideally with a ceiling height not less than 2.5 meters. It should also be properly lit, well ventilated, and climate-controlled so as to maintain a temperature of 22–25°C and a relative humidity of 40–65% during sessions. The exercise area per se, not considering changing rooms, restrooms, and the reception area or waiting room, varies greatly – from 20 m ^2^ to a several hundred square meters. Proper changing rooms and restrooms are essential. To minimize the risk of accidental falls, slip-resistant flooring is mandatory. ^29^

#### 2.2.2. Fitness and Exercise Equipment

***2.2.2.1. Aerobic Exercise***

The most commonly used aerobic exercise equipment are treadmills and stationary bicycles (cycle ergometers), but upper-body ergometers, rowing machines, cross-country ski machines, and elliptical trainers can be used, among countless others. ^29^

Treadmills must be electric, with a nominal capacity of at least 100 kg body weight, front and side supports for the hands, and a safety key. They must also allow individualized adjustments of speed and slope over a wide range. Cycle ergometers can be mechanical or electromagnetically braked. There are specific models for the upper body, and even some models which allow all four limbs to be exercised simultaneously. Conventional (lower body) models may be upright or recumbent. Ideally, the cycle ergometer should provide a readout of cadence or speed and, most importantly, power (in watts). Some cycle ergometers allow the user to program the intensity directly in watts, so that the resistance of the pedals increases when the cadence decreases and vice versa.

Rowing machines, ski machines, and elliptical trainers can be particularly useful for patients with a lower degree of functional limitation or who have had previous experience with such equipment. These devices provide the advantage of allowing simultaneous exercise of the upper and lower limbs.

***2.2.2.2. Strength Training***

Several types of equipment can be used for strength training. Bodyweight exercises, which require no equipment at all, are often sufficient in the most debilitated patients. One representative and functional example is rising from a seated position, which requires only a chair or bench.

Ropes or straps, firmly attached to the ceiling or high on a wall, can also be used to facilitate a wide range and variety of bodyweight exercises. Free weights, dumbbells, or ankle weights are often adopted in CVR programs, as they allow patients to execute a wide range of movements and provide appropriate stimulation of different muscle groups. Specific devices consisting of weights connected to cables and pulleys, known as cable machines or stack machines, can also be used. Other useful equipment includes workout bars, weighted exercise balls (also known as medicine balls), stability balls (also known as Swiss balls), and elastic bands or tubes (also known as exercise bands or resistance bands) with varying degrees of resistance. ^29^

During all exercises, attention must be paid to proper posture and execution of the prescribed movements, so as to prevent musculoskeletal injuries. Attention when handling exercise equipment is also important to prevent accidents and potential injury.

***2.2.2.3. Other Exercise Modalities***

With a view to overall health, considering heart disease and associated conditions, patients may benefit from or even require other types of exercise, such as isometric handgrip training, inspiratory muscle training, and exercises designed to improve balance and flexibility.

#### 2.2.3. Monitoring

Several modalities are available for patient monitoring, including heart monitors, mobile applications for monitoring heart rate (HR), glucometers, pulse oximeters, and conventional devices such as sphygmomanometers and stethoscopes. Depending on the clinical complexity and the risk of unfavorable cardiovascular events, continuous electrocardiographic monitoring (at rest or during exercise) may be convenient. This can be achieved by conventional ECG (connected directly to the patient) or by telemetry systems. Rapid access to monitoring equipment is of fundamental importance for proper detection and subsequent management of potential cardiovascular events.

#### 2.2.4. Safety

Although serious cardiovascular events – such as cardiac arrest, which, in most adults, results from ventricular fibrillation or pulseless ventricular tachycardia – are extremely uncommon during CVR, it is essential that all programs have a plan in place to respond appropriately to these events if they do occur. Therefore, a defibrillator (whether manual or automated) is mandatory safety equipment. Other basic and advanced life support supplies must also be available, such as laryngoscopes, orotracheal tubes of various sizes, masks, a bag-valve-mask manual resuscitator, and supplemental oxygen.

For more detailed guidance on techniques, equipment, and recommended drugs, readers are advised to check subject-specific guidelines. ^35 , 36^

## 3. Phases of Cardiovascular Rehabilitation and Risk Stratification

Traditionally, CVR is divided into time-bound phases, with phase 1 occurring in hospital and phases 2 to 4 in the outpatient setting. In the early days of CVR, phase 1 was intended for recovery after acute myocardial infarction (AMI) or coronary artery bypass surgery (CABG). Subsequently, in the context of what is now known as cardiopulmonary and metabolic rehabilitation, phase 1 was expanded to include hospitalized patients who underwent percutaneous coronary intervention (PCI), valve replacement or repair surgery, procedures for congenital heart disease, and heart transplantation (HTx), in addition to those with heart failure (HF), coronary artery disease (CAD), diabetes, hypertension, and chronic lung and kidney disease (once clinically stable). Therefore, CVR should begin immediately once the patient is considered clinically stable as a result of clinical and/or interventional treatment. ^31^

In phase 1 of CVR, the aim is for the patient to be discharged from the hospital in the best possible physical and psychological condition and with guidance to pursue a healthy lifestyle, especially with regard to physical exercise. A combination of low-intensity physical exercise, techniques for stress control, and education on risk factors and heart disease is recommended. The team must be composed of at least one physician, one physical therapist, and one nurse. All should be trained specifically in CVR, but full-time dedication to the rehabilitation program is not required; team members are free to perform other duties in the hospital. ^31^

Upon discharge from hospital, patients must be referred to the outpatient phases of CVR. Phase 2 begins immediately after hospital discharge and lasts, on average, 3 months. Phase 3 usually lasts 3 to 6 months, and phase 4 is quite prolonged. In all phases, the goal is to obtain progressive benefits from RCV or at least maintain any gains obtained.

A strict division of CVR into time-bound phases may fail to take into account that some very symptomatic, debilitated patients with severe heart disease will remain in long-term “phase 2” rehabilitation, as they continue to require direct supervision of physical exercise, while other low-risk patients may be fit for phase 3 or even phase 4 programs straight away, and are thus candidates for home-based CVR (in which most sessions take place under indirect, remote supervision). ^31^

Therefore, stratification of clinical risk is recommended to enable a more rational use of CVR programs, with individualized targeting of phases and modalities. In this context, high-risk patients, those with less physical capacity, and those most symptomatic should participate in supervised sessions indefinitely, while those at lower risk, with greater physical capacity and fewer or less severe symptoms can engage in a wider range of more intense exercises without direct supervision ( Figure 1 ).

**Figure 1:** – General characteristics of patients undergoing outpatient cardiovascular rehabilitation stratified by clinical risk. MET: metabolic equivalent; VO 2 : oxygen consumption

Stratification of clinical risk as high, intermediate, or low is based on existing recommendations, ^4 , 28 , 37^ while the cut-off points for this classification are based on expert opinion (level C evidence), and can thus be modified according to the experience of the CVR team and the discretion and judgment of the clinician in response to the pre-exercise evaluation and subsequent evaluations ( Table 1 ).

**Table 1:** – Clinical risk stratification of patients undergoing outpatient cardiovascular rehabilitation

Risk	High	Intermediate	Low

Features			
Cardiovascular event, cardiovascular intervention, or clinical decompensation	Less than 8 to 12 weeks	12 weeks or longer	6 months or longer
Physical functioning	TMET: < 5 MET CPET: Weber C/D or VO _2_ peak < 60% of predicted	TMET: 5–7 MET CPET: Weber B or VO _2_ peak 60–85% of predicted	TMET: > 7 MET CPET: Weber A or VO _2_ peak > 85% of predicted
Signs and symptoms of myocardial ischemia (ischemic threshold)	At low loads TMET: at < 6 MET CPET: at < 15 ml.kg ^-1^ .min ^-1^	TMET: at > 6 MET CPET: at > 15 ml.kg ^-1^ .min ^-1^	Absent
Symptoms	HF: NYHA III and IV Angina: CCS III and IV	HF: NYHA I and II Angina: CCS I and II	Absent
Other clinical features:	Dialytic CKD; oxygen desaturation on exertion; complex ventricular arrhythmia.	At clinician’s discretion and judgment during pre-exercise evaluation	At clinician’s discretion and judgment during pre-exercise evaluation

*CCS: Canadian Cardiovascular Society; CKD: chronic kidney disease; CPET: cardiopulmonary exercise test; HF: heart failure; MET: metabolic equivalent; NYHA: New York Heart Association functional class; TMET: treadmill exercise test; VO _2_: oxygen consumption.*

### 3.1. High Clinical Risk

The duration of CVR can vary according to the patient’s clinical picture and progress of physical training. The initial classification, maintenance, and reclassification of risk profile must be based on the pre-exercise evaluation and on subsequent revaluations, carried out by the physician and other CVR team members. This evalution may vary according to the logistics, infrastructure and experience of each CVR service, but must at least consist of a clinical history, physical examination, resting electrocardiogram (ECG), and cardiopulmonary exercise test (CPET) or treadmill exercise test (TMET).

High-risk patients will often need immediate or short-term medical attention during CVR (hospital readmission, intervention, or adjustment of drug therapy). Therefore, they require closer monitoring by the team, which must be able to identify signs and symptoms of high-risk events and act immediately if such an event arises, providing basic and advanced life support, including with use of a manual or automated external defibrillator as necessary. It is preferable that this equipment be present in the room at all times. The medical team must be readily available on site to attend to the patient in the event of any serious complications.

It should be noted that the best way to prevent cardiovascular events during a rehabilitation program, and especially after events and interventions, is to conduct systematic pre-exercise evaluation and revaluations.

The exercise program must be individualized in terms of intensity, duration, frequency, training modality, and progression, according to the functional testing performed at the start of the program and subsequently. Proper measurement of HR and BP at rest and during exercise are mandatory; measurement of oxygen saturation, capillary blood glucose, and electrocardiographic monitoring should also be available.

The rehabilitation program should also include an educational program aimed at lifestyle modification, with an emphasis on dietary re-education and strategies for smoking cessation, if necessary. It is essential that patients acquire knowledge about their illness and learn to self-monitor, both while exercising and in terms of red-flag signs and symptoms which may signal unstable or high-risk clinical situations.

The clinical characteristics of patients who would initially be classified as high clinical risk (presence of at least one such feature) are:

- Hospitalization due to recent (< 8–12 weeks) cardiovascular events: AMI or unstable angina; surgical or percutaneous revascularization; complex arrhythmias; cardiac arrest; acute decompensated HF.
- Chronic heart disease with or without recent cardiovascular events and/or interventions but with significant functional changes on physical exertion, i.e.:

– Low functional capacity on TMET (< 5 MET) or CPET (Weber C/D classification or VO _2_ peak < 60% of predicted for age and sex).
– Signs and symptoms of myocardial ischemia at low loads (< 6 MET or at a VO _2_ of < 15 ml.kg ^-1^ .min ^-1^ ).
– Exacerbated symptoms (HF with NYHA functional class III or IV, or angina with CCS functional class III and IV).

- Other clinical characteristics of patients at increased risk during physical exercise include dialytic chronic kidney disease (CKD), oxygen desaturation on exertion, and complex ventricular arrhythmias at rest or exertion.

Considering that high-risk patients often need frequent readjustment of drug therapy and reassessment and occasionally need advanced intervention (revascularization or other procedures), constant communication between the CVR team and the patient’s primary physician(s) is essential. It is also important to note that some patients who experience intercurrent events during exercise or unfavorable findings on follow-up evaluations may remain in the high-risk classification (i.e., requiring direct supervision of physical exercise) indefinitely.

### 3.2. Intermediate Clinical Risk

Patients may have completed previous stages of CVR and been reclassified; may be classified directly into this category despite no previous engagement in exercise; or may have been referred from other exercise programs. The duration of CVR under this classification can also be variable, depending on the clinical status and progress achieved with physical training as demonstrated in follow-up evaluations.

Exercises should be supervised by a physical therapist or physical educator, and the service should (ideally) rely on the coordination of a physician with experience in CVR. Devices for measurement of HR and BP at rest and during exercise are recommended; measurement of oxygen saturation, capillary blood glucose, and electrocardiographic monitoring should also be available as necessary.

If there is no on-site physician, one must be readily on call. Basic life support material must be available on site and all team members must be trained in cardiopulmonary resuscitation, including use of an automated external defibrillator, which must also be present on site.

It is essential that pre-exercise evaluation be carried out by the CVR team, with appropriate risk stratification. Regular medical follow-up and revaluations as necessary must be carried out to ensure the safety of the exercise regimen.

The clinical characteristics of patients who would initially be classified as intermediate risk (presence of at least one such feature) are:

- Longer than 12 weeks since the latest cardiovascular event or intervention and currently stable clinical condition.
- Chronic heart disease with some loss of function on exertion:

– Moderate functional capacity on TMET (5–7 MET) or CPET (Weber B classification or VO _2_ peak 60–85% of predicted for age and sex).
– Signs and symptoms of myocardial ischemia at loads > 6 MET or at a VO _2_ > 15 ml.kg ^- 1^ .min ^- 1^ .
– Mild to moderate symptoms (HF with NYHA functional class I or II, or angina with CCS functional class I and II).

- Any other clinical features judged by the physician responsible for pre-exercise evalution to pose intermediate cardiovascular risk on exertion.

The main objective of CVR in patients with this risk profile remains the improvement of physical fitness, both aerobic and non-aerobic (muscle strength, flexibility, balance, body composition), as well as superior disease control. The need to promote wellness and improved quality of life, in addition to other procedures that contribute to reducing the risk of clinical complications, such as strategies for smoking cessation, dietary reeducation, and weight control, should all be considered. An emphasis on maintaining and adhering to prescribed drug therapy is also essential to preventing the progression or instability of CVD. Acquisition of knowledge about the disease itself, allowing for better self-management, increases the accuracy in identifying signs and symptoms of disease progression or red flags of unstable clinical situations, which may require interruption of the exercise program and medical revaluation.

Patients in this category, after an initial period of guidance and knowledge acquisition about physical exercise and self-monitoring, may be able to adapt to home-based CVR, in which physical exercise is performed under the indirect supervision of team members. Such supervision, as well as adjustments to the exercise prescription or patient education to clarify any questions, should take place during periodic face-to-face or remote follow-up sessions.

### 3.3. Low Clinical Risk

Patients may have completed previous stages of CVR and been reclassified; may be classified directly into this category despite no previous engagement in CVR; or may have been referred from other physical exercise programs. Training for these patients is a long-term endeavor, aimed at maintaining overall health and achieving the greatest possible gains in physical fitness, with the objective of reaching the highest attainable standard of health.

Depending on availability and individual preferences, exercises can be carried out under direct (face-to-face) or remote supervision. However, given their lower clinical risk and reduced need for supervision, patients at this stage are a perfect fit for home-based rehabilitation models, so that the CVR team can devote on-site resources to patients at higher clinical risk.

Patients must nevertheless undergo periodic reevaluation by their primary physician and by the CVR team, including CPET or TMET. In principle, the time between follow-up revaluations should not exceed 12 months. The purpose of follow-up is to readjust the exercise prescription and identify any deterioration of the underlying disease or red flags for clinical decompensation or cardiovascular events, thus allowing preemptive adjustment of drug therapy and/or surgical or percutaneous intervention as needed.

Patients receiving home-based programs should be periodically reevaluated and receive guidance on exercise. These occasions are advised to serve as opportunities for participation in supervised exercise sessions, especially for less-experienced patients, as well as for readjustment of the exercise prescription as needed and to answer any questions. Periodic remote assessment by the CVR team (through virtual and/or telephone contact), at least once every 6 months, is recommended to encourage continued adherence to the physical exercise program.

The clinical characteristics of stage 4 patients are (all of the following must be present):

- Longer than 6 months since the latest cardiovascular event or intervention and currently stable clinical condition.
- Chronic heart disease with little or no loss of function on exertion.
- Patients in this classification usually exhibit the following:

– Good functional capacity on TMET (> 7 MET) or CPET (Weber A classification or VO _2_ peak > 85% of predicted for age and sex).
– No signs or symptoms of myocardial ischemia and no unusual symptoms on physical exertion.

## 4. Cost-Effectiveness of Cardiovascular Rehabilitation

According to the World Health Organization, between 2000 and 2016, the rise in global health expenditures outpaced the global economy, reaching USD 7.5 trillion in 2016. ^38^ In 2010 alone, USD 863 billion were spent worldwide on CVD, a figure estimated to reach USD 1.04 trillion by 2030. ^39^

In Brazil, where nearly 50% of health expenditures are borne by the government, ^40^ the situation is no different. CVD accounts for the largest share of expenditure on inpatient care within the Brazilian Unified Health System, and is the leading reason for disability benefits. ^41 - 45^ It is estimated that, in 2015, public expenses on inpatient and outpatient care of CVD exceeded R$ 5 billion, while the cost of temporary sick leave or disability exceeded R$ 380 million. ^40^

Therefore, the economic impact of CVD, coupled with the need for rational use of financial resources, requires the large-scale implementation of low-cost models to ensure the feasibility of caring for a greater number of patients. CVR is a strategy that, in patients with stable CAD, is more cost-effective than procedures used much more widely, such as percutaneous coronary intervention. ^46 , 47^ In addition, its use on a larger scale would reduce health care expenditures due to a decrease in new cardiovascular events, hospital readmissions, and interventional treatment. ^48 , 49^ Therefore, wider dissemination of CVR should be considered a priority public health strategy.

Assessment of cost-effectiveness, which is done through a combined analysis of clinical consequences (effectiveness) and health-system expenditures, is essential in evaluating the relevance of large-scale implementation of a given treatment. ^50 - 52^ According to Georgiou et al., ^53^ measures that require investments of less than USD 20,000 per life-year saved (LYS) are considered to have excellent cost-effectiveness, whereas those that require investments of USD 20,000–40,000/LYS are acceptable and those requiring investments of USD > 40,000/LYS are unacceptable.

According to 1985–2004 data, CVR can be considered an intervention with an excellent cost-effectiveness ratio, as its addition to conventional treatment resulted in an increase in expenditures from USD 2,193 to USD 28,193 per LYS. In 2005, Papadakis et al. ^23^ published the first systematic review of studies on the cost-effectiveness of CVR as a secondary prevention strategy in patients with CAD and HF. In a 2018 evaluation of studies published after 2001, ^54^ the cost-effectiveness ratio was very similar to that described by Papadakis; the addition of CVR to conventional treatment resulted in an increase in expenditure of USD 2,555 to USD 23,598 per LYS.

It is worth mentioning that, although more than 75% of CVD deaths occur in low- and middle-income countries, ^55^ there is a lack of data on the cost-effectiveness of CVR in these settings. ^56^ Most of the information available comes from high-income nations, such as the United States, Canada, and European countries, which hinders extrapolation of results to the Brazilian reality. However, those few studies which are available from lower-income nations show a similar trend. In Brazil, the addition of rehabilitation to conventional treatment of patients with HF resulted in an increase in expenditures of USD 21,169 per LYS. ^57^

Despite the clear clinical and economic benefits of CVR, the percentage of eligible patients who effectively receive this type of care is far short of desired levels. According to international data, only around 30% of patients attend a CVR program; in Brazil, this number is estimated to be well below 15%. ^26 , 58 , 59^ In fact, most Brazilian states – including most capitals and large cities – lack even a single cardiac rehabilitation service.

As a result, the use of home-based models has grown. Initially, concerns about the safety of physical exercise meant that HBCR was intended only for low-risk patients. However, with growing evidence of noninferiority in terms of safety and similar clinical benefits in relation to the conventional strategy, ^60 - 62^ in addition to advances in technology that now allow remote monitoring, the use of HBCR has been expanded to patients with a higher risk profile.

Recent studies show that HBCR has effectiveness similar to traditional CVR, as demonstrated by Ades et al., ^60^ who compared the effects of a 3-month program of either model in low- and moderate-risk CAD patients after an acute coronary event. Although the group of patients who attended the traditional program performed a higher volume of exercise, there was no difference in increase in functional capacity or quality of life between the two groups. Jolly et al. ^62^ compared cardiovascular risk outcomes between patients undergoing traditional and home-based rehabilitation for longer periods, with 6, 12, and 24 months of follow-up, and also observed no differences.

A recent systematic review by Anderson et al. of studies enrolling patients with a history of AMI, CABG, or HF ^61^ also found no significant differences between conventional and home-based rehabilitation across a series of outcomes (death, cardiac events, functional capacity, quality of life, and modifiable risk factors) in the short term (3 to 12 months) or long term (up to 24 months).

Thus, HBCR programs should be considered as a strategy to facilitate access, adherence, and wider use of rehabilitation. Despite the aforementioned evidence of noninferiority in outcomes, comparatively few studies have demonstrated that the cost of HBCR is comparable to that of traditional CVR programs. ^61 , 63 , 64^ This major research gap precludes comparison of the two models in terms of cost-effectiveness. ^65 - 67^

Given the facts, it is unsustainable that countries of all income levels – and, most worryingly, lower-incomes – continue to provide high-cost therapeutic interventions massively, without stricter indications and criteria, while they continue to neglect the highly effective, economically viable, and readily applicable intervention that is CVR. There is an urgent need for public health policies to expand the availability of, participation in, and adherence of eligible patients to both traditional and home-based CVR programs.

Finally, considering the relevance of CVR given its broad clinical benefit and excellent cost-effectiveness, strategies must be implemented to change medical culture and stances toward it and facilitate the dissemination of structured rehabilitation programs. In this context, it is particularly relevant that specialty cardiology services offer CVR to their patients both during hospitalization and after discharge. The availability of a CVR service should be considered as a mandatory prerequisite for a medical institution to be recognized or accredited as having excellence in cardiology.

## 5. Home-Based Cardiovascular Rehabilitation

There are several barriers to patient access and adherence to conventional CVR, ^24 - 27 , 68^ which, compounded by the scarcity of referral to CVR programs and the limited availability of services, lead to very low levels of actual participation in supervised exercise programs. In this context, programs of indirectly supervised exercise carried out in the home, known as home-based cardiovascular rehabilitation (HBCR), are an attractive alternative or supplement to conventional, on-site CVR. Given its inherently greater availability, HBCR should be considered the main modality of CVR intervention when it comes to public health strategies, aiming at mass engagement of the CVD population in rehabilitation programs.

A Cochrane review ^61^ of 23 studies including 2,890 patients with heart disease (post-AMI, post-CABG, angina or HF) compared the effects of conventional CVR and HBCR. No differences were found in mortality, physical capacity, or quality of life. Therefore, the decision to participate in conventional (on-site) or home-based programs depends on the availability of services and patients’ individual preference.

HBCR is understood as the practice of physical exercise without face-to-face supervision, but with guidance and follow-up from the CVR team. It is thus also known as semi-supervised rehabilitation, or rehabilitation under indirect or remote supervision. The indications and objectives of HBCR are the same as those of conventional CVR; the same care is required regarding pre-exercise evaluation and exercise prescription. Most sessions are held under indirect supervision, but participation in some on-site classes, especially at the start of the program, is of fundamental importance to ensure that patients understand the exercise prescription, consolidate guidance and clarity doubts. Exercise can be done at home, in parks, on public roads, gyms, sports centers, and health clubs, among others, with patients self-monitoring and following the guidance received.

Therefore, in order to achieve HBCR as a viable population strategy, it is first necessary to expand the availability and capacity of conventional CVR programs, in order to enable initial evaluation, guidance, exercise prescription, and follow-up of home sessions (with periodic reassessment for any adjustments). Thus, the home strategy must be aligned with that of conventional CVR. The two modalities may be used in parallel, including patients with different risk profiles, or in sequence, with the same patient engaging in conventional or home-based CVR depending on clinical status.

Just as in conventional CVR, the first step of HBCR is referral by the primary physician, followed by evaluation by the rehabilitation physician and other team members, ideally including a stress test (CPET or TMET) or other physical fitness tests. After the pre-exercise evaluation, patients defined as high risk can be prioritized for conventional, face-to-face CVR. Those at lower risk, who are capable of self-monitoring and according to individual preference, can be routed to the HBCR component of the program. After receiving instruction on the prescribed exercises, patients perform the sessions on their own. The exercises may be documented in printouts or spreadsheets, with the aid of resources such as cardiac monitors, pedometers, or fitness trackers. Smartphone apps can mediate this exchange of information between patients and the health care team.

In some cases, a combined CVR program – with both on-site and home-based sessions – may be an option for moderate-risk patients who are still learning to self-monitor or find it difficult to attend face-to-face sessions due to social issues or reduced mobility. The proportion of on-site versus home-based sessions will vary according to the patient's clinical characteristics and the logistics and infrastructure of the CVR service.

The overall focus is always to make patients more physically active; a reduction in sedentary behavior and its harmful consequences is the imperative. It is essential that all available resources – whether alone or in combination, whether informal physical activity, home rehabilitation, or conventional CVR – be deployed toward this goal.

## 6. Integration of Cardiovascular Rehabilitation into Optimized Clinical Care of Cardiovascular Diseases

CVR must be integrated with full clinical treatment of CVD, which consists of a synergistic combination of structured lifestyle changes and optimized drug therapy. For instance, in patients with stable coronary disease – even those with moderate and severe ischemia – interventional treatments have not been shown to be superior in reducing major outcomes (cardiovascular mortality, all-cause mortality, AMI, HF). ^69 , 70^

To increase the efficacy and safety of CVR, it is important that drug therapy of CVD be properly optimized. The aim is to increase exercise tolerance and thus facilitate engagement in physical exercise while reducing the risk of further events. ^3 , 5 , 71 - 73^ In this context, it may be necessary to adjust current drugs or prescribe additional agents prior to the start of the physical exercise program. Once CVR has been initiated and adequate adherence has been achieved, some patients may require dose reduction or even discontinuation of some drugs due to adaptations to physical training, e.g., patients who develop hypotension, marked bradycardia, or symptomatic hypoglycemia. ^74 , 75^

### 6.1. General Guidance for Increasing Physical Activity and Engagement in Physical Exercise

There is an association between sedentary lifestyle (e.g., screen time), and higher all-cause and cardiovascular mortality. ^76^ Therefore, for health promotion and CVD prevention, medical guidelines have recommended the practice of moderate-intensity physical activity for at least 150 minutes per week or high-intensity for at least 75 minutes per week (grade 1B recommendation). ^77 - 83^ Engagement in more than 300 minutes/week of moderate- to high-intensity exercise can confer additional benefit, as has already been demonstrated in patients with CAD. ^84^

According to the results of individual evaluation, the exercise prescription may vary in terms of type (aerobic, endurance, flexibility), modality (walking, running, cycling, dancing, etc.) and duration; weekly frequency and intensity should also be considered (Tables 2 and 3).

**Table 2:** – Classifications of physical exercise

Classification		Features
By predominant metabolic pathway	Alactic anaerobic	High intensity, very short duration
	Lactic anaerobic	High intensity, short duration
	Aerobic	Low or medium intensity, prolonged duration
By pace	Fixed, constant, or continuous	No change of pace over time
	Variable, intermittent, or interval	Pace changes over time
By relative intensity*	Low or light	Easy to breathe, barely short of breath (Borg < 4)
	Medium or moderate	Breathing faster and labored, but still controlled. Can speak a sentence (Borg 4–7)
	High or heavy	Breathing very rapid and labored; short or out of breath. Barely able to speak (Borg > 7)
By muscle mechanics	Static	There is no movement; mechanical work is zero
	Dynamic	Involves movement; mechanical work is positive or negative

*A Borg scale of 0 to 10 was considered.

**Table 3:** – Methods for prescription of moderate-intensity aerobic exercise

Method	Description
Rating of perceived exertion (Borg)	Exercises yielding a rating of perceived exertion of 2–4 on the 0–10 Borg scale or 10–13 on the 6–20 Borg scale
Speech test	Exercise intensity maintained so that breathing is labored but still controlled, and the patient is still able to speak a complete sentence without pause
Percent peak HR	Exercise intensity titrated to an HR target of 70–85% of peak HR* Target HR = peak HR x desired percentage
HR reserve (Karvonen)	Exercise intensity titrated to an HR target of 50–85% of reserve HR (peak HR – resting HR). Target HR = resting HR + (peak HR – resting HR) x desired percentage
Cardiopulmonary exercise test thresholds	Exercise intensity titrated to remain between ventilatory threshold 1 (anaerobic threshold) and ventilatory threshold 2 (respiratory compensation point)

*Peak HR preferably measured during a maximal exercise stress test, as interindividual variability can cause errors in the prediction of HR by age, especially in patients who are on medications with negative chronotropic effect. HR: heart rate.

Sedentary patients should start exercising at the lower limit of the exercise prescription and progress gradually over the following weeks. Progression should initially be based on the duration of each session and, later, on exercise intensity. Physically active patients can perform exercises at a more intense level from the outset, aiming at a minimum duration of 75 minutes divided into two or more weekly sessions.

Resistance training of localized muscle groups (whether strength or endurance training) has proven quite beneficial for overall health and for the cardiovascular and musculoskeletal systems, and is particularly important – in fact, essential – in patients with sarcopenia and/or osteopenia. Resistance training should be performed at least twice a week, favoring the large muscle groups of the upper limbs, lower limbs, and core. Exercise can be done against the individual’s own body weight or using implements such as free weights, ankle weights, bands, or weight machines. The load (or weight) for each exercise or movement must be individually adjusted, and particular attention should be paid to proper posture and technique.

Several protocols for resistance training are available, with variations in parameters such as the number of exercises per session (e.g., 6 to 15), the number of sets per exercise (usually 1 to 3), and the number of repetitions per set (6 to 20). The intensity of resistance training can be adjusted according to the relative intensity of the maximum force, and can be expressed as a function of the maximum load that can be borne during a single repetition (one repetition maximum test or 1RM). Light intensity would be up to 30% of 1RM; medium intensity, between 30 and 60–70% of 1RM; and high intensity, above 60–70% of 1RM. Resistance exercises may also be prescribed subjectively, on the basis of perceived exertion alone (see Table 2 ).

A practical approach is the variable repetition method, which aims to perform a range of repetitions (e.g., 10 to 15 repetitions). If the patient is unable to perform the movement correctly for the minimum number of repetitions prescribed, the applied load is too high. On the other hand, if the patient reaches the maximum prescribed number of repetitions easily, the load is insufficient. Thus, the load will be adjusted so that training takes place within the proposed range of repetitions. This method can be applied to a wide range of localized exercises and can be altered as the patient progresses; the repetition ranges can also be modified depending on the desired objective (strength, hypertrophy, or endurance).

Flexibility exercises can also provide musculoskeletal benefits, improve health-related quality of life, and prevent falls in the elderly. By facilitating and increasing the efficiency of joint movement, they reduce oxygen demand during motion, thus enhancing cardiovascular performance. The aim of these exercises is to reach the maximum range of motion (point of mild discomfort) and remain static for 10 to 30 seconds.

Depending on the age group, clinical condition, and objectives of the exercise program for a given patient, other types of exercise can be included in the prescription, such as motor coordination and balance exercises. The countless benefits of more playful, social-based forms of exercise, such as dance and other modalities, should also be considered. ^85 , 86^

Assessment of aerobic and non-aerobic physical fitness enables a more individualized exercise prescription, with the objective of achieving the best outcomes while minimizing hazard to the patient through proper risk stratification and a thorough search for possible abnormalities. In general, the initial evaluation is based on a thorough history, physical examination, and ECG. More detailed assessments should be individualized to include CPET or TMET, anthropometric measurements, and evaluation of muscle strength/endurance and flexibility. The initial evaluation allows quantification of the patient’s functional deficit in relation to the desired level of function, as well as goal setting. Even those patients with poor baseline physical fitness can benefit from adequate adherence to a supervised exercise program. ^87^ Such adherence can also provide clinical and functional inputs that enable adequate counseling as to whether and how patients can resume sexual activity, based on the KiTOMI model, which was proposed by Brazilian authors in 2016. ^88^ In addition, periodic reevaluation is essential to encourage commitment and measure the progress and benefits obtained.

Finally, it is important to establish a systematic follow-up revaluation scheme which, in addition to encouraging patient commitment, will allow measurement of the progress and benefits obtained and yield reports to support treatment adjustments. Therefore, these reports should always be sent to the patient’s primary physician, who is obviously an integral part of full clinical treatment.

### 6.2. Hypertension

Hypertension (HTN) remains one of the leading risk factors for the development of CAD, HF, CKD, and ischemic or hemorrhagic stroke, representing a huge social and economic challenge to global public health. ^89^ Worldwide, the number of patients with HTN rose from 594 million in 1975 to 1.13 billion in 2015, with growth largely credited to underdeveloped and developing countries. ^90^ Considering that most cases of HTN are lifestyle-related, with sedentary behaviors as a prominent cause, the importance of physical exercise is clear, alongside other behavioral measures and drug therapy as indicated. ^72^

#### 6.2.1. Therapeutic Benefits of Physical Exercise

HTN has a complex, multifactorial pathophysiology involving structural and physiological modifications, particularly to the vasculature (increased arterial stiffness, increased arteriole wall-to-lumen ratio, capillary rarefaction), kidneys (increased plasma renin and water and sodium resorption, decreased glomerular filtration), and nervous system (increased sympathetic and chemoreceptor activity, decreased parasympathetic activity and baroreflex sensitivity). ^91^ The regular practice of physical exercise has a therapeutic effect on the physiological restructuring of these systems, reducing oxidative stress and inflammation, correcting baroreflex dysfunction, increasing vagal tone, decreasing sympathetic activity, reversing hypertrophic arteriolar remodeling in exercised tissues, and reducing peripheral vascular resistance, with a consequent decrease in BP levels similar, or even superior, to that provided by drug therapy. ^92 , 93^

In vascular tissue, HTN is characterized by disorganization of smooth muscle cells, increased collagen deposition, and a decreased elastin/collagen ratio, in addition to the formation of abnormal elastic fiber and internal elastic lamina with a smaller fenestrated area. ^94^ All of these structural changes in the vessel wall, which occur in both arteries and arterioles, increase the overall stiffness of the vasculature, with a consequent increase in pulse wave velocity, pulse pressure – the difference between systolic BP (SBP) and diastolic BP (DBP) – and hydrostatic pressure in the capillaries. These structural imbalances are compounded by endothelial dysfunction, with an increase in vasoconstrictive compounds, inflammatory mediators, and oxidizing agents, to the detriment of synthesis of vasodilating and antioxidant compounds. ^95^

Physical exercise, by increasing the tangential stress derived from the friction of blood flow on the endothelial surface of the vessel wall (commonly described by the term shear stress) positively stimulates the endothelial tissue, increasing production of antioxidant enzymes and vasodilating agents, in addition to decreasing the action of free radicals, pro-inflammatory cytokines, adhesion molecules, and vasoconstricting agents, thus restoring the balance of endothelial function. ^97 , 98^ Experimental studies ^94^ in spontaneously hypertensive rats have demonstrated reorganization of all vascular structures of the aorta after implementation of a period of aerobic exercise. Aerobic training promotes vascular adaptations in the conductance arteries (with decreased arterial stiffness and improved endothelial function), arterioles (by decreasing the vessel wall-to-lumen ratio), and capillaries, stimulating angiogenesis. ^99 , 100^

Thus, physical exercise has multifactorial effects on HTN, and is considered a key intervention to mitigate the burden of the disease and its comorbidities. ^101^ The antihypertensive effect of exercise is comparable to that of medication, ^102^ and both can be additive, occasionally requiring adjustments of drug dosage.

The greatest evidence of benefit in BP reductions among hypertensive patients is for aerobic physical exercise, as corroborated in a meta-analysis by Cornelissen et al. which showed an average SBP reduction of 8.3 mmHg and DBP reduction of 5.2 mmHg as a result of aerobic exercise.

The goal of resistance training (which also has an antihypertensive effect ^103^ ) is to preserve or increase muscle mass, strength, and endurance, factors that decrease the relative intensity needed to perform the activities of daily living, with consequent damping of the blood pressure response to exertion. Furthermore, resistance training may also promote improvement in baroreflex sensitivity. ^104^

In addition to aerobic and dynamic resistance exercises, some studies have focused on isometric (static resistance) exercises and shown significant effects in reducing BP levels. ^105 - 107^ A meta-analysis found that isometric handgrip training, performed for 12 minutes three to five times a week, reduced SBP and DBP by 5.2 and 3.9 mmHg respectively. ^108^ However, studies on the safety and effectiveness of isometric modalities in the long term are still lacking.

#### 6.2.2. Indications for Physical Exercise in Hypertension

Higher levels of physical activity have been associated with a decrease in the risk of developing HTN. With the advent of electronic activity trackers and ambulatory BP monitoring, it has become increasingly feasible to conduct studies that correlate physical activity with BP. ^109^ Physical fitness, measured objectively through graded stress tests, attenuates the increase in BP with age and prevents the development of HTN. In a cohort of 20-to-90-year-old men who were followed for 3 to 28 years, greater physical fitness decreased the rate of BP increase over time and delayed the onset of HTN. ^110^ Epidemiological studies have revealed that both level of physical activity and cardiorespiratory fitness are inversely associated with hypertension. ^111 , 112^

Large randomized controlled trials and meta-analyses have confirmed that regular exercise can reduce BP levels. ^102 , 112^ In addition, the continuous practice of physical activities can be beneficial for both the prevention and the treatment of hypertension, further reducing cardiovascular morbidity and mortality. Demonstrating this, active individuals have up to a 30% lower risk of developing hypertension than sedentary ones, ^111^ and increasing daily physical activity significantly reduces BP. ^113^

Physical inactivity is one of the greatest public health issues of modern society, ^114^ as it is the most prevalent of the cardiovascular risk factors and one of the leading factors contributing to mortality worldwide. ^115^ Survival is lower among people who spend most of their time sitting than in those who spend little time in this position. ^116^ Television viewing time is directly associated with high BP levels and cardiovascular morbidity and mortality; ^117^ therefore, to reduce time spent in the seated position, standing for at least 5 minutes for every 30 minutes spent sitting is recommended as a valid preventive measure. Physical exercise is indicated for all patients with HTN ( Table 4 ). ^72 , 73 , 118^

**Table 4:** – Indications for physical exercise in hypertension

Indication	Recommendation	Level of evidence
Aerobic exercise to prevent development of hypertension ^110-112^	I	A
Aerobic exercise in the treatment of hypertension ^93,102,103,112^	I	A
Dynamic muscle endurance training in the treatment of hypertension ^103,112^	I	B
Isometric training in the treatment of hypertension ^105-108^	IIa	B

In addition to exercise, the treatment of HTN requires other lifestyle changes, such as proper diet, weight control, and cessation of risk factors such as smoking and excessive alcohol intake.

In addition to the direct effect of exercise on HTN, another important component of CVR concerns the management of drug therapy, which can be optimized in the rehabilitation environment through disease education, advice on the need for treatment, and information on adverse effects and on the importance of adherence. ^119^

#### 6.2.3. Pre-Exercise Evaluation

Obviously, it is up to the patient’s primary physician to establish the diagnosis of HTN, search for other cardiovascular risk factors, and screen for target organ damage and other comorbidities in order to define the treatment strategy, which can be pharmacological and/or composed of one or more behavioral changes. ^72^

A CPET or TMET should be performed during pre-exercise evaluation, especially if there is suspicion of heart disease, target organ damage, or presence of three or more risk factors. ^72^ When CPET or TMET is used to support exercise prescription, it should ideally be performed with the patient on all of their usual medications, especially those with negative chronotropic effect, in order to mimic the actual conditions encountered during physical training. This will allow use of peak HR (TMET) or ventilatory thresholds (CPET) to determine the target training zone.

#### 6.2.4. Special Considerations for the Prescription and Follow-Up of Physical Exercise Programs

The exercise recommendation for hypertensive patients, is similar to that proposed for the general population: at least 150 minutes per week (five 30-minute sessions) of moderate-to-intense aerobic activity. In addition, two to three resistance training sessions per week are advisable. For greater benefit, absent any contraindications, patients may gradually increase their engagement towards a goal of 300 min/week of moderate aerobic exercise or 150 min/week of intense aerobic exercise.

During training, it is important that BP be assessed at rest and in exertion. Patients with a resting BP higher than 160/100 mmHg or with target organ damage (left ventricular hypertrophy, retinopathy, nephropathy, etc.) are advised to optimize antihypertensive therapy for better BP control before starting or resuming exercise, ^37^ or to reduce training intensity until better BP control is achieved. In supervised CVR programs, these recommendations are flexible and can be adjusted individually at the discretion of the rehabilitation physician and according to the BP response observed during the stress test and exercise sessions. During exercise, it is recommended that BP remain below 220/105 mmHg. If BP exceeds this level, the session should be halted or the load reduced, and adjustment of drug therapy should be considered. ^37^

BP must be measured after each exercise session, and is commonly found to be lower than before the start of activities. In hypertensive patients, the acute antihypertensive effect of a single session tends to be greater with more intense levels of aerobic exercise. ^120^ This acute effect of physical training can cause symptomatic hypotension once the session ends, which usually improves with rest and hydration. Patients on alpha blockers, beta blockers, calcium channel blockers, and vasodilators may be at increased risk of post-exercise hypotension, and thus require special attention during the cooldown period. If post-exercise hypotension becomes recurrent, which usually results from an add-on antihypertensive effect of training, the need for dose adjustments or even discontinuation of medications must be considered.

There is little data regarding the effect of exercise in patients with resistant hypertension, which is characterized by BP above target despite the use of three or more antihypertensive medications. In these patients, who require closer monitoring, a randomized, single-center clinical trial showed that exercising in warm water (30 to 32ºC) resulted in a pronounced reduction in BP (36/12 mmHg) after 3 months. ^121^ Although such effects need to be reproduced in further studies, exercise in warm water appears to be appropriate for patients with resistant hypertension.

## 6.3. Stable Coronary Artery Disease after an Acute Event or Revascularization

Cardiovascular disease (CVD), led by coronary artery disease (CAD), is responsible for the majority of deaths in the adult population worldwide. ^122 - 124^ The underlying mechanisms of stable CAD include atherosclerotic obstruction of the epicardial vessels, microvascular disease, and coronary spasm, either alone or in combination. ^5^ Clinically, the most common manifestation of stable CAD is angina pectoris, which is characterized by reversible episodes of chest pain due to myocardial ischemia, resulting from the imbalance between myocardial oxygen supply and consumption, usually triggered by physical exertion or emotional stress, which resolve with rest or the administration of fast-acting nitrates. ^5^

Stable CAD has a good prognosis, with annual mortality estimated at 1.5% and a nonfatal infarction incidence of 1.4%. ^125^ Nonetheless, full clinical treatment is essential, including optimization of drug therapy and regular physical exercise, in addition to other behavioral changes to address smoking, diet, and body composition. Elective revascularization (whether surgical or interventional) may also be indicated in patients with stable CAD, depending on their symptoms and cardiovascular risk. ^5^ However, it is worth noting that, in stable patients, even those with angina, exclusively clinical treatment has not been shown to be inferior to treatment with the addition of an interventional approach. ^70 , 126 , 127^

Development of an acute coronary syndrome, with AMI or unstable angina, is associated with increased cardiovascular risk and may require adjustment of drug therapy plus urgent surgical or percutaneous revascularization. ^128 - 131^

### 6.3.1. Therapeutic Benefits of Physical Exercise

The short- and long-term beneficial effects of regular physical exercise in patients with stable CAD have been demonstrated in the scientific literature. During the first 8 to 12 weeks of CVR, there is a marked increase in ischemic threshold, ^132 - 136^ improvement of cardiorespiratory functional capacity, ^132 , 134 , 136^ and improvement in myocardial perfusion imaging. ^137 - 140^ These benefits persist as long as regular physical exercise is maintained, ^103 , 141 - 144 ,^ which contributes to improvement in quality of life ^1 , 146^ and reduction of hospitalization and mortality from cardiovascular causes. ^1 , 144 , 146 - 148^

In patients with stable CAD, different mechanisms explain the increase in ischemic threshold, which gradually allows physical activity at higher loads. Reduction of the double product at submaximal loads is associated, among other mechanisms, with an improvement in cardiac autonomic modulation. ^144^ Myocardial perfusion increases due to an improved endothelium-dependent vasodilator response ^149 - 151^ and increased recruitment of collateral vessels during exercise, ^134 , 144 , 152^ which is reflected in the reduction of ST segment depression during exercise. ^35 , 132 , 137^ It is also notable that the combination of physical training and a low-fat diet can influence the progression of atherosclerotic plaque. ^152 , 153^

CVR is an adjunctive therapy that is also effective after acute coronary events and surgical or percutaneous revascularization. A systematic review and meta-analysis ^1^ of 63 studies involving 14,486 patients aged 47–71 years revealed that CVR reduced cardiovascular mortality by 26% and overall hospitalization rates by 18%, with additional improvement in quality of life. In this population, CVR should be encouraged whenever possible.

The improvement in cardiorespiratory fitness is one of the factors responsible for reduction of all-cause mortality after CVR. In a cohort of 5,641 CVR patients in Canada, every 1 MET increase in cardiorespiratory capacity was found to decrease all-cause mortality by 25%. ^154^ Other similar studies reported reductions in cardiac or all-cause mortality on the order of 8–34% for each MET of improvement in cardiorespiratory fitness. ^155 , 156^

In addition, CVR provides an add-on effect to reduce cardiovascular events after coronary angioplasty, as demonstrated in the ETICA trial (Exercise Training Intervention After Coronary Angioplasty). A 26% increase in VO _2_ peak, 27% improvement in quality of life, and 20% reduction in cardiac events, including fewer AMIs and fewer hospitalizations, were observed in patients who underwent CVR after angioplasty when compared to those who remained sedentary. ^157^

### 6.3.2. When Is Rehabilitation Indicated?

CVR is indicated in all cases of CAD ( Table 5 ). It is considered useful and effective both when it consists exclusively of physical exercise and when educational content, management of risk factors, and psychological counseling are added. ^146^

**Table 5:** – Indications for cardiovascular rehabilitation in coronary artery disease

Indication	Recommendation	Level of evidence
CVR to reduce myocardial ischemia ^132-140,158^	I	A
CVR to increase physical capacity ^132,134,140^	I	A
CVR to reduce mortality ^1,154,155^	I	A
CVR after coronary events or revascularization ^140,157^	I	A
Early CVR (1 week after an acute event) ^159,160^	IIa	A
CVR in patients with refractory angina ^161,162^	IIb	C

*CVR: cardiovascular rehabilitation.*

Despite increasingly early interventional treatments and decreased length of hospital stay after acute coronary syndromes, it is not uncommon for patients to begin rehabilitation only after outpatient follow-up with their primary physician, which may mean 15 days or longer after the event. Early initiation of CVR is possible, and can have a direct, positive influence on adherence and on the degree of clinical benefit achieved after the acute event.

One of the greatest concerns of early CVR refers to the effect of physical training on the ventricular remodeling process. While some authors report negative effects, ^163^ others report neutral ^139^ or even positive effects ^158 , 164^ on this process. A systematic review and meta-analysis ^159^ carried out to answer this question found that the changes observed in ventricular function, ventricular diameter, and functional capacity were directly related to the timing of CVR initiation. The greatest benefits in ventricular remodeling and functional capacity were obtained when programs were started in the acute phase (6 hours to 7 days) after the event, declining when initiated 7–28 days after the event and even further after 29 days, at which time the positive effect on ventricular remodeling was progressively lost. It is important to note that there was no difference in events between the initial training phases and that the sample studied was primarily composed of young men, which highlights the need for further studies, especially in other populations (such as older adults and women). For every 1-week delay in initiation of CVR after an AMI, an additional 1 month of training may be necessary to achieve similar benefits in end-systolic volume and left ventricular ejection fraction (LVEF). ^160^

Although widely endorsed by the medical literature for their beneficial effects and cost-effectiveness, CVR programs are only attended by a minority of eligible patients. Multiple barriers can explain this, such as a lack of programs, difficulty in accessing existing services, few referrals, and poor urban mobility; women, the elderly, and ethnic minorities are most affected. ^165 - 168^ Therefore, political, social, and structural changes – as well as a shift in medical culture – are needed to change this scenario.

### 6.3.3. Pre-Exercise Evaluation and Exercise Prescription

Both in patients with stable CAD and after a coronary event and/or revascularization, risk stratification for CVR is essential and should be based on a targeted clinical evaluation focused on detailed knowledge of the patient’s CVD and treatment history, whether clinical or interventional. Presence of symptoms, ventricular function, functional capacity, arrhythmias, and the possibility of residual ischemia all aid in stratification and should queried during the initial assessment. Ideally, this evaluation should be carried out by a CVR team member (rehabilitation physician).

The profile of a patient referred to CVR can vary widely, from individuals receiving elective treatment to patients with a complicated acute coronary syndrome and history of prolonged hospitalization. A broader assessment, including nutritional, psychological and musculoskeletal issues, should be part of the clinical history and examination, as these factors can directly impact the CVR process. In patients who have undergone percutaneous or surgical revascularization, examination of the arterial puncture site (especially of femoral access sites) or surgical wound (especially regarding sternal stability and wound infection) should always be performed. Any abnormalities or complex medical needs identified during the pre-exercise evaluation must be relayed to the CVR team members who will be involved in the patient’s exercise sessions.

During pre-exercise evaluation for CVR, the purpose of functional tests is to gain better insight into functional capacity, identification of residual ischemia and stress-induced arrhythmias. Myocardial ischemia on exertion is identified by detection of symptoms such as angina pectoris and/or by ECG changes. The ischemic threshold can be identified during TMET by the onset of these clinical and/or ECG changes and expressed as the load and/or HR at which ischemia initiate. This information is essential to guide the exercise prescription.

When used for exercise prescription purposes, TMET should be performed on all of the patient’s usual medications, especially those that may affect HR. This is important to reproduce the conditions that will be present during training sessions. If patients on beta blockers have their dose adjusted during rehabilitation, ideally a new TMET would be performed for adjustment of the exercise prescription. If this is impossible, a subjective (perceived exertion) test may assist in adjusting the prescription until a new test can be performed.

In some cases, patients entering CVR may have clinical limitations precluding a maximal exercise test. In these patients, the initial exercise prescription can be guided by a submaximal test, and a maximal test performed once there has been sufficient clinical improvement and/or optimization of drug therapy. Considering the possibility of serious error due to marked individual variation in chronotropic response, formulas that consider age as a parameter to define peak HR should never be used. The error is even greater in patients who are on beta blockers.

When rehabilitation is initiated without functional testing, the prescription may be based on the Borg scale of perceived exertion (a score of 11–15 on the 6–20 scale) and on arbitrary limitation of HR during training, i.e., the use of a resting HR + 20 bpm for patients who have had an acute coronary syndrome or resting HR + 30 bpm for those who have undergone elective surgical or interventional revascularization. ^131^ The target exercise intensity can also be determined by subjective assessment of breathing; moderate-intensity activity leaves the patient only slightly short of breath, but still able to speak complete sentences without interruption (see Table 3 ).

When a TMET is performed, the intensity of the prescribed exercise should lie between 40 and 80% of HR reserve (Karvonen formula: [Peak HR – resting HR] x percent intensity + resting HR). In such cases, the initial exercise prescription usually targets the lower limit of HR, progressing from there according to the patient’s clinical course and improvement in functional capacity. Most patients will be prescribed an exercise intensity between 50 and 70% of their HR reserve. Those that are more functionally limited or have significant ventricular dysfunction may be prescribed at lower intensities (40–60%), while those who were previously active and still retain better functional capacity may be prescribed at a higher and wider range (50–80%). Percentages of peak HR can also be used, with moderate-intensity exercise corresponding to 70–85% of HR peak (see Table 3 ).

CPET, by allowing analysis of the oxygen pulse response, provides increased sensitivity and specificity for the diagnosis of myocardial ischemia. ^169^ When there is an occurrence of an early plateau of oxygen pulse or, particularly, a drop during exertion, the exercise prescription should be limited to loads below that alterations. Thus, CPET is considered the gold-standard assessment method to support exercise prescription and should be used whenever it is available. ^169 - 171^ In patients who complete a CPET, the prescribed exercise intensity should lie between the ventilatory thresholds and increase progressively from there.

Regarding the volume of exercise, at least 150 minutes/week are recommended, distributed across 3 to 5 sessions. Depending on tolerance, adaptations to training, and individual preferences, as well as consideration of clinical status, this volume may be increased to 300 minutes or more per week.

For resistance training, the gold-standard method to determine the optimal intensity is 1RM testing. However, in practice, many rehabilitation programs do not perform this test due to time constraints or clinical limitations, such as in patients who have undergone CABG and may thus be limited not only by sternotomy but also by saphenectomy. In such cases, subjective perceived exertion is a practical and useful alternative.

In patients who have undergone sternotomy, upper body exercise should be limited to low intensity and performed with restricted loads for 5 to 8 weeks. Exercises involving the full range of motion of the arms may be allowed after this period if there is no sternal instability, although recent and ongoing studies are evaluating the safety of earlier exercise after CABG. ^172 , 173^

Patients should always be instructed on how to correctly perform movement and breathing, avoiding the Valsalva maneuver. The interval between series can range between 45 s and 1 min, depending on the load applied and the patient’s tolerance.

### 6.3.4. Special Considerations for the Prescription and Follow-Up of Physical Exercise Programs

***6.3.4.1. Refractory Angina***

Refractory angina is defined as disabling angina of over 3 months’ duration, despite optimized clinical treatment, with documentation of myocardial ischemia in a patient who is not considered eligible for percutaneous and/or surgical coronary intervention. ^174 , 175^ Such patients are generally not referred to CVR programs due to fear of adverse events during physical training, although rehabilitation has already been considered a feasible and safe possibility for these patients. ^175^

The objective of therapeutic interventions in this setting is to improve quality of life and facilitate performance of the activities of daily living. ^176 - 178^ A single controlled study has evaluated CVR in patients with refractory angina. The study randomized 42 subjects to a CVR exercise program or usual care for 8 weeks. Patients in the CVR group were prescribed training to a target HR between 60 and 75% of HR reserve (for those with preserved ventricular function) or between 40 and 60% of HR reserve (when LVEF was <40%). Patients in the rehabilitation group increased their total distance on the shuttle walk test by 50 m, with no change in severity or frequency of angina. There were no adverse events in either group. ^161^

An ongoing Brazilian randomized trial ^162^ will evaluate the safety and efficacy of a 12-week supervised exercise program in patients with refractory angina, carried out in a hospital environment with continuous ECG monitoring. Exercise prescription is individualized, on the basis of CPET findings and the ischemia and/or angina threshold. To date, 42 patients have been included, and no exercise-emergent cardiovascular events or hospitalizations related have been documented. Serum levels of high-sensitivity troponin T, a known predictor of worse prognosis, ^179^ did not change in 32 patients who completed a 40-minute acute aerobic exercise session (at the ischemia threshold) at the time of study enrollment ( *unpublished data* ).

In patients with refractory angina and a low ischemic threshold, administration of rapid-acting nitrates before the start of each physical training session can help prolong the duration of training and even allow exercise at higher intensities. ^180^

***6.3.4.2. Exercise Training with Myocardial Ischemia***

Traditionally, there is a recommendation that physical exercises in patients with CAD be performed below the clinical and electrocardiographic ischemic threshold; however, this can be difficult to control. Previous studies have shown that physical exercise, even when prescribed according to literature recommendations, can trigger scintigraphic perfusion defects which are not demonstrable on ECG and do not trigger angina, ^181 , 182^ because changes in contractility and perfusion defects precede clinical and electrocardiographic ischemic changes. ^183 , 184^

The functional significance of ischemic defects visible only on myocardial perfusion imaging is still unclear, but some studies of training above the ischemic threshold have been carried out. In one study of a single 20-minute training session conducted above the ischemic threshold, no evidence of acute myocardial damage was identified. ^185^ Other authors demonstrated in a small series of patients with CAD that, after 6 weeks of training, repetitive ischemic stimuli did not result in significant damage, myocardial dysfunction, or arrhythmias. ^186 , 187^

Therefore, there is evidence to suggest that interval training, a modality that has proven to be safe and effective in improving physical fitness, endothelial function, and left ventricular function with results superior to those obtained with moderate-intensity continuous training (MICT), may be feasible in patients with stable CAD. ^187 , 188^ Additionally, there is evidence of the superiority of combined aerobic and resistance training as opposed to aerobic training alone in patients with CAD. ^189^

***6.3.4.3. Drug Adjustments in Response to Physical Exercise***

Patients with stable CAD usually rely on medications for symptom relief, reduction of ischemia, improvement of endothelial function, stabilization of atherosclerotic plaque, control of risk factors, and maintenance of adequate hemodynamics. For example, high SBP and/or HR levels (increased double product) lead to clinical deterioration. On the other hand, systolic hypotension and bradycardia produce reduced cardiac output, which can cause abnormalities due to a drop in coronary flow.

In CVR programs, particular attention should be paid to improving the angina threshold before training begins, which allows greater tolerance to the progression of exercise intensity and, thus, facilitates achievement of the desired beneficial effects. Therefore, the optimization of drug therapy is essential for a safe and effective CVR.

Patients engaging in RCV can present a number of physiological adaptations to exercise, including modulation of the autonomic nervous system and reduced HR at baseline and on exertion. Together, these adaptations improve endothelial function and BP reduction, reduce afterload, and improve the diastolic function of the heart. ^190^ These adaptations can reduce the need for antianginal and antihypertensive agents. It is the role of the rehabilitation physician to discuss adjustments of drug therapy with the patient’s primary physician as necessary.

## 6.4. Heart Failure

Chronic HF is a complex, multisystem syndrome that features dyspnea and progressive exercise intolerance are its core symptoms. Despite recent advances in drug therapy, which have reduced once very high morbidity and mortality rates, symptoms tend to persist, compromising patient quality of life. There is consistent evidence that reduced levels of physical activity in HF trigger a vicious circle that contributes to increasing symptoms and exercise intolerance, secondary to a reduction in functional capacity, producing negative psychological effects, ^191^ impairment of vasoreactivity, peripheral endothelial dysfunction, ^192^ and chronic inflammation. ^193^ In this context, physical exercise has been established as a safe therapeutic strategy that mitigates the effects of progressive physical deconditioning due to the natural course of the disease. ^194^

Small randomized studies, systematic reviews, and meta-analyzes have consistently demonstrated that regular physical training is safe, increases exercise tolerance, improves quality of life, and reduces hospitalizations in HF. ^195 - 197^ A single large, multicenter randomized trial, HF-ACTION, ^198^ revealed a modest but nonsignificant reduction in primary outcomes (all-cause mortality and all-cause hospitalization), as well as major benefits in quality of life and a reduction in the rate of HF hospitalization. As a weakness of the study, poor adherence to exercise probably impaired the effectiveness of the intervention, a hypothesis that was confirmed later in another study, which demonstrated that adherence is a determining factor for obtaining medium-term benefits. ^199^

In a systematic review ^2^ on physical training in patients with HF, which analyzed 33 randomized studies including 4,740 patients (with a predominance of reduced ejection fraction), there was a trend toward reductions in all-cause mortality in the physical exercise group at 1 year of follow-up. Compared to controls, the physical training group had a lower rate of HF hospitalization and improved quality of life. Regarding benefits in women with HF, the available studies suggest that a positive impact equivalent to that seen in men. ^200^

For patients with advanced symptoms (NYHA class IV), data are still insufficient to indicate specific exercise programs. A single Brazilian randomized trial tested a daily exercise program on a cycle ergometer combined with noninvasive ventilation. The study evaluated patients hospitalized for acute decompensated HF, and found functional benefits and reduced length of stay. ^201^ Nevertheless, additional studies are needed to confirm these initial results before a stronger recommendation can be issued.

In HF with preserved LVEF, there is recent evidence from small randomized studies and a systematic review showing benefits in VO _2_ peak (measured by CPET), ^202 , 203^ quality of life, ^203 , 204^ and diastolic function (as assessed by echocardiography). ^205^

In light of this evidence, exercise-based CVR is recommended in HF ( Table 6 ) regardless of whether LVEF is preserved or reduced. Public policies must be adopted to ensure that a greater number of eligible patients benefit from treatment in structured CVR programs. ^207^

**Table 6:** – Indications for cardiovascular rehabilitation in heart failure

Indication	Recommendation	Level of evidence
Regular aerobic exercise in patients with HF to increase functional capacity, reduce symptoms, and improve quality of life ^2,195-199,205^	I	A
Regular aerobic exercise in patients with reduced LVEF to decrease HF hospitalization ^2,198^	I	A
Aerobic exercise in patients with preserved LVEF to increase functional capacity and improve diastolic function ^203,205,206^	IIa	B
Low-intensity aerobic exercises with noninvasive ventilation during the hospital phase of HF ^201^	IIb	B/C

*HF: heart failure; LVEF: left ventricular ejection fraction.*

Physical exercise alone should not be prescribed for patients with clinically unstable HF, with a clinical picture suggestive of acute myocarditis, or in the presence of acute systemic infection (Class IIIC).

### 6.4.1. Pre-Exercise Evaluation and Exercise Prescription

Internationally, CVR programs are implemented in a wide range of formats, using different exercise modalities alone or in combination. The exercises can include aerobic training (moderate- and/or high-intensity), localized resistance training, and respiratory muscle training ( Figure 2 ).

**Figure 2:** – Flow diagram of cardiovascular rehabilitation in patients with heart failure.

Before starting the training program, it is essential that the patient be clinically stable and on optimized drug therapy; ideally, a functional assessment should be performed, preferably with CPET or a TMET. If the aforementioned functional tests are unavailable, the 6-minute walk test can serve as a parameter for monitoring functional gains. ^208^ Functional tests should be performed while the patient is on his or her prescribed medications, to mimic the conditions of actual training.

The recommended aerobic training can be MICT, which corresponds to the HR zone delimited by the ventilatory thresholds of CPET, or, in the case of a TMET, to the zone located between 60 and 80% of peak HR or 50 and 70% of reserve HR. Patients with more severe disease and greater functional limitations may start at the lower end of the prescription. Intensity can progress up to the upper limit as training advances.

Recently, the use of high-intensity aerobic exercises performed in an interval mode – known as high-intensity interval training, or HIIT – has become popular. In this modality, more intense periods of exercise alternate with periods of passive or active recovery, which allows a greater total duration of high-intensity exercise and, consequently, can increase stimuli for central and peripheral physiological adaptations.

In HF patients with reduced LVEF, Wisløff et al. ^209^ demonstrated that HIIT was superior to MICT in promoting improvement in functional capacity and in different cardiovascular parameters. Subsequently, other clinical trials were carried out and meta-analyzed. The superiority of HIIT over MICT in terms of effects on functional capacity was confirmed in a meta-analysis. ^210^ The largest multicenter study published to date, Smartex-HF, ^211^ compared MICT versus HIIT. The authors found similar benefit, with no superiority of one modality over the other in any respect. Therefore, the choice of protocol will depend on team experience, clinical conditions, physical capacity, and patient preferences.

In addition, HIIT protocols can vary widely; several have been described. ^212^ One consists of 4 min of high-intensity exercise (90 to 95% of maximum HR) alternating with 3 min of low-intensity exercise (70% of maximum HR). ^209^ Protocols with much shorter durations of high-intensity load (30 or 90s) have already been described, and the tolerance to different HIIT protocols may vary according to patient preference and physical capacity. ^213^ Therefore, the use of this modality will depend on the patient’s clinical picture and choices, as well as on the experience and preferences of the CVR team.

The addition of localized muscle resistance exercises to aerobic training has been suggested as a means of obtaining additional benefit. ^214^ These exercised can be prescribed as percentages of maximum voluntary contraction or according to subjective perceived exertion. The recommended loads and repetitions may vary according to the patient’s functional limitations and must be individualized, progressing as rehabilitation itself progresses.

The addition of breathing exercises has been recommended for patients with respiratory muscle weakness. ^215^ In a meta-analysis by Smart et al. ^216^ which evaluated 11 studies including 287 participants with HF, 148 of whom underwent inspiratory muscle training (IMT) compared with 139 sedentary controls, significant gains in VO _2_ peak, distance walked in the 6-minute test, quality of life, PImax, and VE/VCO _2_ slope were observed. Thus, IMT provided gains in cardiorespiratory fitness and quality of life of a similar magnitude to those obtained with conventional training, and should be considered a valid alternative for severely deconditioned and debilitated HF patients, perhaps as a bridge to conventional physical exercise.

### 6.4.2. Final Considerations on Heart Failure

Given the variety of benefits observed, it is essential that patients with HF perform physical exercises regularly. Ideally, this should be done in the context of a CVR program, with an individualized prescription combining moderate- and/or high-intensity aerobic training, localized muscle resistance exercises, and respiratory muscle training, depending on the patient’s clinical condition and functional limitations, and according to patient preferences and staff experience. In addition, there are valid alternatives even for very debilitated and severely deconditioned patients. ^214 , 217^

## 6.5. Heart Transplantation

Heart transplantation (HTx) is the treatment of choice for patients with refractory HF, whose symptoms remain severe despite use of the entire pharmacotherapeutic arsenal and surgical procedures as indicated.

In recent years, there have been significant advances in HTx, with the emergence of new surgical techniques and the development of more efficient immunosuppressants. In Brazil, there has been substantial growth in the number of procedures, which had been stagnant since 2015, with a rate of 1.7 transplants per million population (pmp). In 2019, the rate grew 17.6%, reaching 2 transplants pmp, very close to the target set for the year (2.1 pmp). In 2018, 357 procedures were performed, and by March 2019, 104 hearts had been transplanted in Brazil. ^218^

HTx aims to improve quality of life, as well as survival, in this population. ^219 , 220^ Recipients are able to return to work and lead normal lives with minimal or no symptoms. ^221^ The survival rate is estimated at 90% at 1 year and around 70% at 5 years. ^222^

Although HTx significantly improves patients’ functional capacity, VO _2_ peak is still reduced when compared to that of healthy, age-matched individuals. ^223 , 224^ Among other factors, this can be explained by: 1) in the immediate post-transplant period, the allograft is devoid of sympathetic and parasympathetic innervation (autonomic denervation), causing an increase in resting HR, attenuating its natural elevation in response to exercise, and impairing recovery after exertion ^224 , 225^ ; 2 ) patients often exhibit skeletal muscle dysfunction (sometimes to the point of cachexia), in which immunosuppressive therapy and pre-transplant HF play prominent roles ^226^ ; and 3) impairment of vascular and diastolic function. ^227^ During the acute phase of exercise, the increase in cardiac output of HTx recipients depends fundamentally on the Frank–Starling mechanism, i.e., on increase in venous return, inotropy, chronotropy, and reduction in afterload. ^228 , 229^ In addition, there is an increase in the concentrations of circulating catecholamines, ^227^ which decrease slowly after the end of exercise, explaining the slow recovery of HR in these patients. ^230^

Immunosuppression may predispose HTx recipients to a higher risk of other complications, ^231^ and these patients may develop HTN, diabetes mellitus, and CAD. ^232^ Conversely, physical exercise is known to be an excellent therapy for management of these chronic diseases ^93 , 233^ and is effective in optimizing autonomic control. ^230 , 234^

Physical training after HTx contributes to an increase in VO _2_ peak and improvements in hemodynamic control, muscle strength, and bone mineral density, ^233 - 236^ thus improving prognosis. ^19^ Although there are countless possibilities for training prescription, the recommended method remains aerobic exercise, which can be performed continuously or, in specific cases, at intervals and at different intensities, ^170^ combined with resistance training whenever possible. ^6^

### 6.5.1. Benefits of Physical Exercise

In a pioneering study by Richard et al., ^237^ the investigators found that, 46 months after HTx, patients who underwent aerobic training had a functional capacity and chronotropic function similar to those of healthy individuals. Previous studies had already demonstrated the safety of physical training in this population. ^234 , 238 - 240^

A Cochrane meta-analysis of nine randomized clinical trials, including 284 patients, compared the effect of physical training to usual care in the post-HTx setting. ^234^ An average increase in VO _2_ peak of 2.5 ml.kg ^- 1^ .min ^- 1^ was observed in those who received training versus those allocated to usual care. Rosenbaum et al. ^241^ assessed the relationship between early participation in a CVR program after HTx and found that the number of sessions performed in the first 90 days was directly associated with better 10-year survival.

Haykowsky et al. ^242^ described significant improvements in VO _2_ peak of HTx recipients, with an average increase of 3.1 ml.kg ^- 1^ .min ^- 1^ after 12 weeks of combined training (resistance and aerobic). Kobashigawa et al. ^243^ studied 27 patients after HTx who received a combination of aerobic, resistance, and flexibility training for 6 months versus a control group. The duration and intensity of the aerobic exercise sessions had a goal of at least 30 minutes of continuous, moderate exercise on a cycle ergometer. The intervention group showed an average increase of 4.4 ml ^- 1^ .kg ^- 1^ .min in VO _2_ peak, versus 1.9 ml.kg ^- 1^ .min ^- 1^ in the control group. These data provide valuable information on the importance of both types of training for this population.

Regarding high-intensity training in patients after HTx, the number of studies is still small, but the results obtained have been encouraging. In a crossover study, Dall et al. ^244^ found a greater effect of HIIT compared to MICT on VO _2_ peak, with an additional gain of 2.3 ml.kg ^- 1^ .min ^- 1^ and superior improvement in quality of life. One meta-analysis ^233^ included three randomized controlled trials that compared HIIT (intense blocks: 80 to 100% of VO _2_ peak or 85 to 95% of peak HR) to usual care. Post-HTx patients randomized to HIIT showed an increase in VO _2_ peak of 4.45 ml.kg ^- 1^ .min ^- 1^ after the intervention period, which ranged from 8 to 12 weeks of three to five weekly sessions.

Nytrøen et al. ^224^ evaluated the effects of a HIIT program compared to a control group in 43 HTx recipients. The authors evaluated the progression of allograft vasculopathy, assessed by intravascular ultrasound, and found less progression of atheromatous plaque in the HIIT group. However, additional studies are still needed to elucidate these benefits. ^245^

Some well-known common adverse effects of the use of glucocorticoids after HTx are muscle atrophy and weakness. In 1998, Braith et al. ^235^ were the first to study the effect of resistance training on glucocorticoid-induced myopathy in HTx recipients. One group received training and was compared with a control group. After 6 months, although both groups had increased muscle strength in the quadriceps and lumbar extensors, the increase was up to six times greater in the training group.

Resistance training also appears to have a major therapeutic effect on bone metabolism. After HTx, patients commonly experience significant bone loss at femur head and mineral total bone loss. In one study, patients were enrolled for resistance training 2 months after HTx, and training was shown to be able to restore bone mineral density to pre-transplant levels. ^236^

### 6.5.2. Pre-Exercise Evaluation and Unique Features

HTx recipients must undergo a thorough history, physical examination, 12-lead resting ECG, color Doppler echocardiogram, and other tests at the discretion of the CVR team. Ideally, a functional stress test should be performed, preferably CPET, which is the gold-standard method for assessing functional capacity in this patient population. The stress test must be performed by a trained physician; it evaluate the cardiopulmonary and metabolic responses to increasing exercise and yields several variables that have an impact on the clinical examination and the exercise prescription. ^246^ The physical therapist and/or physical educator will prescribe, administer, supervise, and guide exercise, following the safety limits recommended by the physician on the basis of the pre-exercise evaluation. ^6 , 247^

The impossibility of performing CPET should not be considered an impediment to exercises; if CPET is not available, a TMET is suggested. ^170^ When even this is unavailable or otherwise impossible, the 6-minute walk test can assist in clinical assessment, in addition to providing a parameter for comparing functional capacity during training. ^248 , 249^

### 6.5.3. Exercise Prescription

Aerobic exercise is most recommended, with supplemental resistance training, starting on the 6th week after HTx. Different training methodologies have been studied in isolation and have proven effective in promoting cardiovascular health in individuals undergoing CVR. ^6 , 170^ In patients who have undergone HTx, most studies evaluated the effect of MICT.

Depending on the patient’s clinical condition, the intensity of aerobic exercise may be gradually increased from moderate to high over the course of training, in order to optimize adaptation and obtain greater benefit, as exercise intensity is directly associated with the magnitude of cardiovascular adaptations. ^250^ In this sense, programs that include interval training (even HIIT) have demonstrated good outcomes. ^233^ However, an optimized and safe exercise prescription requires proper individualization of each component of the training session. ^170^

The determination of target training zones is advised as a means of optimizing the exercise prescription. ^170^ However, as recent HTx recipients will exhibit a compromised chronotropic response, ^251^ prescriptions based on percent of peak HR or threshold HR will not be useful during the first training sessions, although they may be used once there has been improvement in autonomic response. ^224^ Continuous assessment of the HR response to exercise and during recovery is thus extremely important. When CPET is available, the prescription of aerobic exercise can be based on the ventilatory thresholds or on established percentages of VO _2_ peak. Another simple and feasible strategy is assessment of subjective perceived exertion using the Borg scale. ^4 , 170 , 252^ The multidisciplinary team must be firmly committed to educating the patient regarding the various levels of perceived exertion and the symptoms of which they should be aware. ^4 , 6^

In addition to the evaluation and prescription of aerobic exercises, resistance exercises are essential. The methods traditionally used for pre-exercise assessment and exercise prescription are 1RM load tests. However, the use of these protocols after HTx – especially after a recent procedure – may be inappropriate, and clinical investigations on the safety of these tests in this specific patient population are still lacking. An alternative evaluation method is the 30-second sit-to-stand test. ^253^ This test has been validated in active older adults and proved to be reasonably reliable in providing information about lower limb strength, and is now widely used in rehabilitation centers and scientific research on a wide range of clinical conditions. ^254 - 256^

Resistance exercises may also be prescribed subjectively, on the basis of perceived exertion alone. The variable repetition method may be used, whereby the aim of the user is not a set number but a range of repetitions (e.g., 10 to 15 repetitions). If the patient is unable to perform at the lower end of the range, the applied load is too high; if the patient can execute the maximum number of repetitions with ease, the load is too light. Thus, the load can be adjusted so that training takes place within the proposed range of repetitions.

During training, particular attention should be paid to any complications or intercurrent events, such as infections related to the transplant procedure. A. survey found that 36% of HTx recipients are hospitalized within the first year after transplantation, and 61% at 4 years. ^257 , 258^ This clearly demonstrates the importance of patient supervision throughout training, should any intercurrent event arise that warrants discontinuation of the exercise session. In view of the foregoing, some authors suggest that patients should not perform physical exercise when receiving pulse steroid therapy and on the days of myocardial biopsies. ^170^

### 6.5.4. Home-Based Cardiovascular Rehabilitation

Previous studies have shown that HBCR programs are safe and effective, ^1^ and are recommended as an alternative to traditional CVR in low-risk patients. ^71^

Wu et al. ^259^ conducted a prospective, randomized study to evaluate the effect of a home exercise program for 2 months in 37 patients after HTx. The control group maintained their usual lifestyle throughout the study period. Individuals in the intervention group performed an exercise program at least three times a week which included a 5-min warm-up, upper limb and lower limb strength training, 15 to 20 min of aerobic exercise at an intensity of 60 to 70% of VO _2_ peak, and a 5-min cooldown period. To ensure proper exercise performance at home, an initial period of direct supervision was enforced. At the end of the 2-month period, patients had improved muscle strength and endurance, fatigue index, and quality of life (physical domain). CPET revealed an increase in workload, but with no change in VO _2_ peak, probably due to the short follow-up period or the less-intense methodology used to guide the training prescription.

Another study, ^260^ with an equivalent aerobic training protocol but a longer duration (five times a week for 6 months), documented improvements in VO _2_ peak, workload, and BP in individuals after HTx. In addition, there were signs of cardiac sympathetic reinnervation and restoration of arterial sensitivity to autonomic modulation, with no changes in the control group.

Even beyond 5 years after HTx, HBCR can still improve functional capacity, as demonstrated by a study in which 21 patients were instructed to follow a home-based physical training program consisting of cycle ergometer exercises for 1 year. Nine patients served as controls. To ensure adequate control, patients received a smart card programmed for a 6-min warm-up and 20 min at a constant workload, with load adjustment according to the exercise prescription and HR monitoring. At the end of 12 months, there was a modest improvement in VO _2_ peak. ^261^

Karapolat et al. ^262^ compared the effects of home-based and hospital-based exercise programs on exercise capacity and chronotropic variables in 28 patients after HTx and observed significant improvements in VO _2_ peak and HR reserve only in the traditional CVR group. However, new studies, with the inclusion of a larger number of patients, are necessary to better elucidate this superiority of the hospital-based program observed in this study.

### 6.5.5. Recommendations

Based on the evidence reviewed above, physical training has an unequivocal beneficial effect after HTx, is safe and feasible, and can be performed in the hospital or home environment ( Table 7 ). However, although both strategies are effective in promoting an increase in functional capacity, the magnitude of the effect is greater when training is performed in supervised environments.

**Table 7:** – Indications for cardiovascular rehabilitation in heart transplant recipients

Indication	Recommendation	Level of evidence
CVR consisting of moderate aerobic exercise is recommended for patients after HTx ^234,239,241,243^	I	A
CVR consisting of high-intensity aerobic exercise is recommended for patients after HTx ^233,238,244^	IIa	B
CVR consisting of resistance training is recommended for patients after HTx ^235,236^	I	B

*CVR: cardiovascular rehabilitation, Htx: heart transplant (HTx).*

CVR should be started 6 to 8 weeks after the HTx, with referral at hospital discharge. In selected cases and after careful evaluation by the CVR team, rehabilitation may begin earlier. As in any post-sternotomy situation, special care must be taken not to prescribe exercises that might overload the chest muscles and lead to sternal traction, especially in the first 90 days after transplant.

The ideal prescription will include exercises that promote different components of physical fitness, always maintaining an emphasis on specific recommendations for each condition. After HTx, just as in other indications for CVR, aerobic exercise should be the main component of training sessions, supplemented by resistance and flexibility training within an individualized, periodically reassessed program. Sessions should always start with a warm-up period and end with a controlled cooldown period. This strategy aims not only to warm up the skeletal muscles but also to provide an adequate time for adjustments of HR and BP to exercise, as the exertion response in these patients is affected by denervation of the heart, especially in the early stages of the training program after transplant.

Aerobic exercise may consist of walking or cycling, whether indoors (using treadmills and/or cycle ergometers) or outdoors. A weekly frequency of three to five sessions, each lasting 20 to 40 minutes, is recommended. The frequency and duration of these sessions will be adjusted according to the patient’s preexisting condition and should progress over time with training. Control of exercise intensity is essential; given the larger evidence base, MICT (between the first and second ventilatory thresholds) is recommended, with a perceived exertion no greater than 11 to 13 on the modified Borg Scale. In selected cases, interval training can be adopted to add variety and, potentially, to enhance functional gain.

Resistance training is essential, especially in the early phase after transplantation. Many HTx recipients had longstanding HF, endured prolonged hospitalizations, and have been exposed to massive surgical stress. In this regard, a resistance exercise program can be particularly useful. At the start of training, activities performed against body weight alone are considered sufficient for these patients. Over time, elastic bands, dumbbells, ankle weights, and weight machines can be added to the training program. Greater than usual care is needed during upper body exercises, considering that corticosteroid therapy may make for slower healing of the thoracotomy scar.

Further information and examples of training protocols for these patients are available elsewhere. ^263 - 265^

## 6.6. Cardiomyopathies

This section will address hypertrophic cardiomyopathy (HCM), myocarditis, and other cardiomyopathies. The indications for CVR in this setting are listed in Table 8 .

**Table 8:** – Indications for physical exercise in cardiomyopathy

Indication	Recommendation	Level of evidence
Moderate aerobic exercise is recommended for selected patients with HCM ^266,267^	IIa	B
Vigorous or competitive physical exercise in patients with HCM ^268,269^	III	C
Moderate aerobic exercise for selected patients 3 to 6 months after acute myocarditis	IIb	C
Light to moderate aerobic exercise for selected patients with ARVC ^270^	IIb	B
Vigorous or competitive physical exercise in patients with ARVC ^268,269^	III	C

*HCM: hypertrophic cardiomyopathy; ARVC: arrhythmogenic right ventricular cardiomyopathy.*

### 6.6.1. Hypertrophic Cardiomyopathy

Hypertrophic cardiomyopathy (HCM) is a disease characterized by left ventricular hypertrophy, usually without dilatation of the ventricular chambers, in the absence of another cardiac or systemic disease capable of explaining the magnitude of hypertrophy observed. ^271^ It is the most common hereditary heart disease in the general population, caused by a range of mutations in genes which encode the cardiac sarcomere proteins. ^268^ HCM has a characteristically heterogeneous clinical expression, with unique pathophysiological changes and a variable natural history. Up to 10% of cases are caused by other genetic disorders, including hereditary metabolic and neuromuscular disorders, chromosomal abnormalities, and genetic syndromes. ^272^ Some patients have other disorders that can mimic HCM, such as amyloidosis. ^273^

The population-wide prevalence is estimated at around 0.2% or 1 in 500. ^268^ However, this estimate appears to differ in clinical practice, which allows us to infer that a portion of the affected individuals are asymptomatic. Various patterns of asymmetric hypertrophy of the left ventricle are commonly seen in HCM, and there may be different phenotypes in first-degree relatives. Typically, one or more regions of the left ventricle exhibit increased wall thickness when compared to others; transitions and variations in thickness may occur in adjacent or noncontiguous areas. However, although asymmetric septal hypertrophy is the most common finding, there is no “classic” HCM pattern, and virtually all possible patterns of left ventricular hypertrophy can occur. Hypertrophy may even be absent in genetically affected individuals, in what is known as a negative phenotype.

Several multicenter retrospective and observational cohort studies, conducted in different populations, have elucidated the natural history and clinical course of HCM. Recent studies have reported an annual mortality of around 1%, much lower than in older surveys. ^274^ Notably, only a small subgroup of patients with HCM experience significant complications and premature death; these complications can occur due to obstruction of the left ventricular outflow tract, HF with diastolic and/or systolic dysfunction and sudden cardiac death (SCD), or cardiac arrhythmias (atrial fibrillation and ventricular tachycardia or fibrillation). ^275^ In HCM, SCD can occur at any age, although it is most common in adolescents and young adults; therefore, identification of individuals at the highest risk is an essential component of the pre-exercise evaluation, especially in patients who may want to engage in competitive sports. ^276^

In many cases, SCD can be the first manifestation of the disease; indeed, it occurs most commonly in those without warning symptoms and who had not been diagnosed prior to the event. Nevertheless, most patients with HCM have a normal or near-normal life expectancy, with mortality usually attributable to other causes, some even of non-cardiovascular etiology. ^277 - 279^ Therefore, encouraging a healthy lifestyle for HCM patients is essential to reducing the overall risk of morbidity.

***6.6.1.1. Therapeutic Benefits of Physical Exercise***

In the general population, cardiorespiratory fitness is a determinant of the risk of cardiovascular and all-cause mortality. ^19^ In patients with obstructive and minimally symptomatic HCM, an association of mortality with aerobic fitness has also been observed. ^280 , 281^ Patients with a VO _2_ peak below 18 ml.kg ^- 1^ .min ^- 1^ on CPET had higher mortality and were more symptomatic compared to those who achieved values equal to or greater than this threshold. A VO _2_ peak below 60% of predicted was associated with worse 4-year survival (as low as 60%). ^280^

Myocardial fibrosis and myofibrillar derangement may underlie the increased risk of SCD in HCM, as these structural changes act as a substrate for fatal arrhythmias. ^271^ Evidence does suggest that high-intensity physical training could accelerate these changes, but this is still a controversial topic. However, it is well established that the increase in myocardial fibrosis is associated with a lower VO _2_ peak in this population. ^282^

Therefore, assessing aerobic fitness – preferably through CPET – is essential in patients with HCM. ^281^ When there is a reduction in VO _2_ peak, physical exercise can help increase functional capacity.

To date, only one randomized controlled trial has examined the effect of physical training on patients with HCM (RESET-HCM). This study, which included 136 patients, demonstrated an increase in VO _2_ peak after 16 weeks of a moderate-training intervention (+1.35 ml.kg ^- 1^ .min ^- 1^ or < 0.5 MET). ^266^ Another prospective, non-randomized study included 20 patients with HCM and found a significant increase in treadmill test duration, as well as in estimated functional capacity (+2.5 MET). ^267^ In this study, patients completed a CVR program which consisted of 60-minute sessions of moderate to vigorous exercise, performed on a treadmill or cycle ergometer, twice a week. The intensity of exercise progressed from 50 to 85% of HR reserve, which resulted in a gradual increase in conditioning and may have minimized the risk of adverse events, such as exercise-induced arrhythmias. Serious adverse events, such as death, aborted SCD, implantable cardioverter-defibrillator (ICD) activation, or sustained ventricular tachycardia, did not occur in any of these studies. ^266 , 267^

***6.6.1.2. When Is Physical Exercise Indicated?***

The intensity of exercise which patients with HCM can be cleared to do still represents a major challenge. If, on the one hand, intense physical exercise can be harmful, with an increased risk of potentially fatal arrhythmias, on the other hand, excessive restrictions on physical activity lead to deconditioning and can have negative effects on health and quality of life; they may even increase cardiovascular risk, given the well-established association between physical fitness and mortality. ^280 , 281^

In its official position statement on management of HCM, the American Heart Association discourages patients with the disease from engaging in competitive sports of moderate to vigorous intensity (see Table 8 ). This limitation is meant to minimize sudden changes in BP and increases in cardiac output in order to protect patients from the negative effects of exercise on a pathologically hypertrophic heart. ^283^

Exercise-triggered arrhythmias (in the short term) and adverse myocardial remodeling (in the long term) are the most fearsome side effects of exercise in HCM. The fear of SCD during sport extends to non-competitive athletic activities, although there is a clear lack of evidence about the safety of exercise in this patient profile. However, it should be emphasized that this risk of exercise is theoretical, and that recommendations to limit physical activity have been advocated with caution, based solely on the opinion of experts, and are not supported by more robust evidence. ^284^

Thus, patients with HCM receive little guidance regarding the best dose or amount of physical activity to maintain general health and well-being; instead, greater focus is placed on restrictions on physical activities. As a result, more than 50% of patients with HCM do not achieve the minimum recommended physical activity target due to the belief that they are unable to exercise and/or that physical activity can worsen their disease.

Therefore, a balanced approach seems to be most appropriate, and extremes should be avoided (neither vigorous competitive exercise nor physical inactivity), as both could increase cardiovascular risk.

New evidence suggests a positive effect of moderate physical exercise in selected patients with HCM, with individualized risk assessment and exercise prescription. It is noteworthy that the evidence suggests benefits of MICT, while other modalities need further studies.

However, the presence of any of the following could be considered major contraindications to the practice of exercise: history of aborted SCD in the absence of an ICD; history of syncope on exertion; exercise-induced ventricular tachycardia; increased exercise pressure gradient (greater than 50 mmHg); and abnormal BP response to exertion.

***6.6.1.3. Pre-Exercise Evaluation***

Clearance to begin exercising must be based on the pre-exercise evaluation, including a thorough history, physical examination, and 12-lead ECG.

A large proportion of individuals with HCM are asymptomatic or oligosymptomatic; clinical suspicion is raised only by changes on resting ECG, which is abnormal in up to 95% of patients with the disease. ^285^ Electrocardiographic changes may precede structurally detectable disease for some years, which makes ECG extremely important in this scenario. ^269^ Only a minority of patients with HCM present with a normal ECG – usually those without any other phenotypic manifestations (positive genotype/negative phenotype).

Echocardiography remains the most widely used modality for diagnosis of HCM. Magnetic resonance imaging (MRI) is usually reserved for cases in which echocardiography is in conclusive, or to assess more localized hypertrophy (e.g., apical forms). In young athletes, distinguishing physiological hypertrophy (“athlete’s heart”) from the pathological hypertrophy of HCM is a challenge. This is because, most athletes with HCM exhibit an asymmetric pattern of left ventricular hypertrophy, as do sedentary individuals with the condition. In contrast, those with physiological left ventricular hypertrophy show a more homogeneous, symmetrical distribution of wall thickness, with only minor differences between contiguous segments and a symmetrical pattern of left ventricular hypertrophy. ^286^

Exercise testing is always recommended in these patients prior to the start of CVR, whether to assess functional capacity or to detect abnormal BP responses and signs of increased dynamic obstruction of the outflow tract with exertion. For better detection of outlet tract obstruction during exercise, a combination of imaging (echocardiography) with stress testing is the gold standard and should be encouraged whenever possible. Patients with no obstruction at rest can present significant gradients on exertion, and thus be reclassified in relation to prognosis. ^287^

When available, CPET is advised instead of TMET, as it allows direct measurement of VO _2_ peak, a parameter with documented prognostic value. ^280 , 281^ In addition, determining ventilatory thresholds contributes to a more individualized exercise prescription.

***6.6.1.4. Special Considerations for the Prescription and Follow-Up of Physical Exercise Programs***

Some particularities of exercise in patients with HCM should be noted:

- So-called “explosive” activities (e.g., basketball, football, tennis), with the potential for rapid acceleration and deceleration, should be avoided.
- Activities with steady, constant energy consumption (e.g., light jogging or swimming) are preferred.
- Exercise in adverse environmental conditions, including extreme heat or cold, should be avoided, as there is an increased risk of exacerbating exercise-induced physiological changes.
- Training programs that aim competitivity, or achievement of higher levels of fitness and excellence, should be avoided, as they usually motivate patients to strive beyond safe limits.
- Intense static (isometric) exercises, such as weight lifting, should be avoided, as there is an increased risk of left ventricular outflow tract obstruction due to the intense Valsalva maneuver involved.
- Resistance training with low loads and a greater number of repetitions is considered safe for patients with CVD, although there is no solid evidence for patients with HCM.

Some notes on drug therapy are warranted. Beta blockers and calcium channel blockers may be indicated in the treatment of HCM. As these medications attenuate the HR response to exercise, patients may experience a very reduced chronotropic response to exertion, which can cause increased exercise intolerance, suggesting a need for dosage adjustment. Excess diuretic use can be harmful because it increases the gradient of the outflow tract. Therefore, these agents should be used with caution. Like diuretics, exercise-induced dehydration can raise the outflow tract gradient; therefore, adequate hydration during training is of paramount importance.

### 6.6.2. Myocarditis

The pathogenesis of myocarditis consists of three phases: acute myocardial injury, usually of viral etiology; host immune response; and recovery, or transition to fibrosis and dilated cardiomyopathy. Clinically, there is no clear distinction between these phases. The initial insult can cause acute myocardial damage, with impairment of contractility mediated by cytokines produced by the local inflammatory process. This acute inflammation may progress, in the late phase, to extensive fibrosis, which can cause ventricular dilatation and dysfunction.

Acute myocarditis should be suspected when the following criteria are present: ^283^

- A clinical syndrome of acute HF, angina-type chest pain, or myopericarditis of less than 3 months’ duration
- Unexplained rise in serum troponin
- ECG changes suggestive of myocardial ischemia
- Global or regional wall motion abnormalities and/or pericardial effusion on echocardiography
- Characteristic changes in tissue signal on T2- or T1-weighted MRI, as well as late gadolinium enhancement.

The participation of myocarditis patients in CVR programs after resolution of the acute phase has been the subject of very little study. There is no published research on the safety and effectiveness of this intervention. However, reports of CVR in this patient population have demonstrated benefits in quality of life and physical fitness, especially when there is functional impairment, even after improvement of the acute condition and optimization of drug therapy. ^288 - 290^

Before starting any exercise practice, patients with a history of myocarditis should undergo echocardiography, 24-hour Holter monitoring, and an exercise test no less than 3 to 6 months after the acute phase has resolved. ^269 , 283^ After this evaluation, selected cases may initiate moderate CVR, aiming at the general benefits obtained by patients with HF.

In sports, it is reasonable for athletes to return to their normal training routine only if they achieve: return of systolic function to normal values; markers of myocardial necrosis and inflammation within normal range; and absence of clinically significant arrhythmias on both Holter monitoring and an exercise test. It is noteworthy that the clinical significance of persistent late gadolinium enhancement on MRI in post-myocarditis patients whose clinical symptoms have resolved remains unknown. Thus, it seems reasonable that those with small areas of enhancement and without significant arrhythmias on Holter monitoring and exercise testing can return to sports, provided that clinical monitoring is continued. ^269^

In chronic cases, in which ventricular dysfunction persists throughout the follow-up period, the patient should follow the general recommendations for CVR as described for chronic HF (see Table 6 ).

### 6.6.3. Other Cardiomyopathies

***6.6.3.1. Arrhythmogenic Right Ventricular Cardiomyopathy***

Arrhythmogenic right ventricular cardiomyopathy (ARVC) is an inherited disease that is associated with SCD in young adults and athletes. Pathologically, myocytes are lost and replaced with fibroadipose tissue, especially in the myocardium of the right ventricle, although isolated left ventricular or biventricular involvement may also occur. ^291^

There is evidence, in an experimental animal model, that exercise increases penetrance and risk of arrhythmias in patients with traditional ARVC mutations. ^292^ In individuals with positive genotypes, an increased risk of arrhythmias with exercise has also been confirmed. Ventricular tachyarrhythmias and SCD events in this condition usually occur during exertion, including sports and endurance exercise, with an increased risk of tachycardia, ventricular fibrillation, and HF. ^293^

It has been shown that individuals with ARVC who are involved in competitive sports experience a higher incidence of ventricular tachyarrhythmias and SCD, in addition to earlier symptom onset, compared with those who participated only in light physical activity and those who were sedentary. ^270^ The reduction in exercise intensity was associated with a substantial decrease in the risk of ventricular tachyarrhythmias or death, especially in patients without a detected desmosomal mutation and with an ICD for primary prevention. ^294^ Therefore, the scientific evidence suggests that participation in sports and intense exercise are associated with early onset of symptoms and an increased risk of ventricular arrhythmias and major events in patients with ARVC. Therefore, these patients must be disqualified from participation in sport. ^269 , 276^

Regarding participation in CVR programs, there is no scientific data to indicate or suggest any benefits of physical exercise for patients with ARVC. On the other hand, keeping them sedentary, which contributes to low physical fitness, may also be inappropriate, as there is a general association of low physical fitness with mortality. ^14 , 21^

In a small observational study of patients with ARVC, there was no difference in mortality rate between inactive individuals and those who performed only recreational physical activities. ^270^ Thus, it can be assumed that participation in a supervised CVR program, restricted to exercise of light to moderate intensity, could not be harmful. Depending on other individual clinical characteristics, such as the presence of cardiovascular risk factors, physical exercises could be prescribed to control these conditions.

Therefore, the inclusion of a patient with ARVC in CVR programs should only be carried out after a thorough pre-exercise evaluation and rigorous evaluation of the risk-benefit balance of physical exercises. Options should be discussed with the patient, exposing the absence of proven benefits versus the potential risks of physical inactivity and low physical fitness. It is then up to the patient to choose according to their own personal preferences.

In the context of CVR, extrapolating findings from athletes, a restriction on higher training intensities is also suggested. Patients with ARVC could thus perform supervised physical exercises of light to moderate intensity.

***6.6.3.2. Noncompaction Cardiomyopathy***

Noncompaction cardiomyopathy (NCM) is a heart disease that occurs due to embryonic interruption of myocardial compaction. It is characterized by segmental thickening of the left ventricular walls, consisting of two layers: a compacted epicardial one and an endocardial one with marked trabeculation and deep intratrabecular recesses, where spaces are filled by blood flow. ^295 , 296^

Its incidence and prevalence are uncertain, ranging from 0.02 to 0.05% according to some echocardiographic records. ^297^ Clinically, it can be asymptomatic or present with symptoms of HF, ventricular and/or atrial arrhythmias, pre-excitation, thromboembolic events, or SCD. There are no universally accepted criteria for morphological diagnosis; however, a ratio between noncompacted/compacted myocardium greater than 2.1:1 at the end of systole on echocardiography or 2.3:1 at the end of systole on MRI has become the most widely accepted proposed criterion. ^298^

It is not yet established how physical training can influence NCM, nor is the frequency of development of noncompaction morphology in the population known. ^299 , 300^ In recent studies, athletes have shown a high prevalence of increased ventricular trabeculation when compared to a control group (18.3 *versus* 7%). It is believed that the increase in ventricular trabeculation or the presence of isolated echocardiographic criteria for cardiomyopathy is probably of little significance, and may be part of the spectrum of athlete’s heart. ^300 , 301^ Therefore, not all athletes with isolated ventricular compaction are diagnosed with NCM. Therefore, functional parameters (such as ejection fraction) must also be considered to guide management. ^301^

To date, there is no evidence from studies of CVR or training in NCM. Therefore, patients with left ventricular dysfunction should follow the same exercise recommendations as those with chronic HF (see Table 6 ).

## 6.7. Valvular Heart Disease

Patients with valvular heart disease represent a very heterogeneous group with major variability in terms of age, etiology, affected valves, and severity of involvement, whether due to stenosis, regurgitation/insufficiency, or mixed lesions. However, most valvular heart diseases share a common feature in their clinical manifestations induced by exertion, which include chest pain, dyspnea, and/or functional limitations. The severity of these symptoms in patients with severe valvular heart disease can be used as one of several criteria to indicate surgical or percutaneous intervention. In addition, the identification of reduced aerobic fitness, as documented by CPET or TMET, is also a criteria used to define whether interventions are indicated. ^302 - 304^

One major issue in the clinical follow-up of patients with valvular heart disease is the prolonged natural history of these conditions. The onset and progression of symptoms and functional limitations is often slow, which may lead patients to spontaneously reduce their engagement in physical activity due to symptoms on exertion. This sedentary lifestyle can contribute to further reductions in aerobic physical fitness and worsen symptoms.

Thus, doubts may arise regarding the clinical management and need for interventions when the patient undergoes a CPET or TMET, namely: are limitations in physical fitness identified on exercise testing a result of progressive valvular heart disease, a sedentary lifestyle, or both? In this context, the regular practice of physical exercise and the consequent maintenance or even improvement of physical fitness are important to elucidate these questions in the follow-up of patients with valvular heart disease.

Participation of these patients in CVR programs has been the subject of a single cost-effectiveness study. ^305^ However, increases in the functional capacity of individuals referred for CVR have been demonstrated consistently, ^306 , 307^ which justifies referral to exercise-programs (level of evidence C).

Rehabilitation in the setting of valvular heart disease can be subdivided into two phases: pre- and post-intervention (surgical or percutaneous).

### 6.7.1. Pre-Intervention Phase

Patients with moderate to severe valvular heart disease in the pre-intervention phase are rarely enrolled in CVR programs. Training is carried out mainly in asymptomatic cases, in whom there is still no indication for valve repair or correction.

CVR can be useful to keep the patient physically active while waiting for future intervention; after all, a sedentary lifestyle can deteriorate functional capacity and, thus, increase the risk of postoperative complications, especially when the intervention is performed in older adults with multiple comorbidities and established frailty. ^308 - 310^

In addition, monitoring during supervised CVR sessions can be useful to observe changes in symptoms and physical fitness, which can indicate progression of valvular heart disease and suggest the need for medical reevaluation.

### 6.7.2. Post-Intervention Phase

Post-intervention patients are more common in CVR programs, as structured and supervised exercise is a useful means of observing the hemodynamic behavior of a patient’s new (or newly repaired) valve. Information on a patient’s response to physical exercise can help their primary physician adjust drug therapy and/or review valve function. In addition, supervised exercise provides a greater measure of safety for the patient to return to his or her activities of daily living, leisure, and sports.

Although there is no consensually defined time limit for referral to CVR in the setting of valvular heart disease, the earlier the patient starts exercise, the less function he or she will lose from inactivity. ^305 - 307 , 310^ The exchange of information between the patient’s primary physician and the rehabilitation physician is the best strategy for defining the optimal timing of referral, and the pre-exercise evaluation has a fundamental role in consolidating this shared decision.

### 6.7.3. Pre-Exercise Evaluation

The pre-exercise evaluation should always consist of a thorough history, physical examination, and evaluation of laboratory tests and imaging. The clinical history must include: length of hospital stay; complications related to the procedure, such as pleural or pericardial effusion, mediastinitis, and infections; type and size of prosthetic valve; surgical technique; and whether CABG was performed concomitantly, in addition to other clinical information that may be relevant regarding other comorbidities.

On physical examination, cardiac and pulmonary auscultation are particularly important. In addition, attention should be paid to the surgical scar, which should be examined for signs of inflammation and infection, sternal instability, and pain or discomfort on palpation. If concomitant revascularization was performed, the saphenectomy and/or radial artery donor site must be examined. If valve repair or replacement was performed percutaneously, the access site should be checked for signs of peripheral vascular complications.

It is important that the clinician look for signs of anemia on physical examination and laboratory tests, because this is a common complication and can have a negative impact on functional capacity. ^311^ Laboratory evaluation of coagulation is relevant in patients who received a mechanical valve and were started on anticoagulants. Achieving the correct level of anticoagulation is important in preventing complications.

A resting ECG should be obtained to check for any arrhythmias and disturbances in rhythm or conduction. The most commonly used imaging modality in the evaluation of valvular heart disease is Doppler echocardiography, which allows assessment of ventricular function and cavity dimensions, measurement of transvalvular pressure gradients, estimation of pulmonary artery systolic pressure, and measurement of blood flow, which provides a good overview of valve function and cardiac function at rest. Echocardiography should always be performed before the start of a CVR program, to assess the risk of exercise-related complications. ^312^

It is important to evaluate functional capacity by CPET or TMET. ^313 - 316^ These tests, especially CPET, provide extremely useful information regarding aerobic fitness and the hemodynamic repercussions of valvular heart disease, which may be underestimated by assessments performed at rest. In addition, treadmill tests identify parameters that are used to guide exercise prescription and restrictions. When TMET and CPET are unavailable, the use of functional tests, such as the 6-minute walk test and the step test, should be considered. ^317 - 320^

It is important to emphasize that CPET and TMET pose greater risk in patients with stenotic lesions; therefore, they should only be carried out by experienced physicians and in a safe setting with the necessary infrastructure to respond in case of emergency. ^321^

Functional tests are indicated not only in pre-exercise evaluation, but also to elucidate any doubts regarding the symptoms of patients in the pre-intervention phase of valvular heart disease. The combination of functional tests with echocardiography helps assess the response of the transvalvular pressure gradient and pulmonary artery systolic pressure to exertion, especially when there is a discrepancy between echocardiogram findings at rest and clinical signs and symptoms. ^304 , 322 , 323^

Another relevant issue is the evaluation of elderly patients, who are frequently affected by valvular heart disease and have a high prevalence of risk factors and comorbidities. ^324^ Due to their high surgical risk, such patients are now considered candidates for percutaneous repair or replacement of the aortic ^325^ and mitral valves. ^326^ In this scenario, CVR can be considered before the intervention, with the aim of decreasing complication rates, length of hospital stay, and mortality and morbidity associated with the frailty syndrome. ^327^ After the intervention, CVR then provides an opportunity for monitoring and optimization of the outcomes of the procedure in all its aspects. ^328 - 331^

The use of frailty syndrome assessment instruments is still a controversial subject in the literature; there is no consensus regarding the best protocol to assess CVR outcomes. The assessment should include objective tests and instruments to address risk in several domains: mobility, muscle mass and strength, independence in activities of daily living, cognitive function, nutrition, anxiety, and depression. ^304 , 308 , 332^

### 6.7.4. Special Considerations for the Prescription and Follow-Up of Physical Exercise Programs

This section will only address guidelines and recommendations for exercise in patients with moderate or severe valvular heart disease, as there are no restrictions to exercise in patients with mild involvement. Participation in competitive sports should follow the recommendations of the specific literature on the subject. ^276 , 333 , 334^ Scientific evidence is scarce as to the impact of regular exercise on the progression of valvular heart disease and its complications; therefore, recommendations are based on expert opinion alone (level of evidence C).

Acutely, exercise causes an increase in adrenergic tone and in the hemodynamic load imposed on the cardiovascular system, which raises concerns regarding the potential for deleterious cardiovascular effects in patients with valvular heart disease, including progression of aortic disease, functional deterioration, pulmonary hypertension, cardiac remodeling, myocardial ischemia, and arrhythmias.

Patients with valvular heart disease who will start a CVR program must undergo a stress test to guide exercise prescription. Table 9 summarizes recommendations for asymptomatic patients, who have not undergone any intervention, with moderate or severe valvular heart disease. In general, training will consist of a combination of aerobic and resistance exercise. When there are no restrictions, the recommendations for exercise prescription will be the same as those for individuals without heart disease.

**Table 9:** – Physical exercise in asymptomatic individuals with valvular heart disease

Valvular heart disease	Aerobic exercise	Resistance exercise
Aortic insufficiency	**Moderate or severe** (normal ventricular function; LVESD < 50 mm in men or < 40 mm in women; good functional capacity) **No restrictions**	**Moderate or severe Avoid high intensity**
Aortic stenosis	**Moderate or severe** (normal ventricular function; good functional capacity; absence of myocardial ischemia, complex ventricular arrhythmias, or plateau response/fall in SBP) **Avoid high intensity**	**Moderate** Avoid high intensity **Severe** Limited to low intensity (enough to maintain activities of daily living)
Mitral insufficiency	**Moderate or severe** (normal ventricular function; LVEDD < 60 mm; PASP < 30 mmHg) **No restrictions**	**Moderate or severe** Avoid high intensity
Mitral stenosis	**Moderate or severe** (good functional capacity) **Avoid high intensity**	**Moderate or severe** (good functional capacity) **Avoid high intensity**

*LVEDD: left ventricular end-diastolic diameter; LVESD: left ventricular end-systolic diameter; SBP: systolic blood pressure; PASP: pulmonary artery systolic pressure.*

For symptomatic patients in whom surgical correction is not indicated or who do not have the characteristics described in Table 9 , the intensity of exercise should be limited by the occurrence of abnormalities observed during the CPET or TMET, as it is assumed that repeated insults at this intensity could increase the risk of exercise and induce potential deleterious effects in the long term. The exercise prescription should be limited to an intensity of exertion corresponding to 10 bpm below the HR at which the abnormality occurred during the CPET or TMET. Relative loads and subjective perceived exertion can be used to guide exercise prescription when HR is not a good indicator, such as in patients with atrial fibrillation or an artificial pacemaker ( Table 10 ).

**Table 10:** – Abnormalities observed on cardiopulmonary exercise test or treadmill exercise test that should limit exercise intensity in patients with valvular heart disease

Exercise-induced changes	Description
Signs and symptoms	Onset of angina, angina equivalent, or other signs and symptoms indicative of exercise intolerance
Blood pressure	Plateau response or decline in SBP; or, SBP >220 mmHg; or, DBP >115 mmHg
ST segment	Onset of ST segment depression (horizontal or descending) >1 mm
Ventricular function	Evidence of decreased ventricular function on exertion or onset of moderate to major left ventricular wall motion abnormalities
Pulse O _2_ (CPET only)	Evidence of early plateau or decline on effort despite increased load
Arrhythmia	Grade 2 or 3 AV block, atrial fibrillation, supraventricular tachycardia, complex ventricular arrhythmias

*CPET: cardiopulmonary exercise test; SBP: systolic blood pressure; DBP: diastolic blood pressure, AV: atrioventricular.*

In patients who have undergone surgical correction of valvular heart disease, the exercise prescription will depend on the underlying disease, the outcome of the procedure, the presence of residual lesions, ventricular function, and the response to the exercise test (TMET or CPET). Therefore, each case must be assessed individually, and the limits of exercise prescription defined by the pre-exercise assessment and the results of physical examination and any other tests performed.

## 6.8. Patients with Artificial Pacemakers or Implantable Cardioverter-Defibrillators

This section describes particulars involving implantable devices: artificial pacemakers and implantable cardioverter-defibrillators (ICD). Artificial pacemakers are indicated in the management of electrical abnormalities, which may be isolated – sick sinus syndrome, advanced atrioventricular (AV) block – or associated with structural heart diseases. ICDs are indicated for the primary or secondary prevention of SCD in patients with severe electrical and/or structural heart disease. Depending on the underlying heart condition, the recommendations on CVR described elsewhere in this guideline apply.

One of the main concerns of physical exercise in patients with an artificial pacemaker or ICD is the risk of device-related complications, especially in high-impact activities. In patients with ICDs, there is the added fear of inadvertent activation, which can cause behavioral changes, such as reduced physical activity and participation in moderate-intensity exercise. ^335 , 336^ Health care providers also share these fears, ^337^ which may limit their exercise prescribing practices. However, studies have shown that physical exercise is safe and is not associated with an increased risk of shocks or other adverse events. ^338 - 342^ In addition, ICD-related complications have not been observed even in competitive athletes. ^343 , 344^

Nevertheless, before clearing a patient for exercise, the clinician must be aware of the reasons for device placement and become familiar the device’s programming parameters and settings, ideally during the pre-exercise evaluation.

### 6.8.1. Therapeutic Benefits of Physical Exercise

A meta-analysis ^342^ of 14 studies enrolling 2,681 patients with ICDs showed a beneficial effect of physical exercise on functional capacity in this population, with an average increase in VO _2_ of 2.4 ml.kg ^- 1^ .min ^- 1^ . In another meta-analysis, which included five randomized trials and one nonrandomized study in patients with HF and ICDs, ^341^ a similar improvement in physical capacity was observed, with an increase in VO _2_ peak of 1.98 ml.kg ^- 1^ .min ^- 1^ in relation to the control group.

As for the concern of inadvertent ICD activation during physical training, one meta-analysis found no significant differences. The rate of exercise-associated shocks ranged from 0 to 20% across studies, with an average of 2.2%, similar to the rate of shocks during an exercise-free follow-up period. ^342^ Thus, despite widespread fear of this phenomenon, physical training was not associated with increased ICD activation and proved safe.

Another meta-analysis actually reported a lower likelihood of shocks during follow-up in patients participating in CVR compared to controls, corroborating the previous result of an observational study, which reported a higher incidence of ICD activation in patients who did not participate in CVR programs. ^341 , 345^

One possible explanation or the lower incidence of arrhythmias and shocks in patients undergoing CVR would be the improvement of physical capacity, as it has been previously documented that greater physical fitness is associated with a lower incidence of arrhythmia. ^16 , 17 , 346^ In addition, exercise could reduce myocardial arrhythmogenicity due to remodeling and reduction of sympathetic excitability. ^347^

In a nationwide study with 10 years of follow-up which included 150 patients with ICDs in a CVR program, all of which completed a CPET or TMET to support exercise prescription, there were only three shock events and all were appropriate. ^348^ This provides additional evidence of the safety of stress testing and CVR in this population.

### 6.8.2. When Is Cardiovascular Rehabilitation Indicated?

Physical exercise can and should be indicated as long as the patient’s clinical condition is stable and clinical treatment is optimized. In addition to the potential beneficial effects on underlying heart disease, CVR increases physical fitness and can help reduce the incidence of arrhythmias and, consequently, of ICD activation ( Table 11 ).

**Table 11:** – Indications for physical exercise and other treatments in patients with implantable cardioverter–defibrillators

Indication	Recommendation	Level of evidence
Physical exercise to increase physical capacity in stable patients with an ICD ^341,342^	I	A
Physical exercise for potential reduction of the incidence of ICD activation (shocks) ^341^	IIa	B
Use of neuromuscular electrical stimulation in patients with implantable devices with bipolar sensors, when performed on muscles far from the implant site ^349^	IIb	B

*ICD: implantable cardioverter–defibrillator. If any structural heart disease is present, the corresponding recommendations should be taken into account.*

### 6.8.3. Pre-Exercise Evaluation

In patients with implantable devices, the clinician must become familiar with the reason for implant placement, the patient’s ventricular function, whether any arrhythmias are present and, particularly, the device settings and parameters. For patients with an artificial pacemaker, this means understanding the programming mode, the set HR limits, and the type and response of the activity sensor. In patients with an ICD, essential information includes the HR threshold which has been set to trigger shock or burst therapies.

In addition to the standard clinical examination, pre-exercise evaluation is of paramount importance in these patients. Ideally, a CPET or TMET should be performed to determine functional capacity and analyze the behavior of the device during exertion. However, the impossibility of performing CPET or TMET should not prevent the practice of physical exercise. In these cases, monitoring during sessions may reveal a need for device reprogramming, usually of maximum HR and sensor response settings.

During CVR sessions, continuous ECG monitoring can be achieved with the use of telemetry systems. HR control devices, such as regular cardiac monitors, can also be used for monitoring CVR sessions. ^350^ However, due to changes in the ECG tracing caused by artificial pacing, automated HR measurement both by ECG telemetry systems and by cardiac monitors may be erroneous. The team should be aware of this potential for error and measure HR manually as needed.

### 6.8.4. Special Considerations for the Prescription and Follow-Up of Physical Exercise Programs

When prescribing and defining intensity limits for aerobic physical training, one should be aware of ICD programming and limit the intensity accordingly to 10–20 bpm below the HR set to trigger therapy (shock or burst). This is especially important in young individuals who experience elevated HR during training. In older patients with HF who are on high-dose beta blockers, the peak HR observed during CPET or TMET is usually below the threshold that triggers ICD therapy.

Patients with an artificial pacemaker may have different chronotropic responses observed on CPET or TMET, which will impact the prescription of aerobic exercise. In addition, the individual’s own pace, type of pacemaker, and presence of a rate sensor will influence the HR response to exertion and, consequently, the exercise prescription. ^351^

The four possible types of artificial pacemaker response to exertion are as follows:

**1) Normal or depressed sinus-node chronotropic response. Pacemaker inhibited (not triggered).** The chronotropic response to stress is mediated by sinus rhythm and may be normal or depressed (due to sinus node dysfunction and/or drug effect). Ventricular conduction occurs via the own pathway, and the pacemaker is not triggered on exertion. In some cases, it can be triggered at rest and during initial loads, with atrial and/or ventricular pacing. However, during exertion, the pacemaker is inhibited, with a predominance of sinus responses and ventricular conduction via the own pathway. In this type of response to exertion, the intensity of exercise prescription should be based on the usual concerns and is entirely unaffected by the presence of an artificial pacemaker.

**2) Normal or depressed sinus-node chronotropic response. Pacemaker triggered (activity-initiated ventricular pacing).** The chronotropic response to exertion is mediated by the sinus rhythm. Sinus activity is sensed by the pacemaker and triggers synchronized ventricular pacing according to preset paced atrioventricular intervals. In this case, if the maximum pacemaker response limit has been set appropriately for the patient’s sinus response, the exercise prescription may be HR-based, as the ventricle will be paired with sinus activity. However, if the maximum pacemaker response limit is set lower than the patient’s sinus response, dyssynchrony of ventricular pacing and sinus activity will occur at moderate to high exercise intensity. The pacemaker will then block some sinus stimuli by mimicking AV-node Wenckebach activity, a phenomenon known as “electronic Wenckebach”, ^352^ in order to keep the ventricular HR within the programmed limit; a plateau in the chronotropic response to exertion will ensue. In this scenario, the loss of synchrony between sinus rhythm and ventricular rate will interfere with the utility of HR to guide exercise intensity. The exercise prescription should instead be based on relative loads and/or subjective perceived exertion.

When the electronic Wenckebach phenomenon occurs, extreme care is required to detect it during CPET or TMET. It is essential to obtain precise information on the atrial rate at which the pacemaker will initiate 2:1 block, because as this rate is reached, ventricular pacing will occur at a 2:1 ratio, with the potential for a sudden fall in HR on exertion and an abrupt, symptomatic reduction in cardiac output. Therefore, unless the programmed Wenckebach interval and the 2:1 block rate are quite far apart, the HR which triggers electronic Wenckebach may be used as the upper limit for CPET or TMET, as well as for the exercise prescription.

In such cases, pacemaker reprogramming to better match the patient’s sinus response should be considered and discussed with the primary physician. Another option, depending on the clinical picture, is the optimization of drug therapy with negative chronotropic agents (such as beta blockers). A reduced sinus response may prevent the aforementioned event.

**3) Fixed, pacemaker-mediated chronotropic response (no rate responsive pacing).** Some patients may have no sinus activity at all, as in atrial fibrillation. In these cases, individuals with complete AV block will be completely dependent on ventricular pacing. If the pacemaker has no rate responsive pacing, or if the sensor is disabled, there will be no chronotropic response to exertion; the pacemaker will be set to a fixed HR. This type of pacemaker and programming is now exceedingly rare. Nevertheless, in such patients, the HR is useless to guide exercise prescription, which should instead be based on relative loads and/or subjective perceived exertion.

**4) Pacemaker-mediated chronotropic response (rate responsive pacing).** In patients with atrial fibrillation and AV block, as previously described, but whose artificial pacemaker has an active sensor with rate responsive pacing, there will be dependence on ventricular pacing, but activation of the sensor by exertion will lead to a pacemaker-mediated chronotropic response. In patients with sinus rhythm, but with a large chronotropic deficit due to sinus node dysfunction and/or drug effects, a chronotropic response to exertion may also occur, mediated by the pacemaker sensor, with atrial pacing followed or not by ventricular pacing.

The speed and magnitude of the rate sensor’s response to exertion are programmable, with the possibility of adjusting the sensor activation threshold, the rate of increase in HR to exertion and the rate of reduction during recovery, and the maximal HR limit for the sensor. TCPE or TMET can be used to verify the adequacy of the response and identifying potential needs for pacemaker reprogramming, which should be discussed with the patient’s primary physician.

In such cases, as the chronotropic response will be artificially mediated by the device, HR-based prescription of exercise intensity may be inaccurate. Therefore, the use of relative loads and/or perceived exertion is preferred.

Pacemakers with accelerometer sensors and axial motion detection, which are the most common, have a sensitive response to walking or running on a treadmill. However, as there is no vertical movement on a cycle ergometer, the sensor is activated very little or not at all. As a result, there is inferior chronotropic response during ciclo ergometer exercise, which may vary according to the individual response of the patient.

### 6.8.5. Resistance Training

Resistance training is an important component of CVR for several heart diseases. However, after implantation of a pacemaker of ICD, some care is required until healing is complete, to prevent vascular injury, displacement of the device, and electrode fracture. For instance, caution is recommended when performing weight training and any exercise which involves raising the arms during the first 6 weeks after the implant procedure. In addition, repetitive and intense movements of the limb ipsilateral to the device should be avoided.

However, such guidelines are geared more at patients involved in sports, and are unlikely to be relevant to the exercises carried out in CVR programs. A study of early, supervised shoulder mobilization immediately after artificial pacemaker implantation did not observe any device complications. ^353^

### 6.8.6. Neuromuscular Electrical Stimulation

The use of neuromuscular electrical stimulation (NMES) in patients with HF has become widespread, especially for those unable to exercise due to disease severity. NMES can improve aerobic capacity, muscle strength, and cross-sectional area of the quadriceps muscles, and is an effective passive exercise modality in this population. ^354 - 356^ However, the use of electronic devices in these patients (ICDs, artificial pacemakers, resynchronization devices) is also increasing, which raises concerns of the possibility of electromagnetic interference.

A systematic review ^349^ demonstrated that NMES of the quadriceps muscles appears to be safe and feasible in patients with HF and a bipolar sensing ICD. However, the review itself notes that the number of studies and patients included is too small to allow more comprehensive conclusions, and concludes that NMES can be used provided the following conditions are met:

1. If individual hazards (pacemaker dependence, acute HF, unstable angina, ventricular arrhythmia in the last 3 months) have been excluded before starting NMES.
2. If NMES is performed only on the quadriceps and gluteal muscles.
3. If treatment is regularly supervised by a doctor, and the device is analysed after every NMES session.

Therefore, at the present time, although NMES seems safe to use in patients with bipolar-sensing implantable devices when performed on muscles far from the implant, there is still a need for studies with a larger number of patients to confirm that use is safe and feasible without the need for repeated, detailed device evaluation after sessions.

## 6.9. Peripheral Arterial Occlusive Disease

Stroke has been correctly viewed and addressed as a serious disease with massive impact on public health. However, peripheral artery disease, which is also highly prevalent worldwide and carries high morbidity and mortality rates, affecting more than 40 million individuals in Europe alone, have not been properly addressed, hindering prevention, diagnosis, and effective treatment. ^357 , 358^ In this context, peripheral arterial occlusive disease (PAOD) of the lower limbs is particularly concerning, as, at its most severe stage (critical ischemia), it is associated with a high risk of cardiovascular events, lower limb amputation, and death. With the growth of risk factors such as age, diabetes, and smoking, critical ischemia of the lower limbs has become more prevalent, and currently affects about 2 million individuals in the United States alone. ^359^

The presence of PAOD is suspected when there is pain in the lower limbs on exertion, with no apparent musculoskeletal etiology, and the ankle-brachial index (ABI) is <0.90 at rest. ^360 , 361^ The ABI has been recommended as a diagnostic resource prior to use of imaging. ^362^ Functional tests during exertion effort may be necessary to establish the diagnosis, especially when the ABI is greater than 0.91, as well as for functional classification and exercise prescription in CVR.

Gait can be assessed by means of field tests, which allow the diagnosis of intermittent claudication and determination of the distance walked until onset of symptoms (initial claudication) and until development of total loss of function (absolute claudication).

A TMET with measurement of the ABI at rest and after exercise has also been proposed as a diagnostic test. The presence of PAOD is suggested by a greater than 20% reduction in post-exercise ABI as compared to resting values, or a decrease in post-exercise BP greater than 30 mmHg as compared to the resting state. ^363^ Another study reported lower cut-off scores, with PAOD being suggested when there is a greater than 18.5% reduction in ABI and a greater than 15 mmHg decrease in BP after exercise. ^364^

Considering the overall cardiovascular risk of these patients, optimized clinical treatment should always be instituted. In addition, smoking cessation and drug therapy with statins and antiplatelet agents must be considered, as well as adequate blood glucose and BP control. Regarding the use of cilostazol, there is no consensus among the guidelines of different medical societies. ^362 , 363^

In symptomatic patients, exercise has the potential to influence morbidity and mortality, reducing symptoms, improving quality of life, and increasing the maximum walking distance ( Table 12 ). ^365^ Physical activities performed under direct supervision have been shown to be more effective than unsupervised exercise. ^366^ In 14 clinical trials (1,002 participants), with an intervention duration from 6 weeks to 12 months, pain-free walking increased about 180 meters more in training under direct supervision when compared to training under indirect supervision. Physical training is safe; in most studies, the sole exercise was walking to claudication, at least three times a week, for at least 3 months. ^367^

**Table 12:** – Treatment of peripheral arterial occlusive disease of the lower limbs

Indication	Recommendation	Level of evidence
Supervised physical exercise to improve function and quality of life and reduce symptoms of claudication ^365,369,375,376^	I	A
Home-based physical exercise or other training modalities to improve functional status ^366,370,371^	IIa	A
In symptomatic patients, a supervised physical exercise program should be discussed as a treatment option before revascularization ^375,376^	I	B

In patients with PAOD, training under direct supervision is also superior in terms of cost-effectiveness, ^368^ although indirect supervision (HBCR) is a good alternative, with positive effects on quality of life and significantly greater improvement in walking tolerance as compared to a simple recommendation to walk. ^369 , 370^

When walking is not feasible, other activities, such as cycling, resistance training, and use of an upper body ergometers, have also proven effective. ^371^ It is also worth noting that physical exercise is contraindicated in patients with critical ischemia, but should be considered as soon as possible after successful interventional treatment. ^371 - 373^

A systematic review of 12 clinical trials including a total of 1,548 patients compared patients who received drug therapy with physical training, endovascular intervention, and open surgery for treatment of claudication. All modalities increased walking distance, reduced symptoms, and improved quality of life. ^374^ Endovascular intervention and open surgery have proven effective in relieving symptoms, increasing walking distance, and improving quality of life, and are indicated when severe symptoms that negatively influence daily life persist despite full clinical treatment (physical exercise and optimized drug therapy).

In a randomized clinical trial of 111 patients with aortoiliac disease, the increase in exercise time on a graded treadmill test was greater in the supervised exercise group than in the stent revascularization group. ^375^ However, after 18 months of follow-up, the functional and quality of life benefits were equivalent in the exercise and revascularization groups, and, in both cases, were superior to those in the group that received medication alone. ^376^

Several clinical trials have compared the efficacy and effectiveness of supervised physical exercise, angioplasty, and optimized medical care, using a multitude of different designs. Most trials consisted of two treatment arms. The aforementioned systematic reviews suggested that supervised physical exercise may be superior to optimized medical care or angioplasty. However, these meta-analyses included head-to-head comparisons between two specific treatment arms (e.g., angioplasty versus supervised physical training) or used an approach that did not allow inclusion and direct comparison of all available treatments for intermittent claudication. ^377^

For these reasons, a recent meta-analysis sought to establish comparisons between all available treatments in order to elucidate the best management of patients with symptomatic PAOD. The sample included 2,983 participants with intermittent claudication (mean age, 68 years), 54.5% of whom were male. The comparisons were optimized medical care (n = 688), supervised physical training (n = 1,189), angioplasty (n = 511), and angioplasty plus supervised physical training (n = 395). The mean follow-up period was 12 months. Compared with optimized medical care alone, angioplasty and supervised physical training outperformed all other therapeutic strategies, with a 290 m gain in maximum walking distance (95% CI: 180 to 390 m; p < 0.001), corresponding to a proportional gain of 141% (95% CI: 86.85 to 188.3%; p < 0.001), with an average follow-up period of 12 months. ^378^

Supervised physical training alone and angioplasty plus supervised physical training again surpassed the other treatment modalities, with an additional gain of 110 m in maximum walking distance (95% CI: 16 to 200 m; p < 0.001), or a proportional gain of 66% (95% CI: 9.66 to 121%; p < 0.001). Supervised physical training alone yielded a 180-m gain in maximum walking distance (95% CI: 130 to 230 m), corresponding to a proportional gain of 87% (95% CI: 63 to 111%); this was higher than with angioplasty alone, but lower than with supervised physical training plus angioplasty, in terms of maximum walking distance. ^378^

These review studies have important implications for clinical practice. This is because all patients with intermittent claudication should receive optimized clinical treatment, in view of the evidence that shows a reduction in future cardiovascular events and an improvement in limb-related outcomes. ^379 , 380^ In this context, supervised physical training and angioplasty are essential to improve walking distance and quality of life. This recent meta-analysis cited above strongly suggests that supervised physical training associated with angioplasty should be part of first-line treatment, always in the context of optimized drug therapy. The offer of angioplasty without optimized physical training should be avoided whenever possible. ^378^ However, DAOP treatment centers often offer angioplasty primarily due to the lack of centers focused on supervised physical training. Furthermore, it cannot be neglected that supervised physical training faces resistance on the part of patients themselves, who are often little adherent to treatment, which partly justifies the majority preference in favor of percutaneous treatment. ^381^

However, recent studies have demonstrated the benefits of combining treatment modalities for symptomatic PAOD, which may increase the likelihood that CVR will become increasingly widespread and accessible. ^378 , 382^

Thus, in addition to optimized medical care, angioplasty combined with supervised physical training seems to be the ideal strategy for initial treatment of patients with intermittent claudication, both to improve maximum walking distance and to improve quality of life. However, the data from these latest reviews cannot confirm whether supervised physical training should be followed by angioplasty or vice versa.
